# Analysis of the Genes from Gibberellin, Jasmonate, and Auxin Signaling Under Drought Stress: A Genome-Wide Approach in Castor Bean (*Ricinus communis* L.)

**DOI:** 10.3390/plants14081256

**Published:** 2025-04-20

**Authors:** Ygor de Souza-Vieira, Esther Felix-Mendes, Gabriela Valente-Almeida, Thais Felix-Cordeiro, Régis L. Corrêa, Douglas Jardim-Messeder, Gilberto Sachetto-Martins

**Affiliations:** 1 Departamento de Genética, Instituto de Biologia, Universidade Federal do Rio de Janeiro, Rio de Janeiro 21941-902, Brazil; ygor.vieira3126@gmail.com (Y.d.S.-V.);; 2 Institute for Integrative Systems Biology (I2SysBio), Consejo Superior de Investigaciones Cientificas (CSIC), Universitat de València (UV), 46980 Valencia, Spain; 3 Programa de Biologia Molecular e Biotecnologia, Instituto de Bioquímica Médica Leopoldo de Meis, Universidade Federal do Rio de Janeiro, Rio de Janeiro 21941-902, Brazil

**Keywords:** phytohormones, GA, JA, Aux, water stress, phytohormone crosstalk

## Abstract

Castor bean (*Ricinus communis* L.) can tolerate long periods of dehydration, allowing the investigation of gene circuits involved in drought tolerance. Genes from gibberellins, jasmonates, and auxin signaling are important for crosstalk in the developmental and environmental adaptation process to drought conditions. However, the genes related to these signals, as well as their transcription profiles under drought, remain poorly characterized in the castor bean. In the present work, genes from gibberellins, jasmonates, and auxin signaling were identified and molecularly characterized. These analyses allowed us to identify genes encoding receptors, inhibitory proteins, and transcription factors from each signaling pathway in the castor bean genome. Chromosomal distribution, gene structure, evolutionary relationships, and conserved motif analyses were performed. Expression analysis through RNA-seq and RT-qPCR revealed that gibberellins, jasmonates, and auxin signaling were modulated at multiple levels under drought, with notable changes in specific genes. The gibberellin receptor *RcGID1c* was downregulated in response to drought, and *RcDELLA3* was strongly repressed, whereas its homologues were not, reinforcing the suggestion of a nuanced regulation of gibberellin signaling during drought. Considering jasmonate signaling, the downregulation of the transcription factor *RcMYC2* aligned with the drought tolerance observed in mutants lacking this gene. Altogether, these analyses have provided insights into hormone signaling in the castor bean, unveiling transcriptional responses that enhance our understanding of high drought tolerance in this plant. This knowledge opens avenues for identifying potential candidate genes suitable for genetic manipulation in biotechnological approaches.

## 1. Introduction

Drought is a significant challenge faced by plants, deeply impacting global crop production, particularly in developing nations [1]. Agricultural production is already under pressure due to freshwater shortages, limited arable land, and an increasing population. Climate change exacerbates these issues, driving us toward a hotter and increasingly arid world [2]. According to the Intergovernmental Panel on Climate Change, land surface temperatures have risen by approximately 1.59 °C, resulting in heightened evaporation and transpiration [3].

Plants are sessile organisms, and strategies for surviving under different environmental stimuli have evolved. Plants synthesize several phytohormones to mediate their growth, immunity, and development [4]. The responses triggered by plant hormones lead to changes in gene expression, protein accumulation, enzyme activity, changes in ion homeostasis, and cellular metabolite levels [5,6]. To survive under different environmental conditions, the crosstalk among phytohormones plays a key role and coordinates signal transduction pathways, which are essential for stress responses [4,7].

Abscisic acid (ABA) plays a key role in the abiotic stress response [2], and the ABA signaling genes have been previously identified in castor bean [8]. ABA is primarily associated with osmotic stress, such as drought, and coordinates a complex response mechanism that also involves interactions with other phytohormone signals, including gibberellin (GA), jasmonic acid (JA), and auxin (Aux) [9].

**Figure 1:**
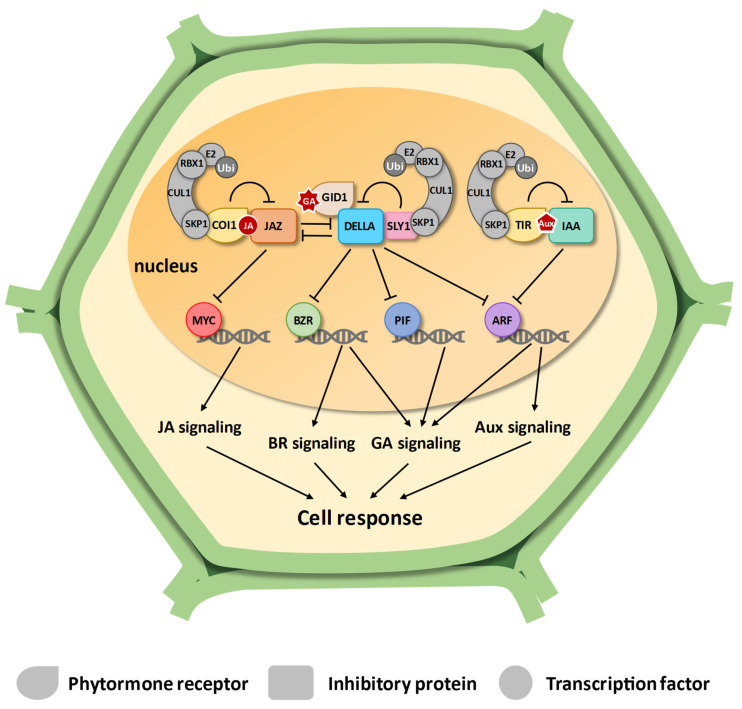
Schematic representation of jasmonate, gibberellin, and auxin signaling. Jasmonic acid (JA), gibberelic acid (GA), and auxin (Aux) are detected by specific receptors (COI, GID1, and TIR, respectively), which inhibit repressor proteins (JAZ, DELLA, and IAA, respectively) by recruiting the SCF ubiquitination complex to target them for degradation via proteasome. This degradation releases transcription factors (MYC, BZR, PIF, and ARF), enabling them to regulate gene expression effectively. In jasmonate and auxin signaling, the receptors are F-box proteins that are already part of the SCF complex. For gibberellin signaling, the F-box proteins SLY/SNE provide specificity by binding to DELLA proteins.

GAs regulate key processes in plant development [10]. The canonical GA signaling begins upon the recognition of GA by the GIBBERELLIN INSENSITIVE DWARF 1 (GID1) receptor [11]. When the interaction with GAs occurs, GID1 undergoes a conformational change that increases its affinity for DELLA proteins. DELLAs are inhibitory proteins that impair GA signaling by interacting with transcription factors that drive GA transcriptional changes [12]. In Arabidopsis, the GA-GID1-DELLA complex is recognized by SCF^SLY1/SNE^ ubiquitin ligase that targets DELLA to proteasome degradation [13] (Figure 1). An important class of transcription factors in the developmental response mediated by GAs is the Phytochrome Interacting Factors (PIF). GA levels and light regulate the amount of elongation growth through PIF and DELLA proteins [14]. In Arabidopsis, Auxin Response Factor 6 (AtARF6) and BRASSINAZOLE-RESISTANT 1 (AtBZR1) were also inhibited by DELLA proteins, and the BZR-ARF-PIF/DELLA (BAP/D) mediates the cooperative regulation of cell elongation by GA, Aux, brassinosteroid (BR), and light signals during seedling photomorphogenesis [15]. GAs and their crosstalk with other phytohormone signaling stimulate developmental process and lead to the activation of genes required for developing strategies to respond to different stresses, including drought [16,17].

Similar signaling systems occur in other phytohormones, such as JA and Aux. The JA receptor is an F-box protein called CORONATINE INSENSITIVE 1 (COI1) linked to a Jasmonate ZIM-domain (JAZ), which inhibits the transcription factors related to JA response. In Arabidopsis and rice, the complex SCF^COI1^ interacts with active JA molecules and sends the JAZ protein to the proteasome, thus releasing previously repressed transcription factors [18,19,20] (Figure 1). The basic Helix-Loop-Helix (bHLH) protein MYC2 is the main responsive transcription factor in JA responses in Arabidopsis [21]. JA plays an important role in regulating biotic and abiotic stresses, as previously demonstrated in Arabidopsis [5,22], rice [21], and other species [23]. The activation of JA defense response is known to severely restrict plant growth, establishing a growth-defense trade-off [7,24].

A significant crosstalk between JA and GA signaling has been described in Arabidopsis and rice, primarily characterized by antagonistic effects. DELLA proteins can physically interact with JAZ in a GA-mediated mechanism that downregulates the JA defense responses [7,25] (Figure 1).

The Aux-mediated signaling occurs similarly to jasmonate signaling. In the presence of Aux, the F-box proteins TRANSPORT INHIBITOR RESPONSE 1 (TIR1) and Auxin Signaling F-box (AFB) protein interact with Auxin/Indole-3-Acetic Acid (Aux/IAA) inhibitory protein. This interaction results in the Aux/IAA ubiquitination and degradation, releasing Auxin Response Factor (ARF), a major class of transcription factors in Aux signaling [26] (Figure 1). Aux plays an important role in plant growth [27]. Aux also assumes a significant role in mediating plant responses to environmental stress conditions, such as osmotic stress imposed by salinity, drought, and low temperature [28].

Castor bean exhibit remarkable drought tolerance [29]. While many genes involved in phytohormone signaling have been identified in various plant species [13,30,31,32,33,34,35,36,37,38,39,40], the specific genes governing the phytohormone signaling under drought in castor bean are still poorly characterized. Moreover, the castor bean seeds are renowned for their rich oil content, comprising approximately 50% of the total biomass [41]. This oil has diverse industrial applications, primarily in pharmaceuticals, cosmetics, lubricants, and biodiesel production [42]. To better comprehend hormonal signaling in castor bean, we employed genome-wide approaches to characterize the core of GA (GID1, DELLA, SLY/SNE, PIF, and BZR), JA (COI, JAZ, and MYC), and Aux signaling (TIR, IAA, ARF) and evaluate their response to drought.

A wide spectrum of the primary structures of protein, as well as phylogeny, and analysis of chromosomal distribution and the duplication events of these gene families were performed. To explore the castor bean response to drought, the expression profile of the identified genes was analyzed in leaves and roots, allowing us to identify genes modulated by drought. We also conducted an analysis of the *cis-*element profile within the promoter regions and miRNAs that can target the identified genes. Collectively, these data enhance our knowledge about the GA, JA, and Aux signaling genes in castor bean and their expression under drought conditions. This knowledge is important for shedding light on the mechanisms underlying the castor bean’s remarkable ability to efficiently adapt to drought stress.

## 2. Results

### 2.1. Genome-Wide Identification and Phylogenetic Analysis of GAs, JA, and Aux Signaling Genes

The analysis of the castor bean genome using protein sequences from Arabidopsis (*Arabidopsis thaliana*) [11,12,43,44,45,46,47] as baits allowed us to identify 90 putative genes from GAs, JA, and Aux signaling. The genes from each family, as well as their locus IDs and physicochemical parameters of the codified proteins, are summarized in Tables S1–S3. To enhance readability and intuitiveness, the results are presented below in groups based on receptors, inhibitory proteins, and transcription factors.

Phylogenetic analyses using full-length protein sequences were employed to assess and establish evolutionary relationships among the genes analyzed (Figure 2, Figure 3 and Figure 4 and Figures S1–S10 and Tables S4–S6). Considering the availability of signaling genes identified among plant species, each phylogenetic tree included different species groups; however, Arabidopsis, rice (*Oryza sativa*), and the castor bean were always included.

**Figure 2:**
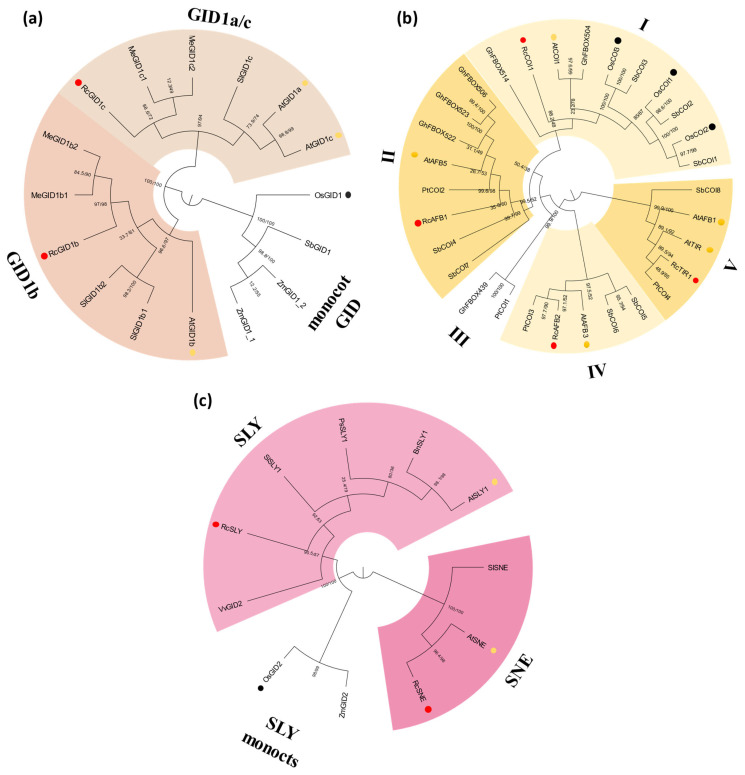
Maximum-likelihood phylogenetic analysis of receptors and F-box of jasmonate, gibberellin, and auxin signaling. Protein sequences of GID1 (**a**), COI and TIR (**b**), and SLY (**c**) were aligned using MUSCLE in MEGA 11, and phylogenetic reconstructions were made using the maximum likelihood method under the best model selection in iQtree software (version 1.6.12) with 1000 replicates of bootstrap and Approximate likelihood-ratio test (ALRt) statistics. Protein sequences from *R. communis*, *A. thaliana*, and *O. sativa* are highlighted by circles red, yellow, and black respectively. Different species were used for phylogenetic analysis: *Solanum lycopersicum* (Sl), *Manihot esculenta* (Me), *Zea mays* (Zm), *Sorghum bicolor* (Sb), *Populus trichocarpa* (Pt), *Gossypium hirsutum* (Gh), *Vitis vinifera* (Vv), *Brassica napus* (Bn), and *Prunus salicina* (Ps). The colors used in the phylogenetic trees correlate with the color scheme for each protein used in Figure 1. The roman numerals in (**b**) indicate the different groups observed.

Similar to what was observed in other species [11], the castor bean contains two putative gibberellin receptors, RcGID1b and RcGID1c, distributed in the clades GIDb and GIDa/c, respectively (Figure 2a and Figure S1, Table S1). The jasmonate receptor COI1 is an F-box protein from the C3 subfamily [45]. This family also contains the Aux receptor TIR1. In arabidopsis, C3 subfamily contains four proteins: COI1, ABF5, TIR1 and ABF3. Consistent with that, we identified four castor bean F-box genes related to the C3 subfamily (Tables S2 and S3). Phylogenetic analysis indicates that these proteins can be separated into five subgroups. The subgroup I includes a group of proteins, along with the COI1 proteins identified in Arabidopsis, rice, and the castor bean RcCOI1 (Figure 2b and Figure S2). Subgroup II includes the castor bean AFB1, which is related to Arabidopsis ABF5 (Figure 2b and Figure S2). Castor bean display other sequences that group with Arabidopsis ABF3 in subgroup IV, and Arabidopsis TIR and AFB1 in subgroup V: RcAFB2 and RcTIR1, respectively (Figure 2b and Figure S2).

SLY/SNE F-box proteins give specificity to the SCF complex for gibberellin signaling [6]. Two putative *SLY/SNE* genes were identified in the castor bean genome (Table S1). Phylogenetic analysis follows the pattern observed in other eudicotyledonous plants, with two clades, one related to SLY and the other to SNE [48] (Figure 2c and Figure S3).

**Figure 3:**
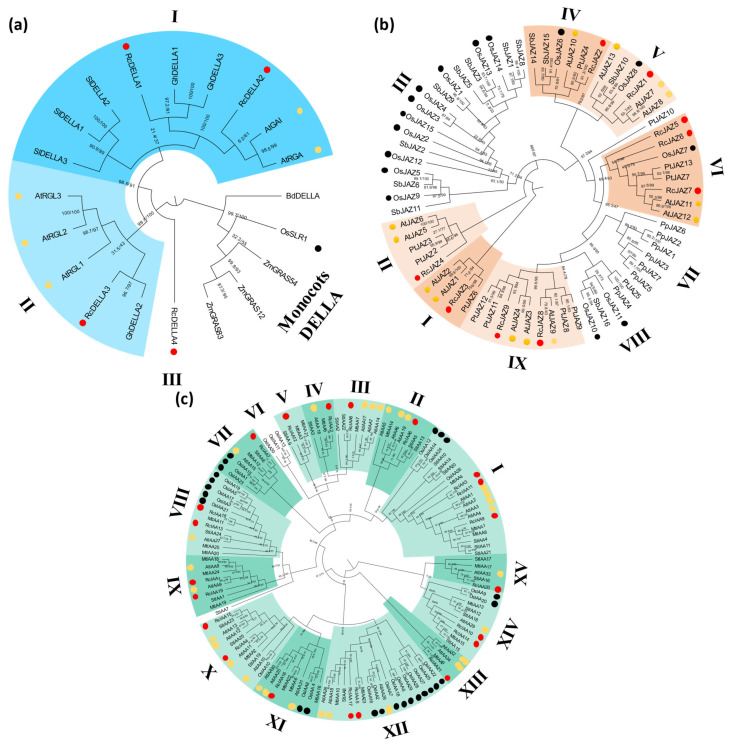
Maximum-likelihood phylogenetic analysis of repressor proteins of jasmonate, gibberellin, and auxin signaling. Protein sequences of DELLA (**a**), JAZ (**b**), and IAA (**c**) were aligned using MUSCLE in MEGA 11, and phylogenetic reconstructions were made using the maximum likelihood method under the best model selection in iQtree software (version 1.6.12) with 1000 replicates of bootstrap and Approximate likelihood-ratio test (ALRt) statistics. Protein sequences from *R. communis*, *A. thaliana*, and *O. sativa* are highlighted by circles red, yellow, and black respectively. Different species were used for phylogenetic analysis: *Solanum lycopersicum* (Sl), *Zea mays* (Zm), *Sorghum bicolor* (Sb), *Brachypodium distachyon* (Bd), *Gossypium hirsutum* (Gh), *Physcomitrella parttens* (Pp), *Solanum tuberosum* (St), and *Medicago. truncatula* (Mt) and *Populus trichocarpa* (Pt). The colors used in the phylogenetic trees correlate with the color scheme for each protein used in Figure 1. The roman numerals in (**b**,**c**) indicate the different groups observed.

The search for the repressor from GA signaling identifies four putative *RcDELLA* genes in the castor bean genome. Phylogenetic analysis indicates that DELLA proteins from eudicot and monocots form two separate groups and that eudicot DELLAs can be subdivided into three subgroups, with RcDELLA1 and RcDELLA2 located in subgroup I, RcDELLA3 in subgroup II, and RcDELLA4 outside of subgroups (Figure 3a and Figure S4). Our analysis identified nine castor bean genes encoding JAZ, the inhibitory protein from JA signaling (Table S2). The phylogenetic analysis of JAZ proteins divides the sequences into nine groups (I to IX) (Figure 3b and Figure S5). Except for TIR1, genes from the Aux signaling have been previously analysed in the castor bean [43], and nineteen inhibitory proteins from the IAA family were identified. Our de novo search expands this characterization, identifying two additional *IAA* genes, *RcIAA20* and *RcIAA21* (Table S3). Phylogenetic analysis demonstrated that IAA proteins can be separated into sixteen groups (I-XVI). Except for groups VI and X, composed exclusively of rice and potato (*Solanum tuberosum*) sequences, respectively, all groups contain at least one castor bean sequence (Figure 3c and Figure S6).

**Figure 4:**
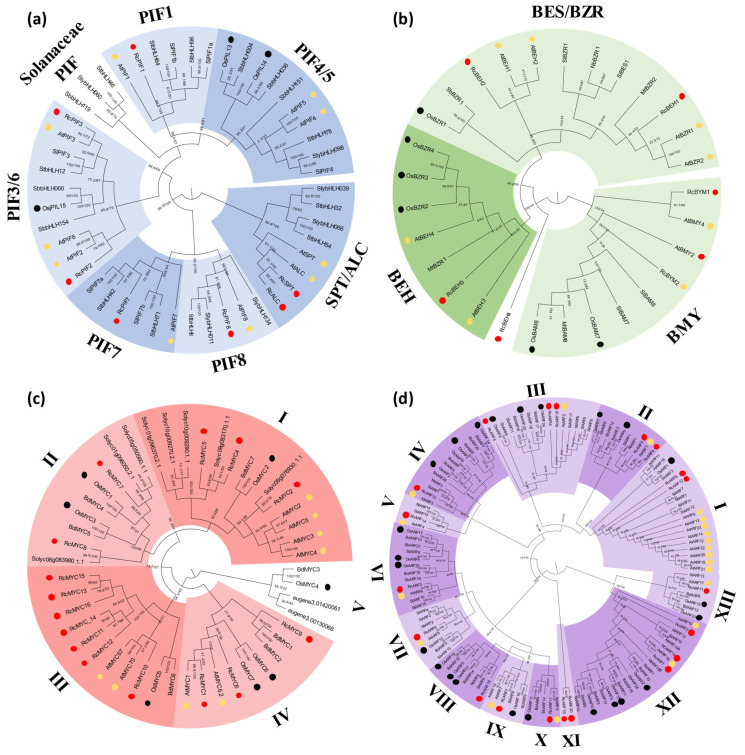
Maximum-likelihood phylogenetic analysis of transcription factors of jasmonate, gibberellin, and auxin signaling components. Protein sequences of PIF (**a**), BEH (**b**), MYC (**c**), and ARF (**d**) were aligned using MUSCLE in MEGA 11, and phylogenetic reconstructions were made using the maximum likelihood method under the best model selection in iQtree software (version 1.6.12) with 1000 replicates of bootstrap and approximate likelihood-ratio test (aLRT) statistics. Protein sequences from *R. communis* (Rc) are highlighted by circles in red, *A. thaliana* (At) in yellow, and *Oryza sativa* (Os) in black. *Solanum lycopersicum* (Sl), *Sorghum bicolor* (Sb), *Populus trichocarpa* (Pt), *Brachypodium distachyon* (Bd), *Solanum tuberosum* (St), *Medicago truncatula* (Mt), *Nicotiana tabacum* (Nt), and *Nicotiana benthamiana* (Nb). The colors used in the phylogenetic trees correlate with the color scheme for each protein used in Figure 1. The roman numerals in (**c**,**d**) indicate the different groups observed.

Considering abiotic stress, GA signaling operates with two main families of transcription factors: PIFs/ALC/SPT and BES1/BZR1/BEH [16]. In Arabidopsis, the PIF family can be grouped into five clades (PIF1, PIF3/6, PIF 4/5, PIF7, PIF8) [40]. Our analysis identifies five castor bean PIF genes: *RcPIF1*, *RcPIF2*, *RcPIF3*, *RcPIF7*, and *RcPIF8* (Figure 4a and Figure S7). Two other bHLH proteins, ALC and SPT, are closely related to PIF and strongly related to GA signaling [36]. Similar genes were also identified in the castor bean genome (*RcALC* and *RcSPT*, Figure 4a and Figure S7). The analysis of the BES/BZR/BEH transcription factors identifies four *BEH* genes in the castor bean genome (*RcBEH1* to 4, Figure 4b and Figure S8). We also identify two *BMY* genes, where the BES/BZR DNA binding domain is combined with a beta-amylase domain (BAM) [49]. Among the BEH genes identified, *RcBEH1* is the closest homologue to the Arabidopsis *BES1* and *BZR1* (Figure 4b and Figure S8). The search for *MYC* genes, recognized to encode the major transcription factor from the JA signaling, allows us to identify fifteen putative genes in the castor bean genome (*RcMYC1* to *RcMYC15*). Phylogenetic analysis groups the MYC proteins into five different groups. RcMYC2, RcMYC4, and RcMYC5 are present in group I, together with the main MYC proteins related to JA-induced responses from Arabidopsis (AtMYC2, AtMYC3, AtMYC4, and AtMYC5) [23] (Figure 4c and Figure S9). ARF proteins represent the main transcription factors involved in Aux signaling [26]. Different from the observed for the Aux/IAA family, our de novo analysis identifies the same eighteen ARF genes previously identified, [43]. Phylogenetic analyses grouped into thirteen clades (I–XIII). Among these, clade V shows only eudicot sequences, and clade XI is composed only of RcARF19 and RcARF20 (Figure 4d and Figure S10).

### 2.2. Chromosomal Positions, Synteny, Collinearity, and Duplication Analysis

**Figure 5:**
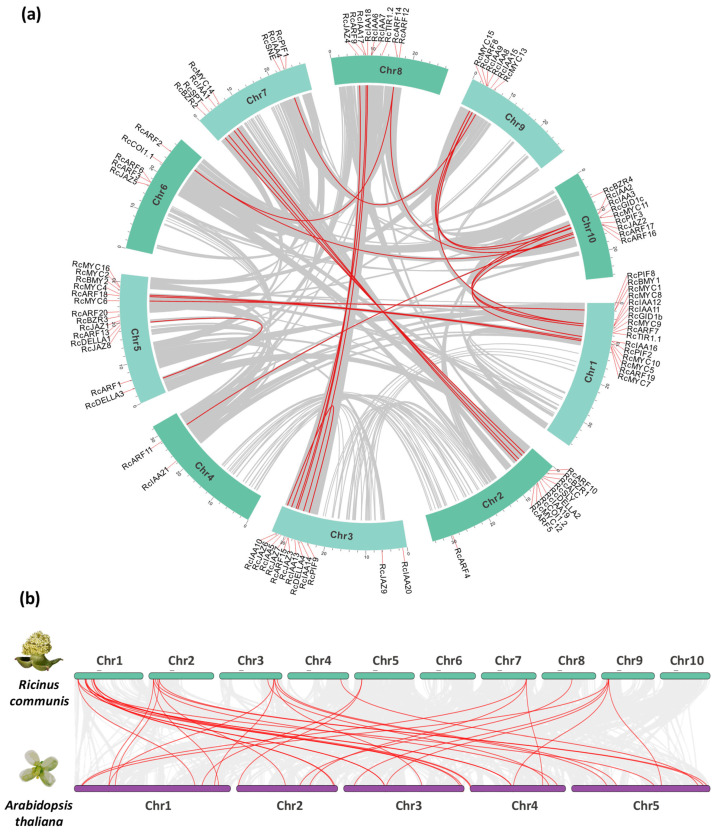
Chromosomal positions and inter-chromosomal groups of duplicated *GID*, *COI*, *AFB*, *TIR*, *SLY*, *SNE*, *DELLA*, *JAZ*, *IAA*, *PIF*, *BEH*, *MYC*, and *ARF* gene pairs in *Ricinus communis* (**a**) and synteny with the *Arabidopsis thaliana* (**b**). Localization of identified genes in castor bean chromosomes is indicated outside of the chromosome circle. Gray lines in the background demonstrate syntenic blocks, and the red lines exhibit the segmental or tandem duplication of identified genes.

To investigate the physical location and duplication events within each analyzed family (Tables S1–S3), the positions from each gene on castor bean chromosomes were located based on the data obtained from the castor bean genome database at NCBI (GCF_019578655.1) (Figure 5a). The collinearity between the castor bean and the Arabidopsis genomes was compared, and both orthologous and paralogous gene pairs are highlighted in red (Figure 5b) and shown in Tables S7 and S8.

Most of the duplications are concentrated in the IAA and ARF families, followed by JAZ and MYC. The expansion of Aux and JA signaling is closely linked to the evolution of land plants and the increasing complexity of this lineage over time. Interestingly, given the similarities between JA and Aux signaling, it is plausible that they share a common evolutionary origin [50]. Additionally, duplications can be identified between *RcGID1a* and *RcGID1c*, which originated in the eudicot ancestor [51], as well as between *RcDELLA1* and *RcDELLA3*. Independent phylogenetic analyses suggest that two major duplication events occurred in the evolutionary history of *DELLA* genes: the first in the ancestor of vascular plants (*DELLA1–DELLA2*) and the second in eudicot flowering plants (*DELLA1–DELLA3*) [52].

To analyze the relationship between genetic divergence and gene duplication events in paralogous genes, their Ka/Ks ratios were determined. The values < 1 found in the analysis indicate a purifying selection force for all duplicated genes (Table S7), which could mean that the duplicated genes did not accumulate differences to lead to loss or change of function.

### 2.3. Gene Structure and Distribution of Conserved Motifs

To analyze the structural features of castor bean GAs, JA, and Aux signaling genes, exon-intron structure and protein motif analysis were performed and compared with their Arabidopsis counterparts for receptors (Figure S11), inhibitory proteins (Figure S12), and transcription factors (Figures S13 and S14). For GID1, TIR, COI, and SLY gene families, similar exon-intron structures (Figure S11a,c,e) and protein motifs (Figure S11b,d,f) were observed.

GID1 features a N-terminal extension that is highly conserved across GID1 members (motifs 4, 6, and 8; Figure S11b). The C-terminal core domain of GID1 shares significant similarity with the plant carboxylesterase family within the a/b-hydrolase fold superfamily (motifs 8, 9, 2, 5, 10, 1, 7, 3, and 11; Figure S11b). This C-terminal core domain forms a GA-binding pocket, while the N-terminal extension acts as a lid for the pocket [12,53]. The motifs of RcGID1c and RcGID1b show strong similarities to those of Arabidopsis GID1s (Figure S11b). Interestingly, AtGID1b exhibits a unique property: it can bind to DELLAs in a GA-independent manner. Additionally, AtGID1b is hypersensitive to GA [12,53]. GA receptor type “b” is also present in soybean (*Glycine max*), displaying the same characteristics observed in Arabidopsis, which seems to have emerged independently through eudicots [11].

COI1, TIR1, and AFB proteins all contain an F-box N-terminal domain (motif 1; Figure S11d) and LRR domains located in the middle of the protein (comprising the motifs: 9, 4, 11, 12, 15, 13, 5, 3, 8, and 2). The surface pocket within the LRR domain can be divided into four distinct pockets, each involving different residues and exhibiting unique surface properties crucial for JA binding [20]. TIR1 shares significant similarity with COI1; however, recent studies have identified an adenylate-cyclase domain in the C-terminal region [54]. This domain, located in motif 7, is specifically found in TIR1 and AFB proteins (Figure S11d). All these motifs are present in castor bean orthologues. Structural studies of TIR1 have revealed that a single pocket formed by the LRR domain binds Aux and the inhibitory protein IAA. Aux is located at the base of this pocket and helps stabilize the binding of IAA to TIR1 [55].

SLY1 and SNE contain three domains: the F-box domain (motif 1), the N-terminal, and the C-terminal regions (Figure S11f). The F-box domain is essential for the formation of the SCF complex [55]. Previous yeast two-hybrid studies have shown that the C-terminal domain of SLY1 is crucial for interaction with DELLA proteins, and this region exhibits the highest sequence homology between SLY1 and SNE (motif 3; Figure S11f) [13].

*DELLA* genes are predominantly intronless, except for *RcDELLA1* (Figure S12a). Among the RcDELLA protein sequences, RcDELLA1 and RcDELLA3 exhibit more conserved motifs (Figure S12b). DELLA, like all GRAS family members, contains a C-terminal GRAS domain that confers transcriptional regulator function [52], which has been shown to be conserved between Arabidopsis and castor bean (Figure S12b). The N-terminal region of DELLA features a unique DELLA domain essential for GA-induced degradation, which is also conserved in RcDELLA1 and RcDELLA3 from castor bean (motifs 14, 7, and 9). However, this domain does not interact with the F-box protein [53]. It has been shown that GID1 directly binds to the conserved DELLA, LEXLE, and VHYNP motifs within the N-terminal domain of DELLA proteins, in a GA-dependent manner [53].

*RcJAZ* genes display variable exon-intron structures (Figure S12c). However, the same variation in the structures and length occurs in Arabidopsis. Besides the differences observed in exon-intron structures, the protein motif distribution is similar among the different subclades (Figure S12c,d). The C-terminal region, which contains the JA-associated (Jas) domain, is both necessary and sufficient for interaction with COI1 and JA binding to the complex [56] (motif 1; Figure S12d). The α-helix of the JAZ degron binds COI1 near the JA-Ile binding site, and this interaction is essential for co-receptor function [56]. Motifs 2 and 3 correspond to the ZIM domain (Figure S12d). The conserved N-terminal region shared by RcJAZ3, RcJAZ4, and AtJAZ1 contains the motif required for JAZ-DELLA interaction (motif 4; Figure S12d).

In the *IAA* genes, the intron number ranged from one to six, and the length was less variable (Figure S12e). The exceptions are *RcIAA10*, which is longer and has more introns, and *RcIAA15,* which is shorter and intronless. These structures are not observed in the Arabidopsis IAA counterparts. The protein motifs vary between IAA types; however, motif 1, known as domain III/IV, is conserved among different types of IAA, and it is important to bind AFRs through shared domains [57]. Except for RcIAA21, this motif is present in all castor bean IAA sequences (Figure S12f). The N-terminal domain I (motif 4) is required for transcriptional repression and recruits co-repressor proteins, including TOPLESS (TPL). Domain II (motifs 2 and 3) is responsible for protein instability through direct interaction with auxin and the TIR1/AFBs. This domain contains a 13 amino acid degron motif, which directly contacts TIR1 and Aux [57].

When compared with Arabidopsis, the structure of the PIF, BEH/BMY, and MYC gene families seems to be more conserved. DELLA proteins were shown to directly interact with the DNA-binding domain of bHLH transcription factors [58].

The key motifs of PIF proteins are conserved between castor bean and Arabidopsis, underscoring their functional importance. Motif 1, which contains the bHLH domain, is critical for DNA binding, dimerization, and interaction with DELLA [40]. Motif 2, related to the APB domain, facilitates interactions with phyB, while motif 15, associated with the APA domain and present exclusively in PIF1 and PIF3, is essential for interacting with phyA (Figure S13a,b) [59].

BEH proteins display a conserved DNA-binding domain at the N-terminal region (motif 1), while the C-terminal ends differ among the subfamilies (Figure S13d). Motifs 2, 7, and 10 are conserved between Arabidopsis and castor bean BEHs, and this region is known to contain phosphorylation sites [60]. Interestingly, this region is not found in BMY proteins, which display a beta-amylase domain (Figure S13d).

MYC proteins show a conserved bHLH domain at the C-terminus (motif 2). The basic domain of bHLH is mainly responsible for binding the G-box, and the HLH is a loop region responsible for the formation of homo or heterodimers. The N-terminal region interacts with DELLA [61] and may contain the JID domain, which is responsible for interacting with the JAZ protein. The JID domain is conserved only in AtMYC2, AtMYC3, AtMYC4, and RcMYC2 (motifs 15, 6, and 8; Figure S13f).

ARF proteins are highly conserved. Similar to IAAs, ARFs also consist of modular domains (domains III/IV) in the C-terminal region (motifs 14 and 9). ARFs contain an N-terminal DNA-binding domain (motifs 1 and 3) and a middle domain that acts either as an activator or a repressor [62].

### 2.4. Cis-Regulatory Elements and microRNAs Predicted to Target the Identified Genes

**Figure 6:**
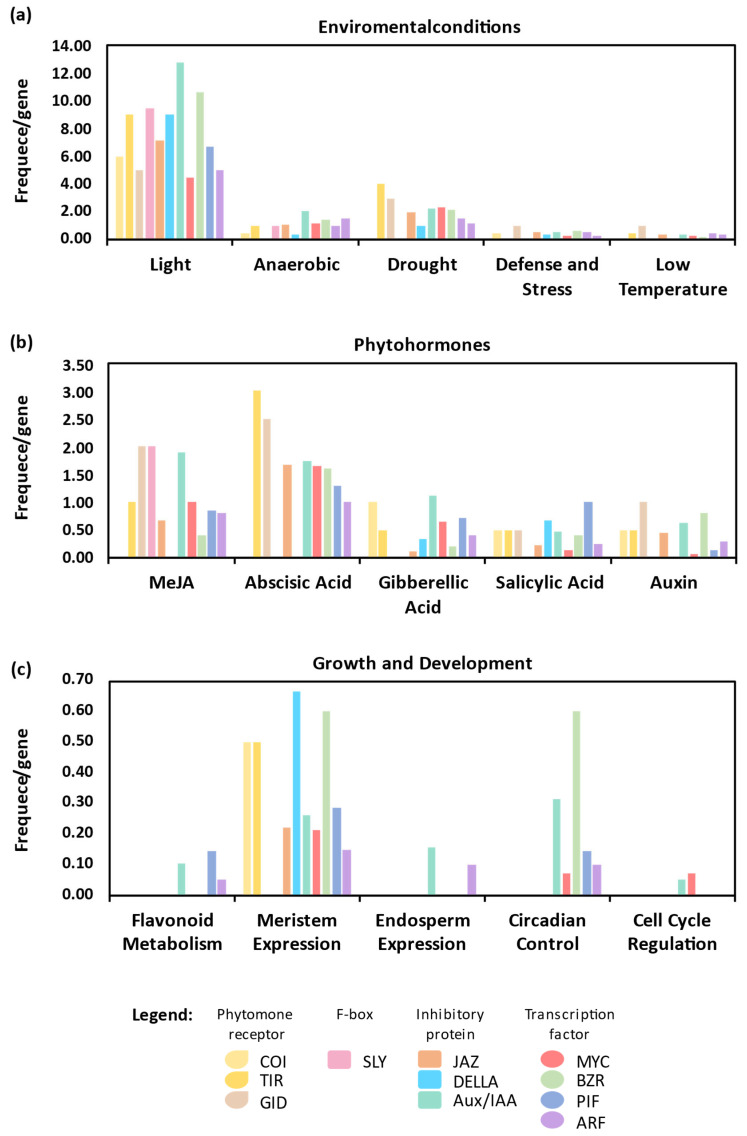
*Cis*-regulatory elements in the *Ricinus communis* COI1/TIR1, GID1, JAZ, DELLA, IAA, MYC, PIF, ARF, BZR, and SLY promoter regions in environmental conditions (**a**), phytohormones (**b**), and growth and development (**c**). The total number of *cis-*regulatory elements per number of genes involved in abiotic stress, phytohormones, and growth and development, respectively. Colors signify different gene families as indicated.

The analysis of *cis*-regulatory elements in promoter regions is a useful strategy to explore the regulation of the selected genes. All *cis*-regulatory elements are summarized in Figure 6.

Among environmental condition-related *cis*-elements (Figure 6a), those associated with drought and dehydration responses (such as MBS, ABRE, DRE, and MYC) are predominantly found in *TIR* and *GID* genes. Regarding phytohormones, the *TIR* and *GID* receptor families contain a higher number of ABA-related *cis*-elements, with the ABA-responsive element (ARE) being the most prevalent (Figure 6b). Except for *COI1*, *SLY*, and *DELLA*, ABA-related elements are abundant across all gene families examined. Additionally, *cis*-elements responsive to JA, GA, salicylic acid, and Aux are frequently present, suggesting regulation by multiple phytohormones (Figure 6b). In the context of growth and development, meristem-associated *cis*-elements are particularly enriched (Figure 6c), aligning with the well-established roles of these signaling in shoot apical meristem regulation across plant species [63,64].

MicroRNAs (miRNAs) are known to fine-tune the expression of numerous plant genes [65]. To evaluate if the castor bean genes from GA, JA, and Aux signaling identified could be targeted by miRNA regulation, we used an in silico approach. Among the genes from phytohormone signaling analyzed, Aux signaling displays the highest number of genes with miRNA targets (25 genes), followed by JA signaling with 14 genes and GA signaling with 6 genes (Tables S9–S11). These genes were predicted as targets for 17 miRNA family genes in the Aux signaling, 18 in the GA, and 11 in the JA. Some of the miRNAs identified here have been previously demonstrated to regulate hormonal signaling genes in other species. miR393, miR156 and miR169 are involved in the control of *TIR1*, *MYC* and *JAZ*-related homologues, respectively. The miR168 and miR395 families are able to regulate genes from all three hormone signaling pathways analyzed (Tables S9–S11). The identification of *cis*-elements and potential miRNA targets can indicate a possible scenario of the regulation of the genes from GA, JA, and Aux signaling identified in the castor bean. Future experimental validations are necessary to evaluate their potential impact on the regulation of the expression of these genes.

### 2.5. Expression Profiles of Castor Bean GA, JA, and Aux Signaling Genes Under Drought Stress

**Figure 7:**
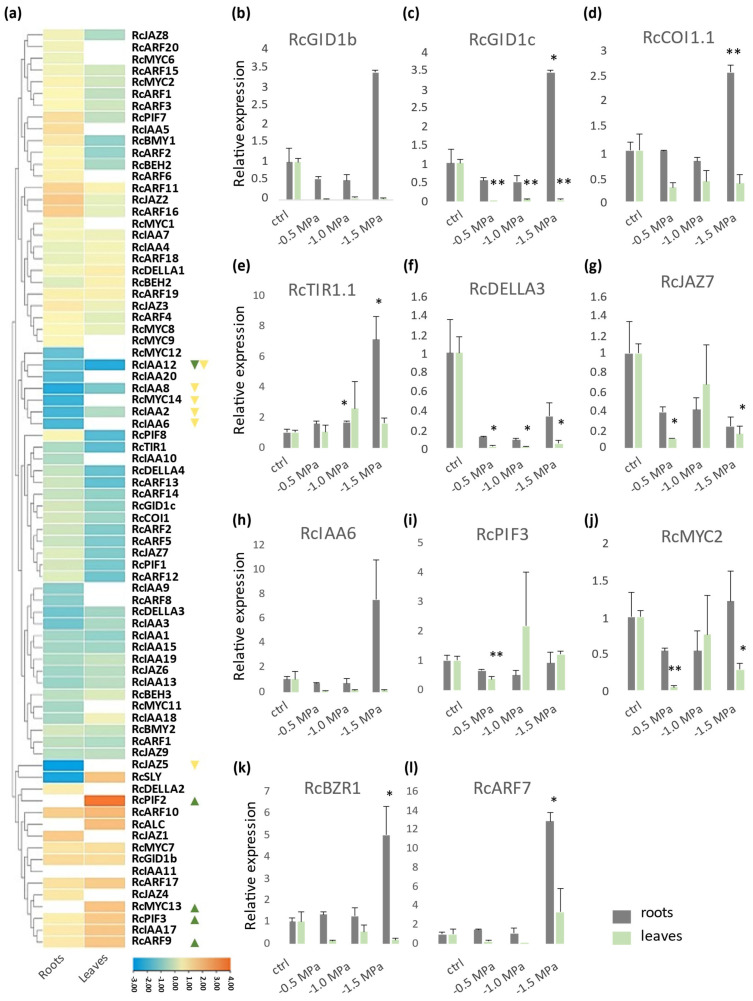
Expression profile under drought stress. (**a**) Heatmap showing the expression pattern of identified genes in leaves and roots under drought stress (−1.0 MPa). Blank spaces mean non-identified genes in the expression data. Triangles indicate statistical significance (up to 1.8-fold change) to upregulated and downregulated genes in leaves (green) and roots (yellow). Relative expression of (**b**) *RcGID1b*, (**c**) *RcGID1b*, (**d**) *RcCOI1*, (**e**) *RcTIR1*, (**f**) *RcDELLA3*, (**g**) *RcJAZ7*, (**h**) *RcIAA6*, (**i**) *RcPIF3*, (**j**) *RcMYC2*, (**k**) *RcBEH1*, and (**l**) *RcARF7* under mild (−0.5 MPa), moderate (−1.0 MPa) and severe (−1.5 MPa) drought stress. Grey bars represent roots and green bars represent leaves. Asterisk indicates statistical difference compared to control, * >0.05, ** >0.01.

To initiate the characterization of the expression profile of the genes from GA, JA, and Aux signaling in castor bean under drought conditions, RNA-seq data obtained from plants submitted to drought stress at water potential of −1.0 MPa (Bioproject: PRJNA401329) [8] were analyzed. The expression analysis of 90 genes related to GA, JA, and Aux signaling identified three larger clusters of gene expression profiles: one with genes upregulated, one with downregulated genes, and the third one with low-modulated genes (Figure 7a). It is possible to observe that the modulation in leaves and roots is similar.

To expand our understanding of GA, JA, and Aux signaling in the drought response, we performed RT-qPCR analyses on genes that are either fundamental to these pathways or potentially involved in drought adaptation. This analysis allowed us to quantify the expression levels of key signaling components, including receptors, inhibitory proteins, and transcription factors, under different stress intensities: mild (−0.5 MPa), moderate (−1.0 MPa), and severe (−1.5 MPa).

The receptors *RcGID1c*, *RcCOI1.1*, and *RcTIR1.1* were mainly upregulated in roots under severe stress, increasing by approximately 238.2%, 154.0%, and 611.7%, respectively. In contrast, their modulation in leaves was generally non-significant, except for *RcGID1c*, which was strongly repressed at all stress levels: 99.2% under mild, 96.0% under moderate, and 96.7% under severe conditions (Figure 7c–e).

Among inhibitory proteins, *RcDELLA3*, *RcJAZ7*, and *RcIAA6* were modulated in leaves, starting at −0.5 MPa. *RcDELLA3* was downregulated in both organs, with significant repression in leaves at all stress levels (98.5% under mild, 99.4% under moderate, and 97.3% under severe stress) and a clear tendency toward repression in roots. *RcJAZ7* expression in leaves was abolished under mild stress and downregulated by 87.5% under severe stress. *RcIAA6* expression tends to decrease in leaves and increase in roots under severe conditions, although these changes were not statistically significant (Figure 7f–h).

Regarding transcription factors, the *RcPIF3* expression remained unchanged except for a 64.7% repression in leaves under mild stress. *RcMYC2* was modulated in leaves under mild and severe stress, showing reductions of 64.7% and 63.7%, respectively. In contrast, *RcBEH1* and *RcARF7* were strongly induced in roots under severe stress, increasing by 399.2% and 1188.1%, respectively (Figure 7i–l).

## 3. Discussion

The phytohormones coordinate stress adaptation by balancing growth and survival through intricate crosstalk mechanisms. The ability to regulate responses through diverse hormonal interactions has been key to plant adaptation in fluctuating environments during its evolutionary life story. This trade-off reflects a strategic allocation of energy resources, ensuring survival without irreversibly compromising development. As climate change intensifies and plant resource demands rise, understanding this signaling presents new biotechnological opportunities for improving drought resilience. Castor bean’s remarkable drought tolerance, coupled with its economic potential for seed oil production, highlights the need for a comprehensive understanding of phytohormone signaling and stress response.

Despite castor bean’s great adaptation to limiting water conditions, the molecular characterization of this response is still limited. Previous transcriptomic analysis from our group has started the evaluation of the castor bean’s general response to drought stress [8], ROS scavenger genes [66,67], and the central core of ABA signaling [8]. The present work aims to continue the identification of genes and genetic circuits that may enhance our understanding of the molecular mechanisms employed by the castor bean in response to water stress.

Here, we perform a systematic analysis of the components of GA, JA, and Aux signaling, as well as a transcriptomic analysis of their response upon drought treatments in leaves and roots. This study enhances our understanding of the phylogenetic, syntenic, structural, and transcriptomic aspects of GA, JA, and Aux-related genes in castor bean and their expression under drought stress. These insights may contribute to developing innovative biotechnological strategies to enhance crop adaptation to climate change, ensuring agricultural sustainability in unfavorable conditions.

### 3.1. Gibberellin Signaling

Our analysis identified two GID1 proteins in castor bean (RcGID1b and RcGID1c). This identification is in accordance with previous work, which demonstrates that the GID1 family expands and diversifies after the emergence of angiosperms, into two subfamilies, GID1ac and GID1b [11]. In the Brassicaceae family, the GID1ac subfamily expanded with the emergence of a well-defined clade containing proteins related to Arabidopsis GID1a [9]. Thus, Arabidopsis possesses both GID1a and GID1c, whereas castor bean retain only a single GID1c, which is outside the GID1a clade. Sequences of RcGID1b and RcGID1c are highly conserved compared to the extensively studied GID proteins from Arabidopsis, as well as other eudicot plants. This conservation strongly suggests that RcGID1b and RcGID1c are the functional gibberellin receptors. Phylogenetic and structural analysis demonstrated that GID1 belongs to the carboxylesterase family (CXE) and that the GA perception complex (GA-GID1) is functional only in tracheophytes [51]. Rice plants with mutations in *GID1* display an increase in the endogenous GA levels and higher stomatal density. Since these plants show normal ABA levels, the stomatal density alteration seems to be primarily influenced by the disruption in GA signaling. On the other hand, in tomato (*Solanum lycopersicum*), the loss of GID1a reduces water loss and causes whole plant transpiration under drought stress without affecting plant growth. This can be explained by the redundancy between GID1 paralogues in eudicots, allowing a mild attenuation of GA signalling when *GID1a* is mutated [68]. The ortholog of *SlGID1a* in castor bean, *RcGID1c* (Figure 1 and Figure S2a), undergoes substantial repression in leaf tissues, starting from water stress −0.5 MPa (Figure 7b). Since, in similar situations, the expression of *RcGID1b* is not repressed (Figure 7a), the attenuated expression of *RcGID1c* in leaves may contribute to the drought tolerance phenotype observed in castor bean.

The significance of the turnoff of GA signaling for drought tolerance was further reinforced through the manipulation of DELLA inhibitory proteins. In tomato, a gain-of-function mutation in the *SlDELLA3/PROCERA* gene (*proceraΔ17/proΔ17*) results in plants with decreased transpiration and enhanced tolerance to drought [69]. Similar results are observed in Arabidopsis, where the gain-of-function mutant in *GAI1* (*GA-insensitive 1, gai-1*) also increases drought tolerance [70]. DELLA serves as the primary suppressor of GA hormone responses in vascular plants and acts as a central hub interactor, giving rise to diverse responses based on the specific protein with which it interacts [52]. Osmotic or drought stress has been demonstrated to decrease GA content, thereby increasing DELLA protein accumulation in plants. This, in turn, further enhances the stress tolerance phenotype in growth-retarded plants [4]. DELLA proteins also play a key role in regulating hypocotyl elongation by interacting with multiple transcription factors. In Arabidopsis, they repress BZR1, ARF6, PIF3, and form the BZR-ARF-PIF/DELLA (BAP/D) complex, which mediates the coordinated regulation of cell elongation during seedling photomorphogenesis [15]. Beyond promoting developmental processes such as cell elongation and division, DELLA proteins also modulate GA crosstalk with other phytohormone signals, leading to the activation of genes essential for stress adaptation, including drought tolerance [12,13]. Our de novo identification of *DELLA* genes confirmed the previous identification of four DELLA proteins in castor bean [69]. The phylogenetic and protein motifs analyses show that RcDELLA1 and RcDELLA2 are closely related to Arabidopsis GAI/RGA and tomato PROCERA, which, when carrying gain-of-function mutations, enhance drought tolerance [70]. This phylogeny is consistent with the hypothesis that in early diverging land plants, a single *DELLA* gene was present, which underwent two duplication events. One is the ancestor of vascular plants, and another is the ancestor of eudicots, resulting in at least three *DELLA* paralogues [70]. However, due to the dynamic history of genome loss and gain throughout evolution, the number of *DELLA* paralogues varies across species [70]. *RcDELLA1* and *RcDELLA2* demonstrate a tendency towards upregulation in response to drought, although without statistical significance (Figure 7a). *RcDELLA3* exhibits significant repression upon water stress imposition (Figure 7f), and *RcDELLA4* is clustered among the downregulated genes (Figure 7a). The inhibition of *RcDELLA3* and the lack of significant changes in other *DELLA* genes indicate a nuanced regulation of *DELLA* transcription in response to drought. This is further demonstrated by the expression analysis of *RcGID1a* and *RcGID1b*, reinforcing the idea that the mild suppression of GA signaling may play a crucial role in the drought tolerance phenotype [71].

PIF orchestrates plant responses to a wide range of stresses, including temperature, shade, drought, and other abiotic and biotic challenges, by interacting with multiple hormonal signals, such as GAs and Aux [72]. Recently, the potential of PIFs to improve tolerance to adverse environmental conditions has been highlighted [73]. *AtPIF3* and its orthologues in maize, *ZmPIF1* and *ZmPIF3,* reduce transpiration in response to drought and improve the tolerance [72]. The overexpression of carrot (*Daucus carota*) *DcPIF3* in Arabidopsis enhanced tolerance to drought stress by increasing endogenous ABA levels [74]. In castor bean, RNA-seq and RT-qPCR analyses indicate that *RcPIF3* was upregulated in leaves at −1.0 MPa (Figure 7a). However, the RT-qPCR statistical analysis indicates repression at −0.5 MPa (Figure 7i). In contrast, rice plants overexpressing *OsPIL13*, the orthologue of *AtPIF4*, show increased drought-induced damage [75]. Notably, castor bean lack an orthologue of this gene (Figure 4a and Figure S6). Similarly, overexpression of PIF1 from tobacco (*Nicotiana tabacum*) increases drought sensitivity by repressing carotenoid and ABA biosynthesis, while the null mutant enhances drought tolerance [76]. Consistent with this, *PIF1* from castor bean was found to be downregulated in leaves (Figure 7a).

Although the BES/BZR transcription factors are mainly associated with BR signaling, they are also involved in drought response [77]. In Arabidopsis, BES1 represses the expression of *RD26* (*RESPONSIVE TO DESICCATION 26*). RD26 also interacts with BES1 to inhibit their transcriptional activity. This inhibitory mechanism not only ensures that BR-induced growth is inhibited under drought stress but also prevents unnecessary activation of drought response when plants undergo BR-induced growth [78]. In wheat (*Triticum aestivum*), *TaBRZ2* is induced under drought conditions, and its overexpression enhances drought tolerance, whereas silenced lines exhibit the opposite trend [77]. The *RcBEH1*, orthologue of the Arabidopsis *BES1*/*BZR1* (Figure 4b and Figure S7), was repressed in leaves under mild stress (−0.5 MPa) and strongly induced in roots under severe stress (−1.5 MPa) (Figure 7k). RNA-seq demonstrated that in moderate stress (−1.0 MPa), the *BEH* genes have low modulation, being preferentially repressed (Figure 7a). The modulation of BES/BZR transcription factors in drought response in most species is predominantly repressive. However, the Arabidopsis *bzr1-D* gain-of-function mutant exhibits greater resilience upon rewatering [79]. Altogether, these data suggest that *RcBEH1* high expression in roots under severe drought stress may lead to a drought tolerance phenotype.

### 3.2. Jasmonate Signaling

The activation of JA signaling was also elucidated as important to drought response. The JA receptor COI1 is an F-box protein of group C3 [45]. TIR/AFB and COI1, which are involved respectively in Aux and JA signaling, and are derived from the same gene during the transition from charophyte to embryophyte; however, the dedicated function as receptors of those hormones was established only in land plants [50]. Consistent with the pattern observed in other species [50], only one *COI1* gene was found in the castor bean genome. Mutants in *COI1* have increased drought sensitivity, indicating its importance in drought response [80]. How COI1 participates in drought signaling is not clear. However, the role of COI1 in acetate-mediated drought tolerance was demonstrated to be dependent on Histone Deacetylase 6 (HDA6) global deacetylation activity, leading to the enrichment of histone 4 (H4) acetylation, which influences the priming of the JA signaling for plant drought tolerance [81]. The *RcCOI1* upregulation in roots under severe drought stress (−1.5 MPa) indicates that COI1-dependent JA signaling may be involved in the castor bean late-stage stress adaptation (Figure 7c).

The appearance of the core of JA signaling has been dated to the emergence of land plants [80]; however, since liverwort shows only one JAZ gene, the JAZ expansion seems to have occurred on the emergence of embryophytes [46]. This expansion may be related to the colonization success of land environments. JAZ proteins bind to transcription factors, such as MYC2, and limit their activity under normal conditions. During water stress, JAZ is degraded, releasing the transcription factors from the inhibition, enhancing drought tolerance by JA signaling [82]. In rice, the *jaz1* T-DNA insertion mutant shows increased drought tolerance [83]. However, overexpression of *AtJAZ7* also confers drought tolerance, indicating that different isoforms of JAZ proteins could act positively or negatively on the drought tolerance phenotype [84]. On the other hand, AtJAZ11/12 were related to the negative control of JA-induced inhibition of root growth [84]. Interestingly, the expression data show that *RcJAZ1* (orthologue of *AtJAZ7)* was upregulated in roots (Figure 2c, Figure S3 and Figure 7a), while *RcJAZ7* (orthologue of *AtJAZ11/12*) was downregulated in leaves under drought stress (Figure 2c, Figure S3 and Figure 7g). These results suggest possible roles of *RcJAZ1* and *RcJAZ7* in the castor bean drought response.

MYC is a subgroup of the bHLH superfamily of transcription factors. Among them, MYC2 is the main target of JAZ [44] and is also important to the establishment of a crosstalk of JA signaling with other hormones, such as ABA [85]. In Arabidopsis, the overexpression of *MYC2* increases ABA sensitivity, which is reduced in the *myc2* mutant [85]. The role of MYC2 as a positive regulator of ABA signaling is related to the expression of ABA-responsive gene *RD22* (*Responsive to desiccation 22*) [86]. Recently, increased drought tolerance in the *myc2* mutant was reported [61]. Interestingly, our data show a repression of *RcMYC2* in leaves under drought stress conditions (Figure 7a,j), indicating that a reduction in *RcMYC2* can be related to the castor bean drought response.

### 3.3. Auxin Signaling

The signaling mediated by Aux, which is traditionally related to development, was recently associated with abiotic stress responses [28,87]. The Aux receptor TIR/AFB has the same evolutionary origin as the JA receptor COI [50]. However, there is an additional motif in the C-terminal (motif 7) region of *TIR/AFB* genes that corresponds to an adenylate cyclase (AC) activity site (Figure S11f), which was shown to be important for Aux response in Arabidopsis [54]. Different studies, including gene overexpression and transcriptome analysis, have demonstrated that many *TIR1/AFB* genes are responsive to drought [88]. In Arabidopsis, the loss of TIR1/AFB proteins increases drought tolerance [89]. However, our results show that *RcTIR1.1*, the orthologue of Arabidopsis *TIR1* (Figure 2a and Figure S1), was upregulated in roots under drought (−1.0 and −1.5 MPa) (Figure 7e), suggesting that castor bean and Arabidopsis may regulate Aux signaling under drought stress in different ways.

Previous studies also demonstrated the role of inhibitory protein IAA in Aux-mediated drought response. The expansion of this family with the presence of three functional domains (domains I, II, and III/IV) is also related to the embryophyte emergence, suggesting that, in some way, it was important for land colonization [50]. In Arabidopsis, IAA5/6/19 interacts with DREB (dehydration-responsive element binding) and is required for drought tolerance [90]. In addition, IAA inhibitory proteins are homologues to JAZ, reinforcing the idea that the JA and Aux signaling components have the same evolutionary origin [50]. Among the castor bean IAAs, RcIAA6 belongs to clade II, together with AtIAA5/6/19 (Figure 3c and Figure S5). *RcIAA6* was also modulated under drought stress, being downregulated in roots in 1.0 MPa (Figure 7a) but displaying a tendency to increase in roots under late point stress (Figure 7h).

ARFs are the main transcription factors that mediate the Aux response. They are inhibited by IAA proteins and also display crosstalk with GA signaling. Rice *OsARF16* is induced by water stress [87]. Phylogenetic analysis of ARFs (Figure 4d and Figure S9) reveals that *OsARF16* is an orthologue of *AtARF7*, which interacts with DELLA protein RGA [90]. Both genes are orthologues to *RcARF7*, which are also shown to be positively modulated during severe drought conditions in castor bean (Figure 7l). *RcARF2, RcARF3,* and *RcARF9* genes are orthologues of arabidopsis *AtARF10*, *AtARF6* and *AtARF8*, respectively (Figure 4d and Figure S9). In Arabidopsis plants exposed to water stress, miRNA160 represses *AtARF10,* and the overexpression of this microRNA confers ABA insensitivity [90]. In castor bean, the *AtARF10* orthologue *RcARF2* is also downregulated by drought, which may contribute to enhanced drought tolerance (Figure 7a). *AtARF6* and *AtARF8* are targets of the miRNA167, which is induced by ABA in response to water stress [65,91], indicating that, in Arabidopsis, the repression of these transcription factors may be related to drought response. However, the response of the castor bean orthologues seems to be different. *RcARF9* is upregulated in leaves under mild stress, and *RcARF3* does not display significant modulation to drought (Figure 7a).

### 3.4. Potential Regulation of Drought-Responsive Genes by miRNAs

The role of miRNAs in drought responses is increasingly evident, since they regulate key genes involved in hormonal and stress-related signaling [65]. The miR167, which is downregulated in response to drought in Arabidopsis, plays a crucial role in the ABA-independent drought response [92]. This miRNA is known to target Arabidopsis *ARF6* and *ARF8* [92] and may also target and regulate *RcARF9* and *RcIAA4* (Table S11). Additionally, miR167 may target *RcDELLA1* and *RcGID1b* (Table S9). This miRNA is associated with the regulation of transcription factors, including bHLH, and plays a role in modulating antioxidant defense genes in response to ABA [92], and its overexpression enhances drought tolerance in alfalfa [93]. In castor bean, miR156 may target *RcSNE* (Table S9), an F-box protein involved in SCF complex specificity for GID and DELLA binding, and may also regulate *RcMYC9* and *RcMYC14*. The miR156 is also predicted to target *RcIAA16* and *RcARF6*. The miR160, known for its role in Aux signaling, targets *ARF10*, *ARF16*, and *ARF17* in Arabidopsis and is linked to ABA-independent drought responses [92]. In castor bean, it may target and regulate *RcARF1*, *RcARF2*, *RcARF11*, and *RcARF13*, indicating that Aux signaling in castor bean could also be modulated by miRNAs during drought.

The miR159a, miR169, and miR393b also show potential involvement in drought responses [92,94]. The miR159a is predicted to target *RcCOI1.1*, while miR169 may control RcJAZ8, and miR393b may target *RcCOI1.2* and *RcTIR* genes (Table S10). These data suggest that the miR393-TIR1 module, known for its regulatory role in drought responses [92], could be conserved in the castor bean. This conservation highlights its functional relevance across species. Altogether, these miRNAs orchestrate complex regulatory networks by modulating key transcription factors, hormone signaling components, and stress-responsive genes [95]. Their interactions with target genes emphasize their essential role in drought adaptation, both in ABA-dependent and independent signals.

## 4. Conclusions

In the present work, the gene families involved in GA, JA, and Aux signaling in *Ricinus communis* were identified and annotated. Expression analysis of these genes under drought provided insights into how these phytohormones signaling are regulated during water stress, revealing potential regulatory networks that could explain the plant’s response to drought. This research highlights the nuanced regulation of GA signaling during drought, and the downregulation of the *RcMYC2*, a transcription factor involved in JA signaling, may contribute to drought tolerance. Aux signaling also exhibits distinct regulatory patterns, suggesting species-specific adaptations compared to Arabidopsis. Additionally, the miRNA prediction analysis suggests that miR167, miR160, and miR156 may regulate the expression of genes involved in GA, JA, and Aux signaling.

Drought is a major challenge to global food production, causing significant yield losses annually, further exacerbated by climate change and decreasing freshwater resources. Given the critical role of hormone signaling in plant stress responses, identifying and analyzing genes involved in phytohormone signaling in castor bean not only enhances our understanding of plant resilience to drought but also opens possibilities for biotechnological applications. This study could lead to the identification of new genetic targets for improving drought resistance in crops, making them more adaptable to changing environmental conditions.

## 5. Materials and Methods

### 5.1. Gene Identification

The sequence of the castor bean proteins was retrieved at Phytozome (https://phytozome-next.jgi.doe.gov/info/Rcommunis_v0_1 (accessed on 26 June 2024)) through the BLASTp tool using sequences from *Arabidopsis thaliana* as bait (Tables S4–S6), and a minimum threshold cut-off of e-value < 10^-10^ Subsequently, the sequences were checked by reverse BLASTp at NCBI and Pfam analysis to confirm the presence of conserved domains.

### 5.2. Phylogenetic Analysis

Phylogenetic trees were constructed using protein sequences from castor bean, Arabidopsis, and other species (Table S3). Sequences were aligned on MEGA version 11 using the Multiple Sequence Comparison by Log Expectation tool (MUSCLE) [96], and the phylogenetic tree was constructed individually for each family using the maximum likelihood method with 1000 replicates of bootstrap and aLRT statistics on the iQtree v1.6.12 program [97].

### 5.3. Gene Structure, Chromosomal Positions, and Gene Duplications Analysis

Exons/introns analysis was performed on the Gene Structure Display Server (GSDS 2.0) [98]. The position of the identified genes on the *R. communis* chromosomes was shown by CIRCOS [99]. Detection of putative gene duplication events was performed with MCScanX (E-value 1 × 10^−10^) and visualized using Advanced Circos of TBtools software v1.098769 [100]. Tandem duplication events were defined as two or more homologous genes located on a chromosomal region within 200 kb [101]. The nucleotide and amino acid sequences of duplicated gene pairs were aligned, and the number of non-synonymous substitutions per non-synonymous site (Ka), synonymous substitutions per synonymous site (Ks), and Ka/Ks ratio were estimated using KaKs_Calculator 2.0 software [102]. The divergence time was calculated according to T = Ks/(2 × 8.1 × 10^−9^) million years ago (MYA) for vascular plants [103].

### 5.4. Prediction of Cis-Regulatory Elements

The putative regulatory region (1 kb of the genomic sequence upstream from the translation start codon) of genes from castor beans were retrieved from the Phytozome v13 database, and the presence of *cis-*regulatory elements was identified by Plant Promoter Analysis, PlantCare ([104], accessed on 3 September 2024).

### 5.5. Identification of miRNA Targets

Castor bean miRNA sequences obtained from the sRNAano database [105] were utilized as input in the psRNAtarget tool [106] to search for possible targets among the specified transcripts, using the default settings, both accessed on 24 September 2024. Networks of miRNA-target interactions drawn from the psRNAtarget output were constructed using Cytoscape 3.10.3.

### 5.6. Protein Analysis In Silico

Molecular weight (MW), isoelectric point (pI), and GRAVY (grand average of hydropathy) from proteins identified were investigated through the ProtParam tool [107] (accessed on 20 July 2024). The conserved motifs in amino acid sequences were analysed using MEME (Multiple Em for Motif Elicitation) software (http://meme-suite.org/ (accessed on 23 September 2024) using the following parameters: number of motifs 1–15 and motif width of 5–50 [108].

### 5.7. Plant Material and RNA-Seq Analysis

Plants were sown in 15 L plastic pots with sandy loam soil and grown under continuous irrigation and natural photoperiod up to the expansion of the third pair of leaves (approximately 2 months), and then a suspension of the irrigation protocol was employed. The plants were divided randomly into two groups: a control group in which irrigation was continued, and a group in which irrigation was suspended until a water potential of mild (−0.5 MPa), moderate (−1.0 MPa), and severe (−1.5 MPa) was reached as previously described [8]. Drought stress levels were defined according to [109]. Six plants were used for each condition in the experiment. The control and water-deficit-treated plants were collected at the same time. The tissues were immediately frozen in liquid nitrogen and stored at −80 °C until processing. Frozen samples were ground in liquid nitrogen. The RNeasy Plant Mini Kit (Qiagen, Hilden, Germany) was used, and >10 μg RNA was used for each sample. The expression profile of castor bean leaves and roots in response to moderate drought (−1.0 MPa) was retrieved from the RNA-Seq experiment [8]. The expression data were expressed in heat maps using a Log2 scale with relative values to gene expression average, and these values were clustered using TBtools software v1.098769 [100].

### 5.8. Reverse Transcriptase and Quantitative PCR (RT-qPCR) Analysis

Complementary DNA (cDNA) was synthesized from 1 μg of total RNA using the SuperScript III Reverse Transcriptase (Invitrogen, (Thermo Fisher Scientific, Waltham, MA, USA)) and a 24-polyTV primer (Invitrogen^®,^ Thermo Fisher Scientific, Waltham, MA, USA). The qPCR reactions were performed with three biological and four technical replicates in the QuantStudio 12K system (Thermo Fisher Scientific, Waltham, MA, USA). The reaction mixture contained 2.5 μM of diluted cDNA, 0.3 μM of each primer, and SYBR^®^ Selection Master Mix (Applied Biosystems, Thermo Fisher Scientific, Waltham, MA, USA), in a total volume of 20 μL. The sequences of each primer used in RT-qPCR experiments are indicated in Table S12. The reaction mixtures were incubated for 2 min at 50 °C and then 5 min at 95 °C; this was followed by 40 amplification cycles consisting of 15 s at 95 °C and 20 s at 60 °C. Analyses of melting curves were performed immediately after the completion of the RT-qPCR to detect primer dimerization and nonspecific amplification. The data analysis was performed after comparative quantification using the 2^ΔΔCt^ method [110,111].

## Data Availability

Data are contained within the article and supplementary materials.

## Supplementary Materials

The following supporting information can be downloaded at: https://www.mdpi.com/article/10.3390/plants14081256/s1, Table S1: Physicochemical parameters from *Ricinus communis* proteins encoded by genes from gibberellin signaling; Table S2: Physicochemical parameters from *Ricinus communis* proteins encoded by from genes of jasmonate signaling; Table S3: Physicochemical parameters from *Ricinus communis* proteins encoded by genes from auxin signaling; Table S4: Genes of receptors GID1, SLY/SNE, and COI1/TIR1 organized by species; Table S5: Genes of repressor proteins DELLA, JAZ, and IAAs organized by species; Table S6: Genes of transcription factors PIF, BEH, MYC, and ARF organized by species; Table S7: Ka/Ks analysis and divergence time between the duplicated gene pairs; Table S8: Linked genes between *A. thaliana* and *R. communis*; Table S9: Conserved miRNAs targeting the genes from gibberellin signaling in *Ricinus communis*; Table S10: Conserved miRNAs targeting the genes from jasmonate signaling in *Ricinus communis*; Table S11: Conserved miRNAs targeting the genes from auxin signaling in *Ricinus communis*; Table S12: Primers used to qPCR reactions. Figure S1: Maximum-likelihood phylogenetic analysis of GID1 gene family; Figure S2: Maximum-likelihood phylogenetic analysis of COI1/TIR1 gene family; Figure S3: Maximum-likelihood phylogenetic analysis of SLY/SNE gene family; Figure S4: Maximum-likelihood phylogenetic analysis of DELLA gene family; Figure S5: Maximum-likelihood phylogenetic analysis of JAZ gene family; Figure S6: Maximum-likelihood phylogenetic analysis of IAA gene family; Figure S7: Maximum-likelihood phylogenetic analysis of PIF gene family; Figure S8: Maximum-likelihood phylogenetic analysis of BEH gene family; Figure S9: Maximum-likelihood phylogenetic analysis of MYC gene family; Figure S10: Maximum-likelihood phylogenetic analysis of ARF gene family; Figure S11: Analysis of the exon-intron structure and conserved motifs of receptors GID1, COI1/TIR1, and SLY/SNE; Figure S12: Analysis of the exon-intron structure and conserved motifs of inhibitor proteins DELLA, JAZ, and IAA; Figure S13: Analysis of the exon-intron structure and conserved motifs of transcription factors PIF, BEH, and MYC; Figure S14: Analysis of the exon-intron structure and conserved motifs of the transcription factor ARF.

## References

[1] FAO (2021). The State of the World’s Land and Water Resources for Food and Agriculture—Systems at Breaking Point (SOLAW 2021).

[2] Gupta A., Rico-Medina A., Caño-Delgado A.I. (2020). The Physiology of Plant Responses to Drought. Science.

[3] Calvin K., Dasgupta D., Krinner G., Mukherji A., Thorne P.W., Trisos C., Romero J., Aldunce P., Barrett K., Blanco G. (2023). IPCC, 2023: Climate Change 2023: Synthesis Report. Contribution of Working Groups I, II and III to the Sixth Assessment Report of the Intergovernmental Panel on Climate Change.

[4] Salvi P., Manna M., Kaur H., Thakur T., Gandass N., Bhatt D., Muthamilarasan M. (2021). Phytohormone Signaling and Crosstalk in Regulating Drought Stress Response in Plants. Plant Cell Rep..

[5] Browse J., Wallis J.G. (2019). Arabidopsis Flowers Unlocked the Mechanism of Jasmonate Signaling. Plants.

[6] Nelson S.K., Steber C.M. (2017). Transcriptional Mechanisms Associated with Seed Dormancy and Dormancy Loss in the Gibberellin-Insensitive Sly1-2 Mutant of *Arabidopsis thaliana*. PLoS ONE.

[7] Yang D.L., Yao J., Mei C.S., Tong X.H., Zeng L.J., Li Q., Xiao L.T., Sun T.P., Li J., Deng X.W. (2012). Plant Hormone Jasmonate Prioritizes Defense over Growth by Interfering with Gibberellin Signaling Cascade. Proc. Natl. Acad. Sci. USA.

[8] Jardim-Messeder D., Cassol D., Souza-Vieira Y., Ehlers Loureiro M., Girke T., Boroni M., Lopes Corrêa R., Coelho A., Sachetto-Martins G. (2023). Genome-Wide Identification of Core Components of ABA Signaling and Transcriptome Analysis Reveals Gene Circuits Involved in Castor Bean (*Ricinus communis L*.) Response to Drought. Gene.

[9] Jardim-Messeder D., de Souza-Vieira Y., Sachetto-Martins G. (2025). Dressed Up to the Nines: The Interplay of Phytohormones Signaling and Redox Metabolism During Plant Response to Drought. Plants.

[10] Olszewski N., Sun T.P., Gubler F. (2002). Gibberellin Signaling: Biosynthesis, Catabolism, and Response Pathways. Plant Cell.

[11] Gazara R.K., Moharana K.C., Bellieny-Rabelo D., Venancio T.M. (2018). Expansion and Diversification of the Gibberellin Receptor GIBBERELLIN INSENSITIVE DWARF1 (GID1) Family in Land Plants. Plant Mol. Biol..

[12] Murase K., Hirano Y., Sun T.P., Hakoshima T. (2008). Gibberellin-Induced DELLA Recognition by the Gibberellin Receptor GID1. Nature.

[13] Ariizumi T., Lawrence P.K., Steber C.M. (2011). The Role of Two F-Box Proteins, SLEEPY1 and SNEEZY, in Arabidopsis Gibberellin Signaling. Plant Physiol..

[14] Hartweck L.M. (2008). Gibberellin Signaling. Planta.

[15] Liu K., Li Y., Chen X., Li L., Liu K., Zhao H., Wang Y., Han S. (2018). ERF72 Interacts with ARF6 and BZR1 to Regulate Hypocotyl Elongation in Arabidopsis. J. Exp. Bot..

[16] Colebrook E.H., Thomas S.G., Phillips A.L., Hedden P. (2014). The Role of Gibberellin Signalling in Plant Responses to Abiotic Stress. J. Exp. Biol..

[17] Jogawat A. (2019). Crosstalk Among Phytohormone Signaling Pathways During Abiotic Stress. Molecular Plant Abiotic Stress: Biology and Biotechnology.

[18] Lee H.Y., Seo J.S., Cho J.H., Jung H., Kim J.K., Lee J.S., Rhee S., Do Choi Y. (2013). Oryza Sativa COI Homologues Restore Jasmonate Signal Transduction in Arabidopsis Coi1-1 Mutants. PLoS ONE.

[19] Sheard L.B., Tan X., Mao H., Withers J., Ben-Nissan G., Hinds T.R., Kobayashi Y., Hsu F.F., Sharon M., Browse J. (2010). Jasmonate Perception by Inositol-Phosphate-Potentiated COI1-JAZ Co-Receptor. Nature.

[20] Yan J., Yao R., Chen L., Li S., Gu M., Nan F., Xie D. (2018). Dynamic Perception of Jasmonates by the F-Box Protein COI1. Mol. Plant.

[21] Major I.T., Yoshida Y., Campos M.L., Kapali G., Xin X.F., Sugimoto K., de Oliveira Ferreira D., He S.Y., Howe G.A. (2017). Regulation of Growth–Defense Balance by the JASMONATE ZIM-DOMAIN (JAZ)-MYC Transcriptional Module. New Phytol..

[22] Ishiguro S., Kawai-Oda A., Ueda J., Nishida I., Okada K. (2001). The Defective in Anther Dehiscence1 gene Encodes a Novel Phospholipase A1 Catalyzing the Initial Step of Jasmonic Acid Biosynthesis, Which Synchronizes Pollen Maturation, Anther Dehiscence, and Flower Opening in Arabidopsis. Plant Cell.

[23] Howe G.A., Major I.T., Koo A.J. (2018). Modularity in Jasmonate Signaling for Multistress Resilience. Annu. Rev. Plant Biol..

[24] Kazan K., Manners J.M. (2011). The Interplay between Light and Jasmonate Signalling during Defence and Development. J. Exp. Bot..

[25] Hou X., Lee L.Y.C., Xia K., Yan Y., Yu H. (2010). DELLAs Modulate Jasmonate Signaling via Competitive Binding to JAZs. Dev. Cell.

[26] Lavy M., Estelle M. (2016). Mechanisms of Auxin Signaling. Dev. Camb..

[27] Takatsuka H., Umeda M. (2014). Hormonal Control of Cell Division and Elongation along Differentiation Trajectories in Roots. J. Exp. Bot..

[28] Sharma E., Sharma R., Borah P., Jain M., Khurana J.P. (2015). Emerging Roles of Auxin in Abiotic Stress Responses. Elucidation of Abiotic Stress Signaling in Plants: Functional Genomics Perspectives.

[29] Wolf J., Weiss E.A. (2000). Oilseed Crops.

[30] Audran-Delalande C., Bassa C., Mila I., Regad F., Zouine M., Bouzayen M. (2012). Genome-Wide Identification, Functional Analysis and Expression Profiling of the Aux/IAA Gene Family in Tomato. Plant Cell Physiol..

[31] Dreher K.A., Brown J., Saw R.E., Callis J. (2006). The Arabidopsis Aux/IAA Protein Family Has Diversified in Degradation and Auxin Responsiveness. Plant Cell.

[32] Guo Y., Wu H., Li X., Li Q., Zhao X., Duan X., An Y., Lv W., An H. (2017). Identification and Expression of GRAS Family Genes in Maize (*Zea mays* L.). PLoS ONE.

[33] Jain M., Kaur N., Garg R., Thakur J.K., Tyagi A.K., Khurana J.P. (2006). Structure and Expression Analysis of Early Auxin-Responsive Aux/IAA Gene Family in Rice (*Oryza sativa*). Funct. Integr. Genom..

[34] Niu Y., Figueroa P., Browse J. (2011). Characterization of JAZ-Interacting bHLH Transcription Factors That Regulate Jasmonate Responses in Arabidopsis. J. Exp. Bot..

[35] Tian C., Wan P., Sun S., Li J., Chen M. (2004). Genome-Wide Analysis of the GRAS Gene Family in Rice and Arabidopsis. Plant Mol. Biol..

[36] Toledo-Ortiz G., Huq E., Quail P.H. (2003). The Arabidopsis Basic/Helix-Loop-Helix Transcription Factor Family. Plant Cell.

[37] Um T.Y., Lee H.Y., Lee S., Chang S.H., Chung P.J., Oh K.B., Kim J.K., Jang G., Choi Y.D. (2018). Jasmonate Zim-Domain Protein 9 Interacts with Slender Rice 1 to Mediate the Antagonistic Interaction between Jasmonic and Gibberellic Acid Signals in Rice. Front. Plant Sci..

[38] Wang Y., Qiao L., Bai J., Wang P., Duan W., Yuan S., Yuan G., Zhang F., Zhang L., Zhao C. (2017). Genome-Wide Characterization of JASMONATE-ZIM DOMAIN Transcription Repressors in Wheat (*Triticum aestivum* L.). BMC Genom..

[39] Ye M., Luo S.M., Xie J.F., Li Y.F., Xu T., Liu Y., Song Y.Y., Zhu-Salzman K., Zeng R.S. (2012). Silencing COI1 in Rice Increases Susceptibility to Chewing Insects and Impairs Inducible Defense. PLoS ONE.

[40] Zhang Y., Mayba O., Pfeiffer A., Shi H., Tepperman J.M., Speed T.P., Quail P.H. (2013). A Quartet of PIF bHLH Factors Provides a Transcriptionally Centered Signaling Hub That Regulates Seedling Morphogenesis through Differential Expression-Patterning of Shared Target Genes in Arabidopsis. PLoS Genet..

[41] Shrirame H.Y., Panwar N.L., Bamniya B.R. (2011). Bio Diesel from Castor Oil—A Green Energy Option. Low Carbon Econ..

[42] Baldwin B.S., Cossar R.D. (2009). Castor Yield in Response to Planting Date at Four Locations in the South-Central United States. Ind. Crops Prod..

[43] Feng L., Li G., He Z., Han W., Sun J., Huang F., Di J., Chen Y. (2019). The ARF, GH3, and Aux/IAA Gene Families in Castor Bean (*Ricinus communis* L.): Genome-Wide Identification and Expression Profiles in High-Stalk and Dwarf Strains. Ind. Crops Prod..

[44] Fernández-Calvo P., Chini A., Fernández-Barbero G., Chico J.M., Gimenez-Ibanez S., Geerinck J., Eeckhout D., Schweizer F., Godoy M., Franco-Zorrilla J.M. (2011). The Arabidopsis bHLH Transcription Factors MYC3 and MYC4 Are Targets of JAZ Repressors and Act Additively with MYC2 in the Activation of Jasmonate Responses. Plant Cell.

[45] Gagne J.M., Downes B.P., Shiu S.-H., Durski A.M., Vierstra R.D. (2002). The F-Box Subunit of the SCF E3 Complex Is Encoded by a Diverse Superfamily of Genes in Arabidopsis. Proc. Natl. Acad. Sci. USA.

[46] Garrido-Bigotes A., Valenzuela-Riffo F., Figueroa C.R. (2019). Evolutionary Analysis of JAZ Proteins in Plants: An Approach in Search of the Ancestral Sequence. Int. J. Mol. Sci..

[47] Lee M.H., Kim B., Song S.K., Heo J.O., Yu N.I., Lee S.A., Kim M., Kim D.G., Sohn S.O., Lim C.E. (2008). Large-Scale Analysis of the GRAS Gene Family in *Arabidopsis thaliana*. Plant Mol. Biol..

[48] Ji Z., Belfield E.J., Zhang S., Bouvier J., Li S., Schnell J., Fu X., Harberd N.P. (2023). Evolution of a Plant Growth-Regulatory Protein Interaction Specificity. Nat. Plants.

[49] Reinhold H., Soyk S., Šimková K., Hostettler C., Marafino J., Mainiero S., Vaughan C.K., Monroe J.D., Zeeman S.C. (2011). β-Amylase–Like Proteins Function as Transcription Factors in Arabidopsis, Controlling Shoot Growth and Development[C][W][OA]. Plant Cell.

[50] Mutte S.K., Kato H., Rothfels C., Melkonian M., Wong G.K.-S., Weijers D. Origin and Evolution of the Nuclear Auxin Response System. https://elifesciences.org/articles/33399/figures.

[51] Yoshida H., Tanimoto E., Hirai T., Miyanoiri Y., Mitani R., Kawamura M., Takeda M., Takehara S., Hirano K., Kainosho M. (2018). Evolution and Diversification of the Plant Gibberellin Receptor GID1. Proc. Natl. Acad. Sci. USA.

[52] Phokas A., Coates J.C. (2021). Evolution of DELLA Function and Signaling in Land Plants. Evolution and Development.

[53] Sun T. (2011). The Molecular Mechanism and Evolution of the GA–GID1–DELLA Signaling Module in Plants. Curr. Biol..

[54] Qi L., Kwiatkowski M., Chen H., Hoermayer L., Sinclair S., Zou M., del Genio C.I., Kubeš M.F., Napier R., Jaworski K. (2022). Adenylate Cyclase Activity of TIR1/AFB Auxin Receptors in Plants. Nature.

[55] Santner A., Estelle M. (2010). The Ubiquitin-Proteasome System Regulates Plant Hormone Signaling. Plant J..

[56] Pauwels L., Goossens A. (2011). The JAZ Proteins: A Crucial Interface in the Jasmonate Signaling Cascade. Plant Cell.

[57] Calderón Villalobos L.I.A., Lee S., De Oliveira C., Ivetac A., Brandt W., Armitage L., Sheard L.B., Tan X., Parry G., Mao H. (2012). A Combinatorial TIR1/AFB–Aux/IAA Co-Receptor System for Differential Sensing of Auxin. Nat. Chem. Biol..

[58] Nelson S.K., Steber C.M. (2016). Gibberellin Hormone Signal Perception: Down-Regulating DELLA Repressors of Plant Growth and Development. Annual Plant Reviews: The Gibberellins.

[59] Yang Y., Guang Y., Wang F., Chen Y., Yang W., Xiao X., Luo S., Zhou Y. (2021). Characterization of Phytochrome-Interacting Factor Genes in Pepper and Functional Analysis of CaPIF8 in Cold and Salt Stress. Front. Plant Sci..

[60] Yin Y., Vafeados D., Tao Y., Yoshida S., Asami T., Chory J. (2005). A New Class of Transcription Factors Mediates Brassinosteroid-Regulated Gene Expression in Arabidopsis. Cell.

[61] Song C., Cao Y., Dai J., Li G., Manzoor M.A., Chen C., Deng H. (2022). The Multifaceted Roles of MYC2 in Plants: Toward Transcriptional Reprogramming and Stress Tolerance by Jasmonate Signaling. Front. Plant Sci..

[62] Wang R., Estelle M. (2014). Diversity and Specificity: Auxin Perception and Signaling through the TIR1/AFB Pathway. Curr. Opin. Plant Biol..

[63] Song S., Qi T., Huang H., Xie D. (2013). Regulation of Stamen Development by Coordinated Actions of Jasmonate, Auxin, and Gibberellin in Arabidopsis. Mol. Plant.

[64] Bai M.Y., Shang J.X., Oh E., Fan M., Bai Y., Zentella R., Sun T.P., Wang Z.Y. (2012). Brassinosteroid, Gibberellin and Phytochrome Impinge on a Common Transcription Module in Arabidopsis. Nat. Cell Biol..

[65] Ahmad H.M., Wang X., Ijaz M., Mahmood-Ur-Rahman, Oranab S., Ali M.A., Fiaz S. (2022). Molecular Aspects of MicroRNAs and Phytohormonal Signaling in Response to Drought Stress: A Review. Curr. Issues Mol. Biol..

[66] Jardim-Messeder D., de Souza-Vieira Y., Lavaquial L.C., Cassol D., Galhego V., Bastos G.A., Felix-Cordeiro T., Corrêa R.L., Zámocký M., Margis-Pinheiro M. (2022). Ascorbate-Glutathione Cycle Genes Families in Euphorbiaceae: Characterization and Evolutionary Analysis. Biology.

[67] de Souza-Vieira Y., Felix-Mendes E., Galhego V., Bastos G.A., Felix-Cordeiro T., Ding X., Zhang Y., Corrêa R.L., Wang X., Sachetto-Martins G. (2024). Euphorbiaceae Superoxide Dismutase, Catalase, and Glutathione Peroxidase as Clues to Better Comprehend High Drought Tolerance in Castor Bean. Ind. Crops Prod..

[68] Illouz-Eliaz N., Nissan I., Nir I., Ramon U., Shohat H., Weiss D. (2020). Mutations in the Tomato Gibberellin Receptors Suppress Xylem Proliferation and Reduce Water Loss under Water-Deficit Conditions. J. Exp. Bot..

[69] Xu W., Chen Z., Ahmed N., Han B., Cui Q., Liu A. (2016). Genome-Wide Identification, Evolutionary Analysis, and Stress Responses of the GRAS Gene Family in Castor Beans. Int. J. Mol. Sci..

[70] Yasumura Y., Crumpton-Taylor M., Fuentes S., Harberd N.P. (2007). Step-by-Step Acquisition of the Gibberellin-DELLA Growth-Regulatory Mechanism during Land-Plant Evolution. Curr. Biol..

[71] Huang X., Tian H., Park J., Oh D.H., Hu J., Zentella R., Qiao H., Dassanayake M., Sun T.P. (2023). The Master Growth Regulator DELLA Binding to Histone H2A Is Essential for DELLA-Mediated Global Transcription Regulation. Nat. Plants.

[72] Saud S., Shi Z., Xiong L., Danish S., Datta R., Ahmad I., Fahad S., Banout J. (2022). Recognizing the Basics of Phytochrome-Interacting Factors in Plants for Abiotic Stress Tolerance. Plant Stress.

[73] Cordeiro A.M., Andrade L., Monteiro C.C., Leitão G., Wigge P.A., Saibo N.J.M. (2022). Phytochrome-Interacting Factors: A Promising Tool to Improve Crop Productivity. J. Exp. Bot..

[74] Wang X.R., Wang Y.H., Jia M., Zhang R.R., Liu H., Xu Z.S., Xiong A.S. (2022). The Phytochrome-Interacting Factor DcPIF3 of Carrot Plays a Positive Role in Drought Stress by Increasing Endogenous ABA Level in Arabidopsis. Plant Sci..

[75] Kudo M., Kidokoro S., Yoshida T., Mizoi J., Todaka D., Fernie A.R., Shinozaki K., Yamaguchi-Shinozaki K. (2017). Double Overexpression of DREB and PIF Transcription Factors Improves Drought Stress Tolerance and Cell Elongation in Transgenic Plants. Plant Biotechnol. J..

[76] Liu S., Zhang Y., Pan X., Li B., Yang Q., Yang C., Zhang J., Wu F., Yang A., Li Y. (2023). PIF1, a Phytochrome-Interacting Factor Negatively Regulates Drought Tolerance and Carotenoids Biosynthesis in Tobacco. Int. J. Biol. Macromol..

[77] Cui X.Y., Gao Y., Guo J., Yu T.F., Zheng W.J., Liu Y.W., Chen J., Xu Z.S., Ma Y.Z. (2019). BES/BZR Transcription Factor TaBZR2 Positively Regulates Drought Responses by Activation of TaGST1. Plant Physiol..

[78] Ye H., Liu S., Tang B., Chen J., Xie Z., Nolan T.M., Jiang H., Guo H., Lin H.Y., Li L. (2017). RD26 Mediates Crosstalk between Drought and Brassinosteroid Signalling Pathways. Nat. Commun..

[79] Wang L., Lin M., Zou L., Zhang S., Lan Y., Yan H., Xiang Y. (2024). Comprehensive Investigation of BZR Gene Family in Four Dicots and the Function of *PtBZR9* and *PtBZR12* under Drought Stress. Plant Physiol. Biochem..

[80] Thines B., Katsir L., Melotto M., Niu Y., Mandaokar A., Liu G., Nomura K., He S.Y., Howe G.A., Browse J. (2007). JAZ Repressor Proteins Are Targets of the SCFCOI1 Complex during Jasmonate Signalling. Nature.

[81] Kim J.M., To T.K., Matsui A., Tanoi K., Kobayashi N.I., Matsuda F., Habu Y., Ogawa D., Sakamoto T., Matsunaga S. (2017). Acetate-Mediated Novel Survival Strategy against Drought in Plants. Nat. Plants.

[82] Waadt R., Seller C.A., Hsu P.K., Takahashi Y., Munemasa S., Schroeder J.I. (2022). Plant Hormone Regulation of Abiotic Stress Responses. Nat. Rev. Mol. Cell Biol..

[83] Fu J., Wu H., Ma S., Xiang D., Liu R., Xiong L. (2017). OSJAZ1 Attenuates Drought Resistance by Regulating JA and ABA Signaling in Rice. Front. Plant Sci..

[84] Liu B., Seong K., Pang S., Song J., Gao H., Wang C., Zhai J., Zhang Y., Gao S., Li X. (2021). Functional Specificity, Diversity, and Redundancy of Arabidopsis JAZ Family Repressors in Jasmonate and COI1-Regulated Growth, Development, and Defense. New Phytol..

[85] Aleman F., Yazaki J., Lee M., Takahashi Y., Kim A.Y., Li Z., Kinoshita T., Ecker J.R., Schroeder J.I. (2016). An ABA-Increased Interaction of the PYL6 ABA Receptor with MYC2 Transcription Factor: A Putative Link of ABA and JA Signaling. Sci. Rep..

[86] Kazan K., Manners J.M. (2013). MYC2: The Master in Action. Mol. Plant.

[87] Jing H., Wilkinson E.G., Sageman-Furnas K., Strader L.C. (2023). Auxin and Abiotic Stress Responses. J. Exp. Bot..

[88] Du W., Lu Y., Li Q., Luo S., Shen S., Li N., Chen X. (2022). TIR1/AFB Proteins: Active Players in Abiotic and Biotic Stress Signaling. Front. Plant Sci..

[89] Salehin M., Li B., Tang M., Katz E., Song L., Ecker J.R., Kliebenstein D.J., Estelle M. (2019). Auxin-Sensitive Aux/IAA Proteins Mediate Drought Tolerance in Arabidopsis by Regulating Glucosinolate Levels. Nat. Commun..

[90] Liu P.-P., Montgomery T.A., Fahlgren N., Kasschau K.D., Nonogaki H., Carrington J.C. (2007). Repression of AUXIN RESPONSE FACTOR10 by microRNA160 Is Critical for Seed Germination and Post-Germination Stages. Plant J..

[91] Liu H.-H., Tian X., Li Y.-J., Wu C.-A., Zheng C.-C. (2008). Microarray-Based Analysis of Stress-Regulated microRNAs in Arabidopsis Thaliana. RNA.

[92] Zhang F., Yang J., Zhang N., Wu J., Si H. (2022). Roles of microRNAs in Abiotic Stress Response and Characteristics Regulation of Plant. Front. Plant Sci..

[93] Arshad M., Gruber M.Y., Hannoufa A. (2018). Transcriptome Analysis of microRNA156 Overexpression Alfalfa Roots under Drought Stress. Sci. Rep..

[94] Singroha G., Sharma P., Sunkur R. (2021). Current Status of microRNA-Mediated Regulation of Drought Stress Responses in Cereals. Physiol. Plant..

[95] Ferdous J., Hussain S.S., Shi B.-J. (2015). Role of microRNAs in Plant Drought Tolerance. Plant Biotechnol. J..

[96] Edgar R.C. (2004). MUSCLE: Multiple Sequence Alignment with High Accuracy and High Throughput. Nucleic Acids Res..

[97] Nguyen L.T., Schmidt H.A., Von Haeseler A., Minh B.Q. (2015). IQ-TREE: A Fast and Effective Stochastic Algorithm for Estimating Maximum-Likelihood Phylogenies. Mol. Biol. Evol..

[98] Hu B., Jin J., Guo A.Y., Zhang H., Luo J., Gao G. (2015). GSDS 2.0: An Upgraded Gene Feature Visualization Server. Bioinformatics.

[99] Rzywinski M., Schein J., Birol I., Connors J., Gascoyne R., Horsman D., Jones S.J., Marra M.A. (2009). Circos: An Information Aesthetic for Comparative Genomics. Genome Res..

[100] Chen C., Chen H., Zhang Y., Thomas H.R., Frank M.H., He Y., Xia R. (2020). TBtools: An Integrative Toolkit Developed for Interactive Analyses of Big Biological Data. Mol. Plant.

[101] Wang Y., Tang H., Debarry J.D., Tan X., Li J., Wang X., Lee T.H., Jin H., Marler B., Guo H. (2012). MCScanX: A Toolkit for Detection and Evolutionary Analysis of Gene Synteny and Collinearity. Nucleic Acids Res..

[102] Wang D., Zhang Y., Zhang Z., Zhu J., Yu J. (2010). KaKs_Calculator 2.0: A Toolkit Incorporating Gamma-Series Methods and Sliding Window Strategies. Genom. Proteom. Bioinform..

[103] Shevliakova E., Sarmiento J., Gloor M., Lynch M., Conery J.S. (2000). The Evolutionary Fate and Consequences of Duplicate Genes. Science.

[104] Lescot M., Déhais P., Thijs G., Marchal K., Moreau Y., Van De Peer Y., Rouzé P., Rombauts S. (2002). PlantCARE, a Database of Plant Cis-Acting Regulatory Elements and a Portal to Tools for in Silico Analysis of Promoter Sequences. Nucleic Acids Res..

[105] Chen C., Li J., Feng J., Liu B., Feng L., Yu X., Li G., Zhai J., Meyers B.C., Xia R. (2021). sRNAanno—A Database Repository of Uniformly Annotated Small RNAs in Plants. Hortic. Res..

[106] Dai X., Zhuang Z., Zhao P.X. (2018). psRNATarget: A Plant Small RNA Target Analysis Server (2017 Release). Nucleic Acids Res..

[107] Gasteiger E., Hoogland C., Gattiker A., Duvaud S., Wilkins M.R., Appel R.D., Bairoch A. (2005). Protein Identification and Analysis Tools on the ExPASy Server. The Proteomics Protocols Handbook.

[108] Bailey T.L., Boden M., Buske F.A., Frith M., Grant C.E., Clementi L., Ren J., Li W.W., Noble W.S. (2009). MEME Suite: Tools for Motif Discovery and Searching. Nucleic Acids Res..

[109] Hsiao T.C. (1973). Plant Responses to Water Stress. Annu. Rev. Plant Physiol..

[110] Livak K.J., Schmittgen T.D. (2001). Analysis of Relative Gene Expression Data Using Real-Time Quantitative PCR and the 2^−ΔΔCT^ Method. Methods.

[111] Schmittgen T.D., Livak K.J. (2008). Analyzing Real-Time PCR Data by the Comparative CT Method. Nat. Protoc..

