# Supplementary material for: Analysis of the Genes from Gibberellin, Jasmonate, and Auxin Signaling Under Drought Stress: A Genome-Wide Approach in Castor Bean (Ricinus communis L.)
Source: Plants (Basel). 2025 Apr 20;14(8):1256. doi: 10.3390/plants14081256 (PMC12030089; doi:10.3390/plants14081256)
Supplement: Supplementary file 1 [file plants-14-01256-s001.zip › plants-3569207-supplementary.pdf]

Table S1

Table S1. Physicochemical parameters from *R. communis* proteins encoded by genes from gibberellin signaling. Molecular weight (MW), isoelectric point (pI), and GRAVY (grand average of hydropathy) from proteins identified were investigated through the ProtParam tool

	Gene Name	Trancript Code ID (phytozome)	Protein ID (NCBI)	Sequence Length	Molecular Weight	Isoelectric Point (pI)	GRAVY	Instability Index
GA	RcGID1b	29703.m001506	XP_002524767.2	345	39126.9	6.420	-0.237	42.2
	RcGID1c	30128.m008695	XP_002512310.1	344	38715.7	6.323	-0.200	42.6
	RcDELLA1	29692.m000540	XP_015580029.3	609	67287.1	5.726	-0.317	45.1
	RcDELLA2	30170.m013590	XP_002510069.1	471	53788.0	5.684	-0.296	49.0
	RcDELLA3	28677.m000055	XP_002534030.1	567	62549.7	5.141	-0.327	49.9
	RcDELLA4	29807.m000482	XP_002529354.2	536	58616.0	5.268	-0.049	44.1
	RcSLY	30170.m014210	XP_002510145.1	179	20473.0	9.675	-0.683	66.7
	RcSNE	28859.m000331	XP_002530225.1	155	17730.5	9.393	-0.059	66.5
	RcPIF1	30204.m001816	XP_002521150.1	572	62603.8	5.954	-0.777	60.9
	RcPIF2	30076.m004542	XP_002515170.2	584	64257.2	8.820	-0.509	59.1
	RcPIF3	30128.m008988	XP_015570501.2	758	80867.5	5.870	-0.552	52.6
	RcPIF8	30174.m008814	XP_015582839.1	465	50489.4	6.969	-0.540	58.9
	RcPIF9	29737.m001209	XP_002523493.1	474	49714.7	6.400	-0.523	49.0
	RcALC	30170.m013852	XP_002510190.2	312	33589.9	5.336	-0.557	71.6
	RcSPT	30115.m001235	XP_015577491.1	406	44826.7	5.750	-0.441	62.6
	RcBEH1	30170.m013999	XP_002510347.1	321	34856.5	9.190	-0.618	64.6
	RcBEH2	29646.m001062	XP_002525100.1	317	34472.1	9.180	-0.647	70.9
	RcBEH3	30026.m001485	XP_002522271.1	331	35458.0	8.436	-0.666	59.8
	RcBEH4	29820.m001005	XP_048226119.1	97	11050.7	10.857	-1.051	30.4
	RcBMY1	30174.m009027	XP_002511857.1	704	78913.3	5.782	-0.401	40.8
	RcBMY2	29827.m002636	XP_002519919.3	668	75469.7	5.774	-0.459	44.0

Table S2. Physicochemical parameters from *R. communis* proteins encoded by from genes of jasmonate signaling. Molecular weight (MW), isoelectric point (pI), and GRAVY (grand average of hydropathy) from proteins identified were investigated through the ProtParam tool.

	Gene Name	Trancript Code ID (phytozome)	Protein ID (NCBI)	Sequence Length	Molecular Weight	Isoelectric Point (pI)	GRAVY	Instability Index
	RcCOI1	29610.m000411	XP_002530419.3	602	68055.1	6.689	-0.072	44.0
	RcJAZ1	29693.m001988	XP_015574971.2	164	18906.5	9.923	-0.669	82.3
	RcJAZ2	30128.m009047	XP_002512644.1	177	19922.4	9.44	-0.553	50.6
	RcJAZ3	29739.m003727	XP_002516243.1	289	31121.8	9.493	-0.507	47.0
	RcJAZ4	29727.m000494	XP_002529200.1	269	29328.6	9.146	-0.688	52.8
	RcJAZ5	29765.m000754	XP_048231258.1	176	19594.0	9.625	-0.798	77.0
	RcJAZ6	29848.m004713	XP_002515600.2	446	46763.0	7.887	-0.627	42.8
	RcJAZ7	29739.m003659	XP_002516176.1	220	23417.2	8.5	-0.357	61.3
	RcJAZ8	29962.m000052	XP_015583781.2	374	40047.1	9.034	-0.219	51.5
	RcJAZ9	29653.m000290	XP_002530784.2	258	26636.6	9.436	-0.174	50.4
	RcMYC1	30174.m009029	XP_015584416.2	656	73954.7	5.37	-0.459	43.9
	RcMYC2	29827.m002528	XP_002519814.1	663	72630.8	5.481	-0.652	46.8
**JA**	RcMYC4	30068.m002581	XP_002518914.1	479	53909.5	6.077	-0.421	40.8
	RcMYC5	27964.m000356	XP_002529965.2	288	32177.9	5.641	-0.613	41.4
	RcMYC6	30190.m011223	XP_048230884.1	492	54834.2	5.637	-0.327	53.9
	RcMYC7	29742.m001404	XP_002523332.1	615	67942.3	6.036	-0.522	38.2
	RcMYC8	29883.m002023	XP_002521010.2	503	55499.7	5.818	-0.45	46.1
	RcMYC9	29647.m002101	XP_015575616.2	668	75005.8	5.533	-0.669	70.4
	RcMYC10	30076.m004456	XP_002515085.2	400	45859.0	5.334	-0.674	69.8
	RcMYC11	30128.m008737	XP_002512342.2	366	41196.7	6.35	-0.566	61.6
	RcMYC12	29908.m006098	XP_002513985.2	338	38185.3	5.72	-0.652	66.0
	RcMYC13	29904.m002956	XP_048234754.1	411	46915.3	5.953	-0.614	61.5
	RcMYC14	50006.m000011	XP_002539136.3	187	20769.4	6.087	-0.173	61.1
	RcMYC15	29844.m003312	XP_002517160.1	195	22052.2	8.693	-0.199	68.7
	RcMYC16	30054.m000819	XP_048230125.1	275	31546.8	8.563	-0.514	64.8

Table S3. Physicochemical parameters from *R. communis* proteins encoded by genes from auxin signaling. Molecular weight (MW), isoelectric point (pI), and GRAVY (grand average of hydropathy) from proteins identified were investigated through the ProtParam tool.

	Gene Name	Trancript Code ID (phytozome)	Protein ID (NCBI)	Sequence Length	Molecular Weight	Isoelectric Point (pI)	GRAVY	Instability Index
Aux	RcTIR1	29647.m002022	XP_002520681.1	585	65701.3	6.726	-0.018	53.4
	RcAFB2	30131.m006863	XP_002512866.1	571	64334.6	6.705	-0.071	40.7
	RcAFB1	29908.m006223	XP_002514006.1	635	70980.5	5.446	-0.187	46.8
	RcIAA1	28179.m000461	XP_002528895.1	382	41112.0	7.586	-0.458	40.9
	RcIAA2	29598.m000460	XP_002531288.2	257	27838.3	6.462	-0.492	37.9
	RcIAA3	29598.m000461	XP_002531289.1	200	22523.1	5.999	-0.716	38.5
	RcIAA4	29637.m000735	XP_015579293.2	300	31565.8	7.802	-0.436	48.7
	RcIAA5	29794.m003410	XP_048227500.1	367	40154.3	8.99	-0.708	56.5
	RcIAA6	29841.m002748	XP_002517528.1	194	21669.5	7.55	-0.588	38.9
	RcIAA7	29841.m002749	XP_002517529.1	217	24035.1	5.627	-0.504	46.7
	RcIAA8	29844.m003174	XP_002517023.3	181	20354.2	8.635	-0.586	44.2
	RcIAA9	29844.m003175	XP_002517024.1	191	21570.2	7.552	-0.809	53.6
	RcIAA10	29848.m004617	XP_015572505.2	895	98734.1	5.825	-0.233	37.3
	RcIAA11	29883.m001992	XP_002520979.1	196	21868.4	6.192	-0.688	48.0
	RcIAA12	29883.m001993	XP_002520980.1	244	26924.3	7.66	-0.614	41.9
	RcIAA13	29912.m005483	XP_002514266.1	297	32370.1	8.176	-0.655	48.4
	RcIAA14	29927.m000601	XP_015579675.2	250	28021.9	5.288	-0.662	41.7
	RcIAA15	30008.m000799	XP_002528346.2	318	33055.5	8.786	-0.464	37.1
	RcIAA16	30076.m004546	XP_002515174.1	175	19730.0	4.948	-0.383	45.2
	RcIAA17	30146.m003502	XP_002515959.3	320	35180.2	8.161	-0.625	51.6
	RcIAA18	30146.m003546	XP_002516003.1	373	39930.0	8.343	-0.581	42.3
	RcIAA19	30170.m013613	XP_048226901.1	374	40593.3	7.051	-0.448	40.5
	RcIAA20	28664.m000104	XP_002532238.1	173	19269.4	6.583	-0.486	56.2
	RcIAA21	29660.m000755	XP_015579134.1	192	21885.2	5.404	-0.49	48.4
	RcARF1	29769.m000463	XP_015579071.1	826	92325.4	5.98	-0.507	62.0
	RcARF2	27494.m000045	XP_002522498.2	590	65623.6	6.486	-0.38	47.5
	RcARF3	29609.m000587	XP_002527835.2	671	75133.5	5.656	-0.541	60.2
	RcARF4	30185.m000956	XP_002519813.1	1109	122192.8	6.226	-0.533	64.2
	RcARF5	29908.m005953	XP_015571564.2	603	66511.1	5.87	-0.294	53.5
	RcARF6	29609.m000586	XP_002527834.2	620	70439.4	6.025	-0.373	58.9
	RcARF7	29647.m002032	XP_048226330.1	634	69614.0	5.564	-0.424	47.1
	RcARF8	29844.m003175	XP_002517024.1	191	21570.2	7.552	-0.809	53.6
	RcARF9	30146.m003502	XP_002515959.3	320	35180.2	8.161	-0.625	51.6
	RcARF10	30170.m014161	XP_002510508.1	950	104627.1	5.123	-0.399	52.8
	RcARF11	30147.m014418	XP_048229217.1	844	93834.3	6.037	-0.574	53.2
	RcARF12	30111.m000734	XP_015579154.2	810	89963.3	6.388	-0.502	56.8
	RcARF13	29599.m000177	XP_048229801.1	478	53361.2	8.277	-0.344	58.6
	RcARF14	27538.m000327	XP_002529941.1	709	77977.1	7.575	-0.373	50.0
	RcARF15	29739.m003713	XP_002516230.1	1119	125132.3	6.122	-0.683	72.8
	RcARF16	30078.m002329	XP_002519531.1	702	77132.5	6.136	-0.377	48.3
	RcARF17	30128.m008545	XP_015570523.1	787	89006.7	6.123	-0.568	54.7
	RcARF18	30068.m002516	XP_002518850.2	694	77445.1	6.117	-0.539	58.6
	RcARF19	29742.m001397	XP_015577377.1	667	74783.8	6.847	-0.458	46.1
	RcARF20	29900.m001540	XP_002522498.2	730	80562.7	6.887	-0.431	61.8

Table S4. Genes of receptors GID1, SLY/SNE, and COI1/TIR1 organized by species. The table includes CodeID and the corresponding names assigned to the phylogenetic tree leaves.

Family	Species	Code ID	Name
GID1	*A. thaliana*	AT3G05120.1	AtGID1a
		AT3G63010.1	AtGID1b
		AT5G27320.1	AtGID1c
	S. lycopersicum	Solyc06g008870.2.1	SlGID1b1
		Solyc09g074270.2.1	SlGID1b2
		Solyc01g098390.2.1	SlGID1c
	M. esculenta	Manes.05G070500.1.p	MeGID1b1
		Manes.01G212300.1.p	MeGID1b2
		Manes.09G161600.1.p	MeGID1c1
		Manes.08G124700.1.p	MeGID1c2
	Z. mays	GRMZM2G173630_P01	ZmGID1_2
		GRMZM2G016605_P02	ZmGID1_1
	S. bicolor	Sobic.009G134600.1.p	SbGID1
	O. sativa	LOC_Os05g33730.1	OsGID1
SLY/SNE	*A. thaliana*	AT4G24210.1	AtSLY1
		AT5G48170.1	AtSNE
	B. napus	ACV88719	BnSLY1
	S. lycopersicum	Solyc04g078390	SlSLY1
		Solyc07g047680.3.1	SlSNE
	P. salicina	MT358313	PslSLY1
	V. vinifera	XP_003632510	VvGID2
	O. sativa	Q7XAK4	OsGID2
	Z. mays	NP_001149408	ZmGID2
COI1/TIR1	*A. thaliana*	At4g03190	AtAFB1
		AT4G24390.1	AtAFB4
		AT5G49980.1	AtAFB5
		AT1G12820.1	AtAFB3
		AT3G62980.1	AtTIR
		AT2G39940.1	AtCOI1
	P. trichocarpa	POPTR_0004s03400.1	PtCOI1
		POPTR_0002s10310.1	PtCOI2
		POPTR_0001s33030.1	PtCOI3
		POPTR_0020s00280.1	PtCOI4
	G. hyrsutum	Gh_A02G0685	GhFBOX258
		Gh_D02G0731	GhFBOX514
		Gh_D04G0642	GhFBOX518
		Gh_A05G2749	GhFBOX504
		Gh_D06G0276	GhFBOX523
		Gh_D05G2130	GhFBOX522
		Gh_A06G1896	GhFBOX506
		Gh_A10G0207	GhFBOX439
	S. bicolor	Sb09g022040.1	SbCOI1
		Sb03g040150.1	SbCOI2
		Sb01g040110.1	SbCOI3
		Sb04g033850.1	SbCOI4
		Sb06g014420.1	SbCOI5
		Sb05g018860.1	SbCOI6
		Sb01g044720.1	SbCOI7
		Sb09g003870.1	SbCOI8
	O. sativa	LOC_Os01g63420.1	OsCOI1
		LOC_Os05g37690.1	OsCOI2
		LOC_Os03g15880.1	OsCOI3

Table S5. Genes of repressor proteins DELLA, JAZ, and IAA organized by species. The table includes CodeID and the corresponding names assigned to the phylogenetic tree leaves.

Family	Species	Code ID	Name
DELLA	*A. thaliana*	AT1G14920	AtGAI
		AT2G01570	AtRGA
		AT1G66350	AtRGL1
		AT3G03450	AtRGL2
		AT5G17490	AtRGL3
	G. hyrsutum	Gh_A01G1242	GhDELLA1
		Gh_A07G0717	GhDELLA2
		Gh_D01G1446	GhDELLA3
	S. lycopersicum	solyc10g086370.1.1	SlDELLA1
		solyc10g086380.1.1	SlDELLA2
		solyc11g011260.1.1	SlDELLA3/PROCERA
	O. sativa	LOC_Os01g45860	OsGRAS1
		LOC_Os03g49990.1	OsSLR1
		LOC_Os11g31100.1	OsGRAS43
		LOC_Os01g71970.1	OsGRAS5
		LOC_Os05g49930.1	OsGRAS27
		LOC_Os01g67650.1	OsGRAS4
	Z. mays	GRMZM2G1594757	ZmGRAS58
		GRMZM2G342217	ZmGRAS68
		GRMZM5G874545	ZmGRAS83
		GRMZM2G144744	ZmGRAS54
		GRMZM2G024973	ZmGRAS12
		GRMZM5G826526	ZmGRAS81
		GRMZM2G001426	ZmGRAS5
		GRMZM2G023872	ZmGRAS11
	B. distachyon	BRADI1G32070	BdDELLA1
		BRADI1G47900	BdDELLA2
		BRADI2G45117	BdDELLA3
		BRADI4G18390	BdDELLA4
		BRADI1G11090	BdDELLA5
		BRADI2G57940	BdDELLA6
JAZ	*A. thaliana*	AT1G19180	AtJAZ1
		AT1G74950	AtJAZ2
		AT3G17860	AtJAZ3
		AT1G48500	AtJAZ4
		AT1G17380	AtJAZ5
		AT1G72450	AtJAZ6
		AT2G34600	AtJAZ7
		AT1G30135	AtJAZ8
		AT1G70700	AtJAZ9
		AT5G13220	AtJAZ10
		AT3G43440	AtJAZ11
		AT5G20900	AtJAZ12
		AT3G22275	AtJAZ13
	P. trichocarpa	Potri.012G044900	PtJAZ11
		Potri.015G035800	PtJAZ12
		Potri.018G047100	PtJAZ13
		Potri.001G062500	PtJAZ2
		Potri.003G165000	PtJAZ6
		Potri.001G166200	PtJAZ3
		Potri.003G068900	PtJAZ4
		Potri.006G217200	PtJAZ7
		Potri.010G108200	PtJAZ9
		Potri.008G133400	PtJAZ8
		Potri.003G165000	PtJAZ5
		Potri.011G083900	PtJAZ10
	S. bicolor	SB01G023300	SbJAZ1
		SB01G023290	SbJAZ2

Table S5 (continuation)

JAZ	*S. bicolor*	SB01G023330	SbJAZ4
		SB01G023431	SbJAZ5
		SB01G027325	SbJAZ6
		SB01G033020	SbJAZ7
		SB01G045180	SbJAZ8
		SB01G045190	SbJAZ9
		SB02G003130	SbJAZ10
		SB02G024270	SbJAZ11
		SB02G025720	SbJAZ12
		SB02G039190	SbJAZ13
		SB03G044485	SbJAZ14
		SB06G031060	SbJAZ15
	O. sativa	LOC_Os03g08310.1	OsJAZ1
		LOC_Os03g08320.1	OsJAZ2
		LOC_Os03g08330.1	OsJAZ3
		LOC_Os03g27900.1	OsJAZ4
		LOC_Os03g28940.1	OsJAZ5
		LOC_Os04g32480.1	OsJAZ6
		LOC_Os04g55920.2	OsJAZ7
		LOC_Os07g05830.1	OsJAZ8
		LOC_Os07g42370.1	OsJAZ9
		LOC_Os08g33160.1	OsJAZ10
		LOC_Os09g23660.1	OsJAZ11
		LOC_Os09g26780.1	OsJAZ12
		LOC_Os10g25230.1	OsJAZ13
		LOC_Os10g25250.1	OsJAZ14
		LOC_Os10g25290.1	OsJAZ15
	P. patens	Pp1s15_168V6.1	PpJAZ1
		Pp1s88_112V6.1	PpJAZ2
		Pp1s88_114V6.1	PpJAZ3
		Pp1s103_27V6.1	PpJAZ4
		Pp1s103_29V6.2	PpJAZ5
		Pp1s442_10V6.1	PpJAZ6
		Pp1s442_14V6.1	PpJAZ7
IAA	*A. thaliana*	AT4G14560.1	AtIAA1
		AT3G23030.1	AtIAA2
		AT1G04240.1	AtIAA3
		AT5G43700.1	AtIAA4
		AT1G15580.1	AtIAA5
		AT1G52830.1	AtIAA6
		AT3G23050.1	AtIAA7
		AT2G22670.1	AtIAA8
		AT5G65670.1	AtIAA9
		AT1G04100.1	AtIAA10
		AT4G28640.1	AtIAA11
		AT1G04550.1	AtIAA12
		AT2G33310.1	AtIAA13
		AT4G14550.1	AtIAA14
		AT1G80390.1	AtIAA15
		AT3G04730.1	AtIAA16
		AT1G04250.1	AtIAA17
		AT1G51950.1	AtIAA18
		AT3G15540.1	AtIAA19
		AT2G46990.1	AtIAA20
		AT3G16500.1	AtIAA26
		AT4G29080.1	AtIAA27
		AT5G25890.1	AtIAA28
		AT4G32280.1	AtIAA29
		AT3G62100.1	AtIAA30
		AT3G17600.1	AtIAA31
		AT2G01200.1	AtIAA32
		AT5G57420.1	AtIAA33
		AT1G15050.1	AtIAA34

Table S5 (continuation)

IAA	*O. sativa*	LOC_Os01g08200	OsIAA1
		LOC_Os01g09450	OsIAA2
		LOC_Os01g13030	OsIAA3
		LOC_Os01g18360	OsIAA4
		LOC_Os01g48450	OsIAA5
		LOC_Os01g53880	OsIAA6
		LOC_Os02g13520	OsIAA7
		LOC_Os02g49160	OsIAA8
		LOC_Os02g56120	OsIAA9
		LOC_Os03g57250	OsIAA10
		LOC_Os03g43400	OsIAA11
		LOC_Os03g43410	OsIAA12
		LOC_Os03g53150	OsIAA13
		LOC_Os05g58350	OsIAA14
		LOC_Os05g08570	OsIAA15
		LOC_Os05g09480	OsIAA16
		LOC_Os05g14180	OsIAA17
		LOC_Os05g44810	OsIAA18
		LOC_Os05g48590	OsIAA19
		LOC_Os06g07040	OsIAA20
		LOC_Os06g22870	OsIAA21
		LOC_Os06g24850	OsIAA22
		LOC_Os06g39590	OsIAA23
		LOC_Os07g08460	OsIAA24
		LOC_Os08g01780	OsIAA25
		LOC_Os09g35870	OsIAA26
		LOC_Os11g11410	OsIAA27
		LOC_Os11g11420	OsIAA28
		LOC_Os11g11430	OsIAA29
		LOC_Os12g40890	OsIAA30
	S. tuberosum	PGSC0003DMC400028515	StIAA1
		PGSC0003DMC400034949	StIAA2
		PGSC0003DMC400033533	StIAA3
		PGSC0003DMC400011339	StIAA4
		PGSC0003DMC400051083	StIAA5
		PGSC0003DMC400004537	StIAA6
		PGSC0003DMC400028445	StIAA7
		PGSC0003DMC400004724	StIAA8
		PGSC0003DMC400033829	StIAA9
		PGSC0003DMC400009432	StIAA10
		PGSC0003DMC400004726	StIAA11
		PGSC0003DMC400023793	StIAA12
		PGSC0003DMC400010270	StIAA13
		PGSC0003DMC400004659	StIAA14
		PGSC0003DMC400000263	StIAA15
		PGSC0003DMC400028876	StIAA16
		PGSC0003DMC400009456	StIAA17
		PGSC0003DMC400035574	StIAA18
		PGSC0003DMC400053819	StIAA19
		PGSC0003DMC400065246	StIAA20
		PGSC0003DMC400024324	StIAA21
		PGSC0003DMC400015088	StIAA22
		PGSC0003DMC400010843	StIAA23

Table S5 (continuation)

	S. tuberosum	PGSC0003DMC400010816	StIAA24
		PGSC0003DMC400002698	StIAA25
		PGSC0003DMC400000747	StIAA26
		PGSC0003DMC400037380	StIAA27
IAA	*M. truncatula*	Medtr1g040675	MtIAA1
		Medtr1g069495	MtIAA2
		Medtr1g070520	MtIAA3
		Medtr1g070830	MtIAA4
		Medtr1g080860	MtIAA5
		Medtr1g085750	MtIAA6
		Medtr1g093240	MtIAA7
		Medtr1g093350	MtIAA8
		Medtr1g109510	MtIAA9
		Medtr2g100780	MtIAA10
		Medtr2g101500	MtIAA11
		Medtr2g102490	MtIAA12
		Medtr3g106850	MtIAA13
		Medtr4g011880	MtIAA14
		Medtr4g115075	MtIAA15
		Medtr4g124300	MtIAA16
		Medtr4g128070	MtIAA17
		Medtr5g030710	MtIAA18
		Medtr5g067350	MtIAA19
		Medtr6g488150	MtIAA20
		Medtr7g096090	MtIAA21
		Medtr7g110790	MtIAA22
		Medtr8g014520	MtIAA23
		Medtr8g067530	MtIAA24
		Medtr8g103030	MtIAA25

Table S6. Genes of transcription factors PIF, BEH, MYC, and ARF organized by species. The table includes CodeID and the corresponding names assigned to the phylogenetic tree leaves.

Family	Species	Code ID	Name
PIF	*A. thaliana*	AT1G09530	AtPIF3
		AT2G46970.1	AtPIF2
		AT3G59060.2	AtPIF5
		AT2G43010	AtPIF4
		AT3G62090	AtPIF6
		AT4G00050	AtPIF8
		AT5G61270	AtPIF7
		AT2G20180	AtPIF1
		AT4G36930	AtSPT
		AT5G67110	AtALC
	S. lycopersicum	Solyc09g063010	SlPIF1a
		Solyc06g008030	SlPIF1b
		Solyc01g102300	SlPIF3
		Solyc07g043580	SlPIF4
		Solyc03g115540	SlPIF7a
		Solyc06g069600	SlPIF7b
		Solyc04g078690	SlALC
		Solyc02g093280	SlSPT
	S. tuberosum	PGSC0003DMG400018280	StbHLH12
		PGSC0003DMG400005998	StbHLH46
		PGSC0003DMG400004011	StbHLH32
		PGSC0003DMG401007955	StbHLH54
		PGSC0003DMG400025976	StbHLH8
		PGSC0003DMG400024554	StbHLH42
		PGSC0003DMG400033087	StbHLH71
		PGSC0003DMG401015926	StbHLH78
		PGSC0003DMG400014705	StbHLH64
		PGSC0003DMG400018950	StbHLH96
	S. bicolor	SORBI_3009G036400	SbbHLH154
		SORBI_3003G136900	SbbHLH066
		SORBI_3006G217800	SbbHLH119
		SORBI_3008G163700	SbbHLH151
		SORBI_3001G068301	SbbHLH004
		SORBI_3002G031000	SbbHLH036
	O. sativa	LOC_Os03g56950	OsPIL13
		LOC_Os07g05010	OsPIL14
		Os01g0286100	OsjPIL15
BEH	*A. thaliana*	AT1G75080.1	AtBZR1
		AT1G19350.1	AtBES1
		AT3G50750.1	AtBEH1
		AT4G36780.1	AtBEH2
		AT4G18890.1	AtBEH3
		AT1G78700.1	AtBEH4
		AT2G45880.1	AtBMY4
		AT5G45300.1	AtBMY2
	S. lycopersicum	Solyc12g089040.1.1	SlBZR1
		Solyc04g079980.2.1	SlBES1
		Solyc01g094580	SlBAM7
		Solyc01g005780	SlBAM8
	M. truncatula	MTR 1g021990	MtBZR1
		MTR 2g075400	MtBZR2
		Medtr5g026210.1	MtBAM8
	N. benthamiana	A0A0F6VYB2	NbBZR1
	O. sativa	OS07G0580500	OsBZR1
		OS01G0203000	OsBZR2
		OS06G0552300	OsBZR3
		OS02G0233200	OsBZR4
		LOC_Os09g39570.1	OsBAM7
		LOC_Os02g03690.1	OsBAM8
	S. bicolor	Sb01g047720	SbBZR1

Table S6 (continuation)

MYC	*A. thaliana*	AT4G00480	AtMYC1
		AT1G32640	AtMYC2
		AT5G46760	AtMYC3
		AT4G17880	AtMYC4
		AT5G46830	AtMYC5
		AT5G41315	AtMYC6.2
		AT2G46810	AtMYC70
		AT3G61950	AtMYC67
	S. lycopersicum	Solyc01g096050.2.1	-
		Solyc01g096370.2.1	-
		Solyc05g050560.1.1	-
		Solyc06g083980.1.1	-
		Solyc08g076930.1.1	-
		Solyc08g083170.1.1	-
		Solyc10g009270.2.1	-
		Solyc10g009290.1.1	-
	P. trichocarpa	eugene3.01420061	-
		eugene3.00130068	-
	B. distachyon	Bradi1g54070	BdMYC1
		Bradi1g54111	BdMYC2
		Bradi1g59771	BdMYC3
		Bradi2g08080	BdMYC4
		Bradi2g47730	BdMYC5
		Bradi3g01910	BdMYC6
		Bradi3g34200	BdMYC7
ARF	*A. thaliana*	AT1G59750	AtARF1
		AT5G62000.1	AtARF2
		AT2G33860.1	AtARF3
		AT5G60450.1	AtARF4
		AT1G19850.1	AtARF5
		AT1G30330.2	AtARF6
		AT5G20730.1	AtARF7
		AT5G37020.1	AtARF8
		AT4G23980.1	AtARF9
		AT2G28350.1	AtARF10
		AT2G28350.1	AtARF11
		AT1G34310.1	AtARF12
		AT1G34170.3	AtARF13
		AT1G35540.1	AtARF14
		AT1G35520.1	AtARF15
		AT4G30080.1	AtARF16
		AT1G77850.1	AtARF17
		AT3G61830.1	AtARF18
		AT1G19220.1	AtARF19
		AT1G35240.1	AtARF20
		AT1G34410.1	AtARF21
		AT1G34390.1	AtARF22
		AT1G43950.1	AtARF23
	N. tabacum	SGN-U421498	NtARF1
		SGN-U467157	NtARF6
		SGN-U439808	NtARF3
	O. sativa	LOC_Os01g13520.1	OsARF1
		LOC_Os01g48060.1	OsARF2
		LOC_Os01g54990.1	OsARF3
		LOC_Os01g70270.1	OsARF4
		LOC_Os02g06910.1	OsARF6
		LOC_Os02g35140.1	OsARF7
		LOC_Os02g41800.1	OsARF8
		LOC_Os04g36054.1	OsARF9
		LOC_Os04g43910.1	OsARF10
		LOC_Os04g56850.1	OsARF11
		LOC_Os04g57610.1	OsARF12
		LOC_Os05g43920.1	OsARF14

Table S6 (continuation)

ARF	*O. sativa*	LOC_Os05g48870.1	OsARF15
		LOC_Os06g46410.1	OsARF17
		LOC_Os06g47150.1	OsARF18
		LOC_Os10g33940.1	OsARF22
		LOC_Os11g32110.1	OsARF23
		LOC_Os12g29520.1	OsARF24
		LOC_Os12g41950.1	OsARF25
		LOC_Os07g08540.1	OsARF26
	S. bicolor	Sobic.001G217300.1	SbARF1
		Sobic.002G290600.1	SbARF2
		Sobic.003G003800.1	SbARF3
		Sobic.003G251700.1	SbARF4
		Sobic.003G298600.1	SbARF5
		Sobic.003G411900.1	SbARF6
		Sobic.004G051900.1	SbARF8
		Sobic.004G178500.1	SbARF9
		Sobic.004G221400.1	SbARF10
		Sobic.005G132000.1	SbARF11
		Sobic.006G089500.1	SbARF12
		Sobic.006G149600.1	SbARF13
		Sobic.006G255300.1	SbARF14
		Sobic.006G262100.1	SbARF15
		Sobic.008G096000.1	SbARF18
		Sobic.008G169400.1	SbARF19
		Sobic.009G196900.1	SbARF20
		Sobic.009G231800.1	SbARF21
		Sobic.010G229000.1	SbARF23
		Sobic.010G236300.1	SbARF24
		Sobic.009G231800.1	SbARF26
	B. distachyon	Bradi4g01730.1	BdARF1
		Bradi1g32547.1	BdARF2
		Bradi3g04920.1	BdARF3
		Bradi5g25767.4	BdARF4
		Bradi5g25157.1	BdARF5
		Bradi3g45880.2	BdARF6
		Bradi4g17410.1	BdARF7
		Bradi5g10950.1	BdARF8
		Bradi4g07470.1	BdARF9
		Bradi2g59480.1	BdARF10
		Bradi2g16610.1	BdARF11
		Bradi2g50120.1	BdARF12
		Bradi2g46190.1	BdARF13
		Bradi2g08120.2	BdARF14
		Bradi2g19867.1	BdARF15
		Bradi3g28950.1	BdARF16
		Bradi3g49320.1	BdARF17
		Bradi5g15904.1	BdARF18
		Bradi1g33160.1	BdARF19

Table S7. Ka/Ks analysis and divergence time between the duplicated genes pairs. Ka. non-synonymous substitution rate; Ks. Synonymous substitution rate; MYA. Million years ago.

Putative Function	Gene 1	Gene 2	Type	Ka	Ks	Ka/Ks	Date (MYA)
Receptors	RcGID1b	RcGID1c	Segmental	0.183112	1.67023	0.109633	6.76E-09
Repressors	RcDELLA3	RcDELLA1	Segmental	0.235681	2.39632	0.098351	9.71E-09
	RcJAZ3	RcJAZ4	Segmental	0.508626	1.44825	0.351200	5.87E-09
	RcJAZ7	RcJAZ4	Segmental	0.753028	1.59012	0.473567	6.44E-09
	RcIAA11	RcIAA9	Segmental	0.202721	1.39150	0.145685	5.64E-09
	RcIAA2	RcIAA9	Segmental	0.444958	3.64623	0.122032	1.48E-08
	RcIAA5	RcIAA17	Segmental	0.422048	1.40372	0.300663	5.69E-09
	RcIAA19	RcIAA1	Segmental	0.203883	1.33310	0.152939	5.40E-09
	RcIAA11	RcIAA3	Segmental	0.239859	2.68565	0.089311	1.09E-08
	RcIAA12	RcIAA8	Segmental	0.135865	1.22585	0.110833	4.96E-09
	RcIAA12	RcIAA2	Segmental	0.190183	1.41196	0.134694	5.72E-09
	RcIAA13	RcIAA18	Segmental	0.203135	1.05838	0.191929	4.29E-09
	RcIAA14	RcIAA10	Segmental	0.482480	4.09041	0.117954	1.66E-08
	RcIAA4	RcIAA15	Segmental	0.671516	1.49123	0.450311	6.04E-09
Trancription factors	RcALC	RcSPT	Segmental	0.468817	1.73496	0.270217	7.03E-09
	RcMYC1	RcMYC6	Segmental	0.522050	3.07350	0.169855	1.24E-08
	RcMYC5	RcMYC4	Segmental	0,438439	2.16879	0.202158	8.78E-09
	RcMYC12	RcMYC14	Segmental	0,742969	2.06077	0.360530	8.35E-09
	RcARF11	RcARF17	Segmental	0,789336	2.82696	0.279217	1.14E-08
	RcARF14	RcARF2	Segmental	0,30425	1.54099	0.197438	6.24E-09
	RcARF14	RcARF16	Segmental	0,241159	1.38665	0.173915	5.62E-09
	RcARF2	RcARF16	Segmental	0,819391	3.29760	0.248481	1.34E-08
	RcBEH1	RcBEH2	Segmental	0,579482	3.08839	0.187633	1.25E-08

Table S8. Linked genes between *A. thaliana* and *R. communis*. The pair genes were obtained from the collinearity analysis using MCScanX.

R. communis				***A. thaliana***			
**Chr**	**Transcript code (NCBI)**	**Locus (Phytozome)**	**Gene Name**	**Chr**	**Transcript code (NCBI)**	**Locus (TAIR)**	**Gene Name**
NC_063256.1	XM_015728930.3	30174.m009029	RcMYC1	NC_003070.9	NM_001198373.2	AT1G63650	EGL3
NC_063256.1	XM_015721891.3	29844.m003175	RcARF8	NC_003071.7	NM_001036471.1	AT2G46530	ARF11
NC_063256.1	XM_002524721.4	29703.m001506	RcGID1b	NC_003074.8	NM_116166.5	AT3G63010	GID1b
NC_063256.1	XM_015721891.3	29844.m003175	RcARF8	NC_003074.8	NM_116048.3	AT3G61830	ARF18
NC_063256.1	XM_002520933.4	29883.m001992	RcIAA11	NC_003074.8	NM_001338609.1	AT3G23030	IAA2
NC_063256.1	XM_002520934.3	29883.m001993	RcIAA12	NC_003074.8	NM_113205.3	AT3G23050	IAA7
NC_063256.1	XM_002529919.4	27964.m000356	RcMYC5	NC_003075.7	NM_116313.3	AT4G00870	bHLH protein
NC_063256.1	XM_002520934.3	29883.m001993	RcIAA12	NC_003075.7	NM_001340945.1	AT4G14550	IAA14
NC_063256.1	XM_002520933.4	29883.m001992	RcIAA11	NC_003075.7	NM_117536.4	AT4G14560	IAA1
NC_063256.1	XM_015728930.3	30174.m009029	RcMYC1	NC_003075.7	NM_001160722.2	AT4G00480	MYC1
NC_063256.1	XM_015728930.3	30174.m009029	RcMYC1	NC_003076.8	NM_001344395.1	AT5G41315	MYC6.2
NC_063257.1	XM_002510301.4	30170.m013999	RcBZR1	NC_003070.9	NM_202416.1	AT1G75080	BZR1
NC_063257.1	XM_002510301.4	30170.m013999	RcBZR1	NC_003070.9	NM_202134.1	AT1G19350	BZR2/BES1
NC_063257.1	XM_002513939.4	29908.m006098	RcMYC12	NC_003070.9	NM_001332550.1	AT1G22490	bHLH protein
NC_063257.1	XM_048370944.1	30170.m013613	RcIAA19	NC_003071.7	NM_001202650.1	AT2G22670	IAA8
NC_063257.1	XM_002510301.4	30170.m013999	RcBZR1	NC_003075.7	NM_119842.7	AT4G36780	BEH2
NC_063257.1	XM_002510144.4	30170.m013852	RcALC	NC_003076.8	NM_126111.4	AT5G67110	ALC
NC_063257.1	XM_002513939.4	29908.m006098	RcMYC12	NC_003076.8	NM_125930.2	AT5G65320	bHLH protein
NC_063258.1	XM_002516197.3	29739.m003727	RcJAZ3	NC_003070.9	NM_001332386.1	AT1G19180	JAZ1
NC_063258.1	XM_002516197.3	29739.m003727	RcJAZ3	NC_003070.9	NM_105904.4	AT1G72450	JAZ6
NC_063258.1	XM_002516130.4	29739.m003659	RcJAZ7	NC_003074.8	NM_114212.5	AT3G43440	JAZ11
NC_063258.1	XM_015724189.3	29927.m000601	RcIAA14	NC_003075.7	NM_119380.3	AT4G32280	IAA29
NC_063258.1	XM_002516130.4	29739.m003659	RcJAZ7	NC_003076.8	NM_122098.5	AT5G20900	JAZ12
NC_063258.1	XM_048371544.1	29794.m003410	RcIAA5	NC_003076.8	NM_122490.4	AT5G25890	IAA28
NC_063259.1	XM_048373260.1	30147.m014418	RcARF11	NC_003076.8	NM_125593.5	AT5G62000	ARF2
NC_063260.1	XM_002533984.4	28677.m000055	RcDELLA3	NC_003070.9	NM_105306.4	AT1G66350	RGL1
NC_063260.1	XM_002533984.4	28677.m000055	RcDELLA3	NC_003071.7	NM_126218.3	AT2G01570	RGA1
NC_063262.1	XM_015723807.3	29637.m000735	RcIAA4	NC_003070.9	NM_100291.3	AT1G04100	IAA10
NC_063262.1	XM_015723807.3	29637.m000735	RcIAA4	NC_003071.7	NM_179874.2	AT2G33310	IAA13
NC_063262.1	XM_015723807.3	29637.m000735	RcIAA4	NC_003075.7	NM_001084991.1	AT4G28640	IAA11
NC_063263.1	XM_002529895.3	27538.m000327	RcARF14	NC_003071.7	NM_128394.5	AT2G28350	ARF10
NC_063264.1	XM_002516978.4	29844.m003175	RcIAA9	NC_003070.9	NM_001331470.1	AT1G04240	IAA3
NC_063264.1	XM_002516978.4	29844.m003175	RcIAA9	NC_003074.8	NM_001338609.1	AT3G23030	IAA2
NC_063264.1	XM_002517114.4	29844.m003312	RcMYC15	NC_003074.8	NM_111487.3	AT3G06120	MUTE
NC_063264.1	XM_002516978.4	29844.m003175	RcIAA9	NC_003075.7	NM_001340945.1	AT4G14550	IAA14
NC_063264.1	XM_002516978.4	29844.m003175	RcIAA9	NC_003076.8	NM_123736.3	AT5G43700	IAA4

Table S9. Conserved miRNAs targeting the genes from gibberellin signaling in *R. communis*. miRNA name; target of miRNA; expectation (Exp.); UPE$; proposed alignment; inhibition type; multiplicity.

miRNA_Acc.	Target_Acc.	Expectation	UPE$	alignment	Inhibition	Multiplicity
miR167a	RcGID1b	5	-1	::::: ::: :::::::	Cleavage	1
miR167b	RcGID1b	5	-1	::::: ::: :::::::	Cleavage	1
miR167d	RcGID1b	5	-1	::::: ::: :::::::	Cleavage	1
miR167e	RcGID1b	5	-1	::::: ::: :::::::	Cleavage	1
miR395a	RcGID1c	3.5	-1	.::::: :.:::::::: .:	Cleavage	1
miR395b	RcGID1c	3.5	-1	.::::: :.:::::::: .:	Cleavage	1
miR395c	RcGID1c	3.5	-1	.::::: :.:::::::: .:	Cleavage	1
miR395d	RcGID1c	3.5	-1	.::::: :.:::::::: .:	Cleavage	1
miR395e	RcGID1c	3.5	-1	.::::: :.:::::::: .:	Cleavage	1
miR167a	RcDELLA1	5	-1	: ::::.:: .:::::.:	Cleavage	1
miR167b	RcDELLA1	5	-1	: ::::.:: .:::::.:	Cleavage	1
miR167c	RcDELLA1	4.5	-1	: ::::.:: .:::::::	Cleavage	1
miR167d	RcDELLA1	5	-1	: ::::.:: .:::::.:	Cleavage	1
miR167e	RcDELLA1	5	-1	: ::::.:: .:::::.:	Cleavage	1
miR172a	RcDELLA3	5	-1	:: :: :::: ::.::::::	Translation	1
miR172b	RcDELLA3	5	-1	:: :: :::: ::.::::::	Translation	1
miR172c	RcDELLA3	5	-1	:: :: :::: ::.::::::	Translation	1
miR156c	RcSNE	5	-1	.:::::.:.:: :::..:	Cleavage	1
miR156e	RcSNE	5	-1	.:::::.:.:: :::..:	Cleavage	1
miR156g	RcSNE	5	-1	.:::::.:.:: :::..:	Cleavage	1
miR159c	RcPIF1	5	-1	: ::::: .:: .:::::::	Cleavage	1
miR168a	RcPIF2	5	-1	.::::: :.:::::: ::	Cleavage	1
miR395a	RcBMY2	5	-1	: : :..: :::.:::::::	Cleavage	1
miR395b	RcBMY2	5	-1	: : :..: :::.:::::::	Cleavage	1
miR395c	RcBMY2	5	-1	: : :..: :::.:::::::	Cleavage	1
miR395d	RcBMY2	5	-1	: : :..: :::.:::::::	Cleavage	1
miR395e	RcBMY2	5	-1	: : :..: :::.:::::::	Cleavage	1

Table S10. Conserved miRNAs targeting the genes from jasmonate signaling in *R. communis*. miRNA name; target of miRNA; expectation (Exp.); UPE$; proposed alignment; inhibition type; multiplicity.

miRNA_Acc.	Target_Acc.	Expectation	UPE$	alignment	Inhibition	Multiplicity
miR164a	RcCOI1	4	-1	: :::::: :.:::::.: ::	Cleavage	1
miR164b	RcCOI1	5	-1	:: ::: :.:::::.: ::	Cleavage	1
miR164c	RcCOI1	4	-1	: :::::: :.:::::.: ::	Cleavage	1
miR164d	RcCOI1	4	-1	: :::::: :.:::::.: ::	Cleavage	1
miR159a	RcCOI1	5	-1	: :: ::.:::: :..:::	Cleavage	1
miR164a	RcAFB1	5	-1	:::: :.:::.::::. :	Cleavage	1
miR393b	RcAFB1	5	-1	::: :::.::::::::	Cleavage	1
miR164c	RcAFB1	5	-1	:::: :.:::.::::. :	Cleavage	1
miR164d	RcAFB1	5	-1	:::: :.:::.::::. :	Cleavage	1
miR156d	RcJAZ2	5	-1	::.:.:.:::: :.:: :::	Cleavage	1
miR6445	RcJAZ4	5	-1	:::. :::::::: ::	Cleavage	1
miRN3	RcJAZ7	5	-1	:: .:::.::::::::: :	Cleavage	1
miR169a	RcJAZ8	5	-1	: ::: :.::::::::	Cleavage	1
miR169b	RcJAZ8	5	-1	: ::: :.::::::::	Cleavage	1
miR172b	RcJAZ8	4.5	-1	:. : ::..::::::::	Cleavage	1
miR172c	RcJAZ8	4.5	-1	:. : ::..::::::::	Cleavage	1
miR156d	RcJAZ9	5	-1	:.:::.:.::::: ::.	Cleavage	1
miR171e	RcMYC1	5	-1	:::::.:::: :::.::.:.:	Translation	1
miR396a	RcMYC2	4.5	-1	.:::: .:::::: ::::::	Cleavage	1
miR396b	RcMYC2	4.5	-1	:::: .:::::: ::::::	Cleavage	1
miR396c	RcMYC2	4.5	-1	:::: .:::::: ::::::	Cleavage	1
miR395a	RcMYC5	5	-1	::: .:::::::.. :::	Cleavage	1
miR395b	RcMYC5	5	-1	::: .:::::::.. :::	Cleavage	1
miR395c	RcMYC5	5	-1	::: .:::::::.. :::	Cleavage	1
miR395d	RcMYC5	5	-1	::: .:::::::.. :::	Cleavage	1
miR395e	RcMYC5	5	-1	::: .:::::::.. :::	Cleavage	1
miR482b	RcMYC5	5	-1	: :::::. ::..:::.:::	Cleavage	1
miR171a	RcMYC7	5	-1	.: ::: :.::.:::.:::	Cleavage	1
miR171b	RcMYC7	5	-1	.: ::: :.::.:::.:::	Cleavage	1
miR171d	RcMYC7	5	-1	.: ::: :.::.:::.:::	Cleavage	1
miR171f	RcMYC7	5	-1	.: ::: :.::.:::.:::	Cleavage	1
miR171g	RcMYC7	5	-1	.: ::: :.::.:::.:::	Cleavage	1
miR171h	RcMYC7	5	-1	.: ::: :.::.:::.:::	Cleavage	1
miR395a	RcMYC7	5	-1	:: : ::::::..:::::	Cleavage	1
miR395b	RcMYC7	5	-1	:: : ::::::..:::::	Cleavage	1
miR395c	RcMYC7	5	-1	:: : ::::::..:::::	Cleavage	1
miR395d	RcMYC7	5	-1	:: : ::::::..:::::	Cleavage	1
miR395e	RcMYC7	5	-1	:: : ::::::..:::::	Cleavage	1
miR156j	RcMYC9	3	-1	:: :: :::::::::::	Cleavage	1
miR168b	RcMYC9	5	-1	.::.:.: :::::::.:.	Translation	1
miR168c	RcMYC9	5	-1	.::.:.: :::::::.:.	Translation	1
miR395a	RcMYC10	4	-1	::::..::::..:::::	Cleavage	1
miR395b	RcMYC10	4	-1	::::..::::..:::::	Cleavage	1
miR395c	RcMYC10	4	-1	::::..::::..:::::	Cleavage	1
miR395d	RcMYC10	4	-1	::::..::::..:::::	Cleavage	1
miR395e	RcMYC10	4	-1	::::..::::..:::::	Cleavage	1
miR156a	RcMYC14	5	-1	: ::. .::::::.: :::	Cleavage	1
miR156b	RcMYC14	4	-1	: ::.:.::::::.: :::	Cleavage	1
miR156f	RcMYC14	5	-1	: ::. .::::::.: :::	Cleavage	1
miR156h	RcMYC14	5	-1	: ::. .::::::.: :::	Cleavage	1
miR156i	RcMYC14	5	-1	: ::. .::::::.: :::	Cleavage	1

Table S11. Conserved miRNAs targeting the genes from auxin signaling in *R. communis*. miRNA name; target of miRNA; expectation; UPE$; proposed alignment; inhibition type; multiplicity.

miRNA_Acc.0	Target_Acc.	Expectation	UPE$	alignment	Inhibition	Multiplicity
miR319	RcTIR1.1	5	-1	:.::::: ::::::::.	Translation	1
miR393b	RcTIR1	5	-1	:: :: .::::: :::::	Cleavage	2
miR393b	RcTIR1	2	-1	::: :::::::::::::::::::	Cleavage	2
miR393b	RcAFB2	1	-1	: :::::::::::::::::::	Cleavage	1
miR166a	RcIAA4	5	-1	:::: :::: .:: ::.::	Translation	1
miR166b	RcIAA4	5	-1	:::: :::: .:: ::.::	Translation	1
miR166c	RcIAA4	5	-1	:::: :::: .:: ::.::	Translation	1
miR166d	RcIAA4	5	-1	:::: :::: .:: ::.::	Translation	1
miR166e	RcIAA4	5	-1	:::: :::: .:: ::.::	Translation	1
miR167a	RcIAA4	5	-1	: :.: .:: :::..:.::::	Cleavage	1
miR167b	RcIAA4	5	-1	: :.: .:: :::..:.::::	Cleavage	1
miR167d	RcIAA4	5	-1	:.: .:: :::..:.::::	Cleavage	1
miR167e	RcIAA4	5	-1	:.: .:: :::..:.::::	Cleavage	1
miR168b	RcIAA8	5	-1	: .. :::.::::::::: :	Cleavage	1
miR168c	RcIAA8	5	-1	: .. :::.::::::::: :	Cleavage	1
miR156b	RcIAA10	5	-1	:::::..::::.: .:::.	Cleavage	1
miR168b	RcIAA12	4.5	-1	:. :::.::::::::: :	Cleavage	1
miR168c	RcIAA12	4.5	-1	:. :::.::::::::: :	Cleavage	1
miR159a	RcIAA13	4.5	-1	:::: :.:::::::: :::	Cleavage	1
miR393b	RcIAA13	5	-1	:.::::::.:.:.:. ::::	Cleavage	1
miR393a	RcIAA15	5	-1	:::: .:::.::: :: ::	Cleavage	1
miR156b	RcIAA15	5	-1	::.:.:.:::.:::: :.	Cleavage	1
miR156c	RcIAA16	4	-1	::.::: ::::::: :::::	Cleavage	1
miR156e	RcIAA16	4	-1	::.::: ::::::: :::::	Cleavage	1
miR156g	RcIAA16	4	-1	::.::: ::::::: :::::	Cleavage	1
miR168a	RcIAA17	4.5	-1	: :.: .:. :.:::::::::	Cleavage	1
miR160a	RcARF1	0	-1	::::::::::::::::::::	Cleavage	1
miR160b	RcARF1	1	-1	:::: ::::::::::::::	Cleavage	1
miR160c	RcARF1	0	-1	::::::::::::::::::::	Cleavage	1
miR6445	RcARF1	5	-1	: :: : ::.::::::::.	Cleavage	1
miRN4	RcARF1	5	-1	: ::: :::: ::: ::::	Cleavage	1
miR160a	RcARF2	0	-1	:::::::::::::::::::::	Cleavage	1
miR160b	RcARF2	1	-1	::::: ::::::::::::::	Cleavage	1
miR160c	RcARF2	0	-1	:::::::::::::::::::::	Cleavage	1
miRN4	RcARF2	4.5	-1	:: : : :::: ::::::::	Cleavage	1
miR156j	RcARF4	4.5	-1	:.::::: :: : :::::::.	Translation	1
miR166f	RcARF4	5	-1	: ::::::: :::::. ::::	Cleavage	1
miR168a	RcARF4	4	-1	:::::.::::..::::: :	Cleavage	2
miR168a	RcARF4	4	-1	::::::::: :::::: :	Translation	2
miR168a	RcARF5	4	-1	:::::::::::..: ::: ::	Cleavage	1
miR394a	RcARF6	5	-1	:: : ::::: :::::...	Cleavage	1
miR394b	RcARF6	5	-1	:: : ::::: :::::...	Cleavage	1
miR156j	RcARF7	5	-1	.::..: ::::::: ::.:	Cleavage	1
miR390a	RcARF7	4	-1	: :: : ::::.:::::.::	Cleavage	1
miR390b	RcARF7	4	-1	: :: : ::::.:::::.::	Cleavage	1
miR171c	RcARF8	4.5	-1	:.:: ::::: ::::::.	Cleavage	1
miR319	RcARF8	5	-1	:.: .::..:..::::::	Cleavage	1
miRN5	RcARF8	5	-1	: :::::: :::::..: :	Translation	1
miR167a	RcARF9	3.5	-1	::::::: :::::::::::	Cleavage	1
miR167b	RcARF9	3.5	-1	::::::: :::::::::::	Cleavage	1
miR167c	RcARF9	3.5	-1	::::::: :::::::::::	Cleavage	1

Table S11 (continuation)

miR167d	RcARF9	3.5	-1	.:::::: :::::::::::	Cleavage	1
miR167e	RcARF9	3.5	-1	.:::::: :::::::::::	Cleavage	1
miR166f	RcARF10	5	-1	.: :::::::: :.: :::	Cleavage	1
miR160a	RcARF11	0.5	-1	::::::.::::::::::::::	Cleavage	1
miR160b	RcARF11	1	-1	:::::: :::::::::::::	Cleavage	1
miR160c	RcARF11	0.5	-1	::::::.::::::::::::::	Cleavage	1
miR394a	RcARF11	5	-1	: :: : .::::::::::	Cleavage	1
miR394b	RcARF11	5	-1	: :: : .::::::::::	Cleavage	1
miR160a	RcARF13	0.5	-1	::::::.::::::::::::::	Cleavage	1
miR160c	RcARF13	0.5	-1	::::::.::::::::::::::	Cleavage	1
miR164a	RcARF14	5	-1	:: :::..:::::: .::	Cleavage	1
miR164c	RcARF14	5	-1	:: :::..:::::: .::	Cleavage	1
miR164d	RcARF14	5	-1	:: :::..:::::: .::	Cleavage	1
miR168a	RcARF14	4.5	-1	:..:::: ::.:.::::::	Cleavage	1
miR477b	RcARF14	5	-1	: :::::... ...::::.::	Translation	1
miR6445	RcARF16	3.5	-1	:::::. :.:::: ::::::	Cleavage	1
miR477a	RcARF17	4.5	-1	::: .::::::. :::::	Cleavage	1
miR395a	RcARF18	5	-1	.::::.:.::: ..: ::::	Cleavage	1
miR395b	RcARF18	5	-1	.::::.:.::: ..: ::::	Cleavage	1
miR395c	RcARF18	5	-1	.::::.:.::: ..: ::::	Cleavage	1
miR395d	RcARF18	5	-1	.::::.:.::: ..: ::::	Cleavage	1
miR395e	RcARF18	5	-1	.::::.:.::: ..: ::::	Cleavage	1
miR171c	RcARF19	4.5	-1	:.:: ::::: ::::::.	Cleavage	1
miR319	RcARF19	5	-1	:.: .::..:..::::::	Cleavage	1
miRN5	RcARF19	5	-1	: :::::: :::::..: :	Translation	1
miR166f	RcARF20	5	-1	.: :::::::: :.: :::	Cleavage	1

Table S12 . Primers used to qPCR reactions. FW, Primer forward; RV, Primer reverse

Gene	Code ID	Primer	Sequence (5'-3')
RcGID1b	29703.m001506	FW	GCCTCATGGAGGAGATTAGA
		RV	TGCCTGTAGGTAAGGTTAAGT
RcGID1c	30128.m008695	FW	GGTCATGGATGAGATAAGTGA
		RV	ATGTATATCTGCCAGTCGGTA
RcCOI1	29610.m000411	FW	TCCAGATTCTGTTGTCCCCT
		RV	GACGCAACCAGCATCATGTA
RcTIR1	29647.m002022	FW	TTGCTGGGCCTCGGTTTGA
		RV	TTCATGAAAACCTCAATGCAGA
RcDELLA3	28677.m000055	FW	CTTGGCAACTCAGCGATTCA
		RV	TCATCGATTTGATAACATGAACT
RcJAZ7	29739.m003659	FW	GAAATTCAACGAGAGGTTGCT
		RV	ATATGAGAGTTTCAATTCTTCAT
RcIAA6	29841.m002748	PFW	AGAGATCGGAGGCCAAATGT
		PRV	AGTTACGATCCTCGTTTGTGA
RcPIF3	30128.m008988	PFW	ATGGGATGGACACTGAATGAA
		PRV	GGATTCTAACTGTCCTGAAAT
RcMYC2	29827.m002528	PFW	CAGGTTAGCCTTATCAACCAA
		PRV	TTTCTTCAAGCTCAACCCCAA
RcBEH1	30170.m013999	PFW	GCTCACACTTGGAAATGGGA
		PRV	AGCACTTCCTCGCAAAGATTT
RcARF7	29739.m003659	PFW	CCGCCAAAAGGAATTGGCAA
		PRV	AAATTGACTTGAGCAAGCGTAA

Figure S1

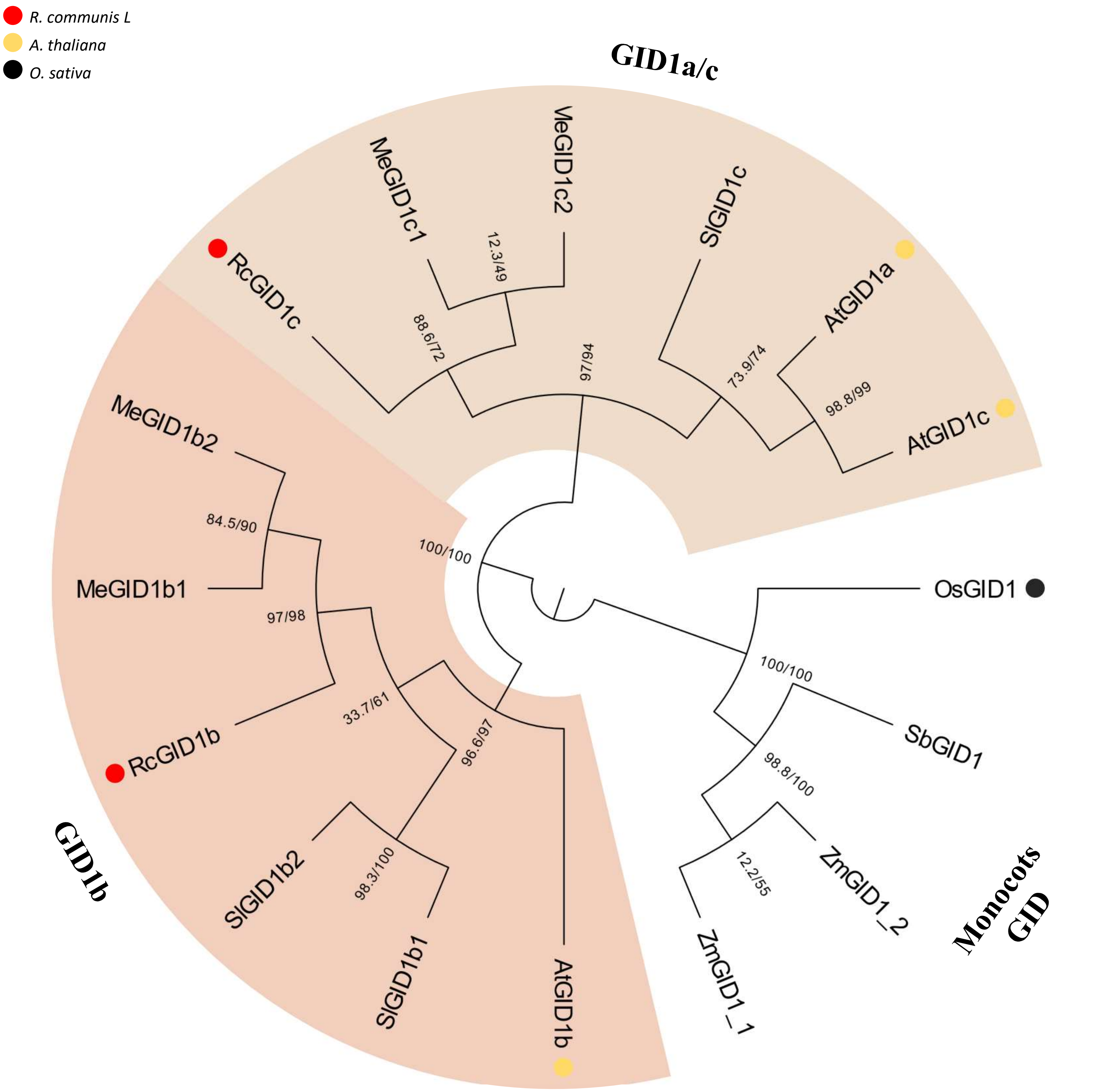

Figure S1. Maximum-likelihood phylogenetic analysis of GID1 gene family. The phylogenetic relationships among analyzed proteins were reconstructed using the maximum likelihood method under the best model selection in Iqtree software (version 1.6.12) with 1000 replicates of bootstrap and ALRT statistics. Protein sequences from *R. communis, A. thaliana and O. sativa* are highlighted by circle red, yellow and black respectively. Sequences from *S. lycopersicum, M. esculenta, Z. mays*, and *S. bicolor* were used to improve the analysis.

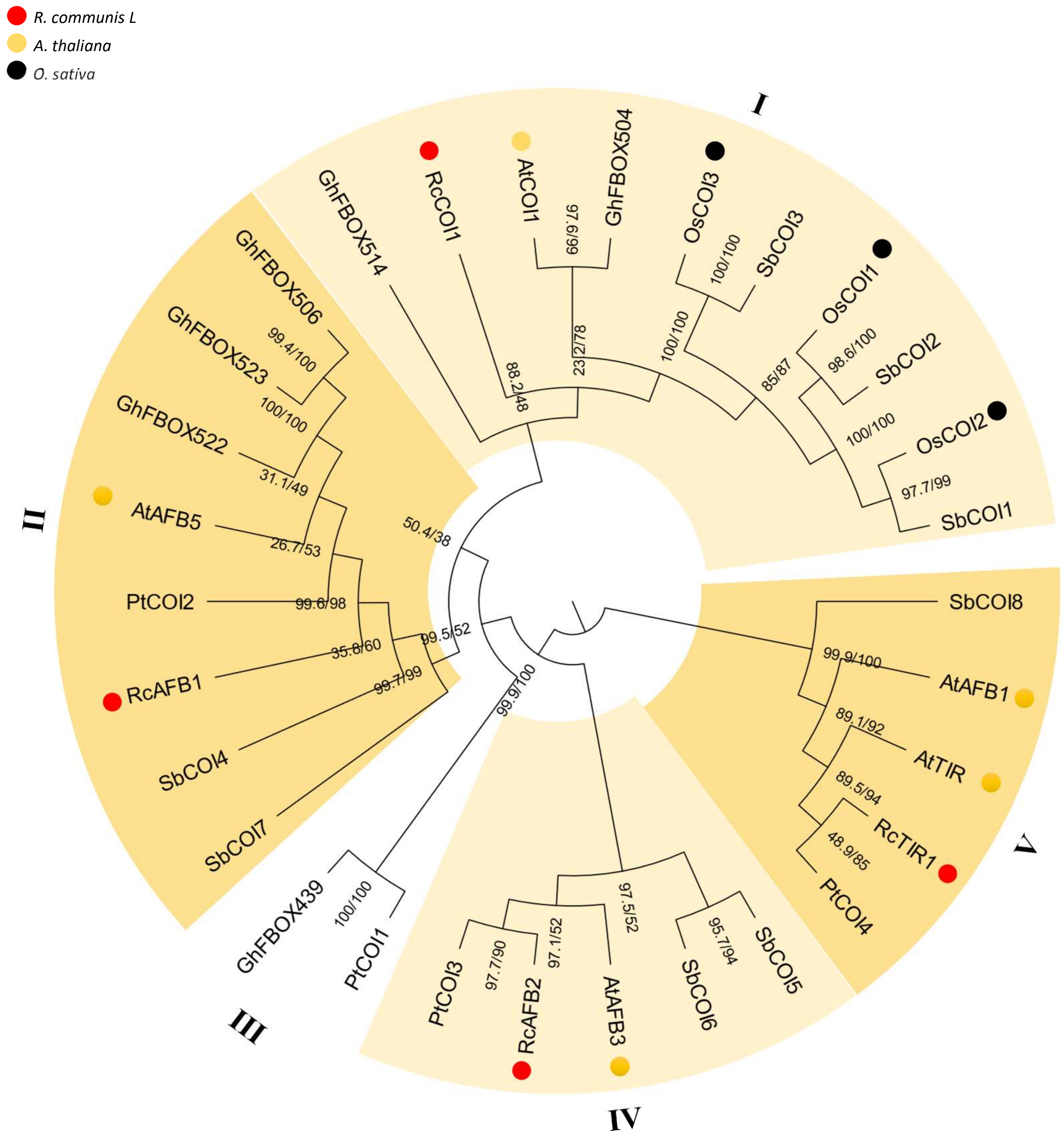

Figure S2. Maximum-likelihood phylogenetic analysis of COI1/TIR1 gene family. The phylogenetic relationships among analyzed proteins were reconstructed using the maximum likelihood method under the best model selection in Iqtree software (version 1.6.12) with 1000 replicates of bootstrap and ALRT statistics. Protein sequences from *R. communis, A. thaliana and O. sativa* are highlighted by circle red, yellow and black respectively. Sequences from *P. trichocarpa, G. hirsutum* and *S. bicolor* were used to improve the analysis.

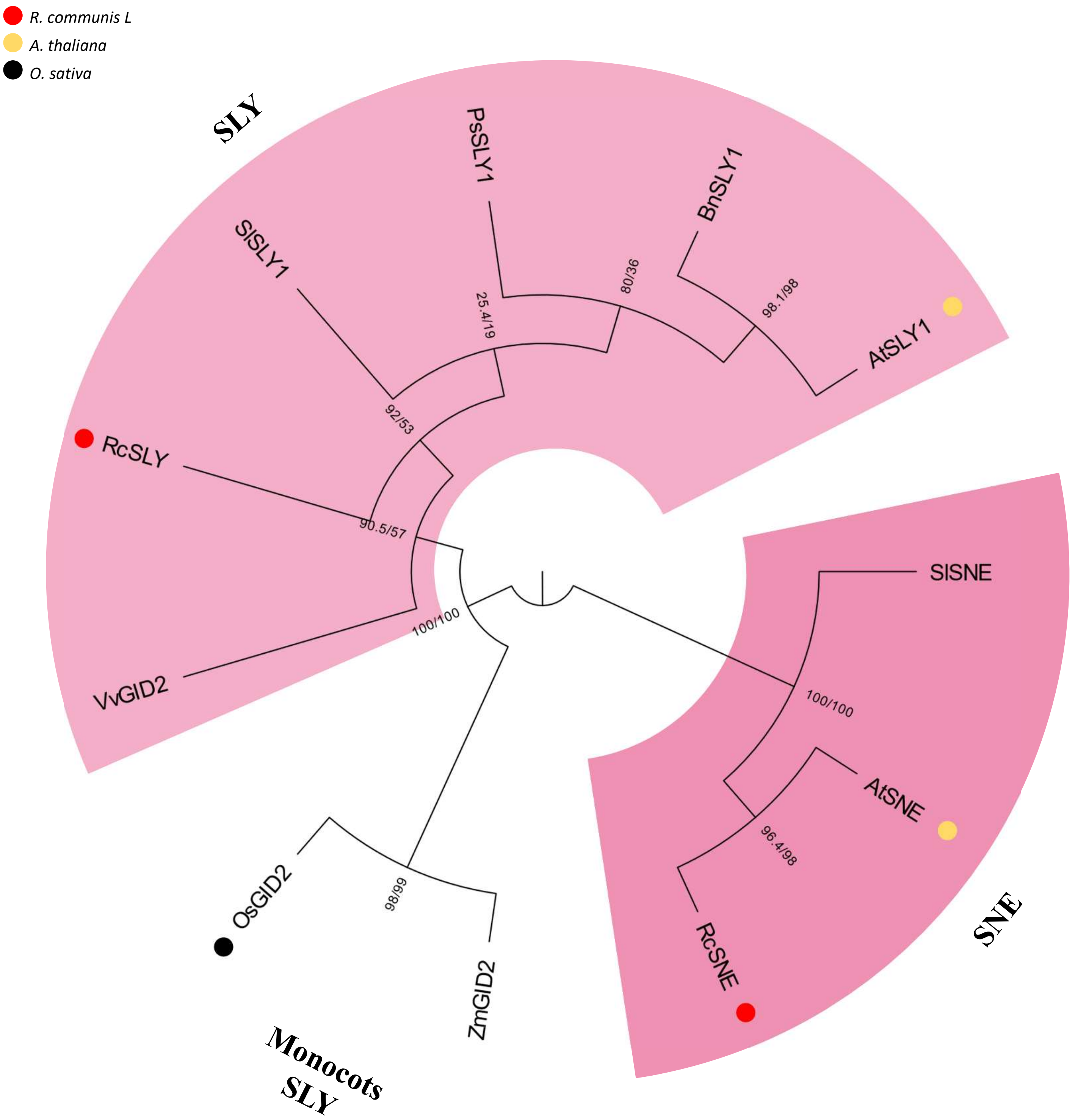

Figure S3. Maximum-likelihood phylogenetic analysis of SLY/SNE gene family. The phylogenetic relationships among analyzed proteins were reconstructed using the maximum likelihood method under the best model selection in Iqtree software (version 1.6.12) with 1000 replicates of bootstrap and ALRT statistics. Protein sequences from *R. communis, A. thaliana and O. sativa* are highlighted by circle red, yellow and black respectively. Sequences from *B. napus, S. lycopersicum, V. vinifera, Z. mays* and *P. salicina* were used to improve the analysis.

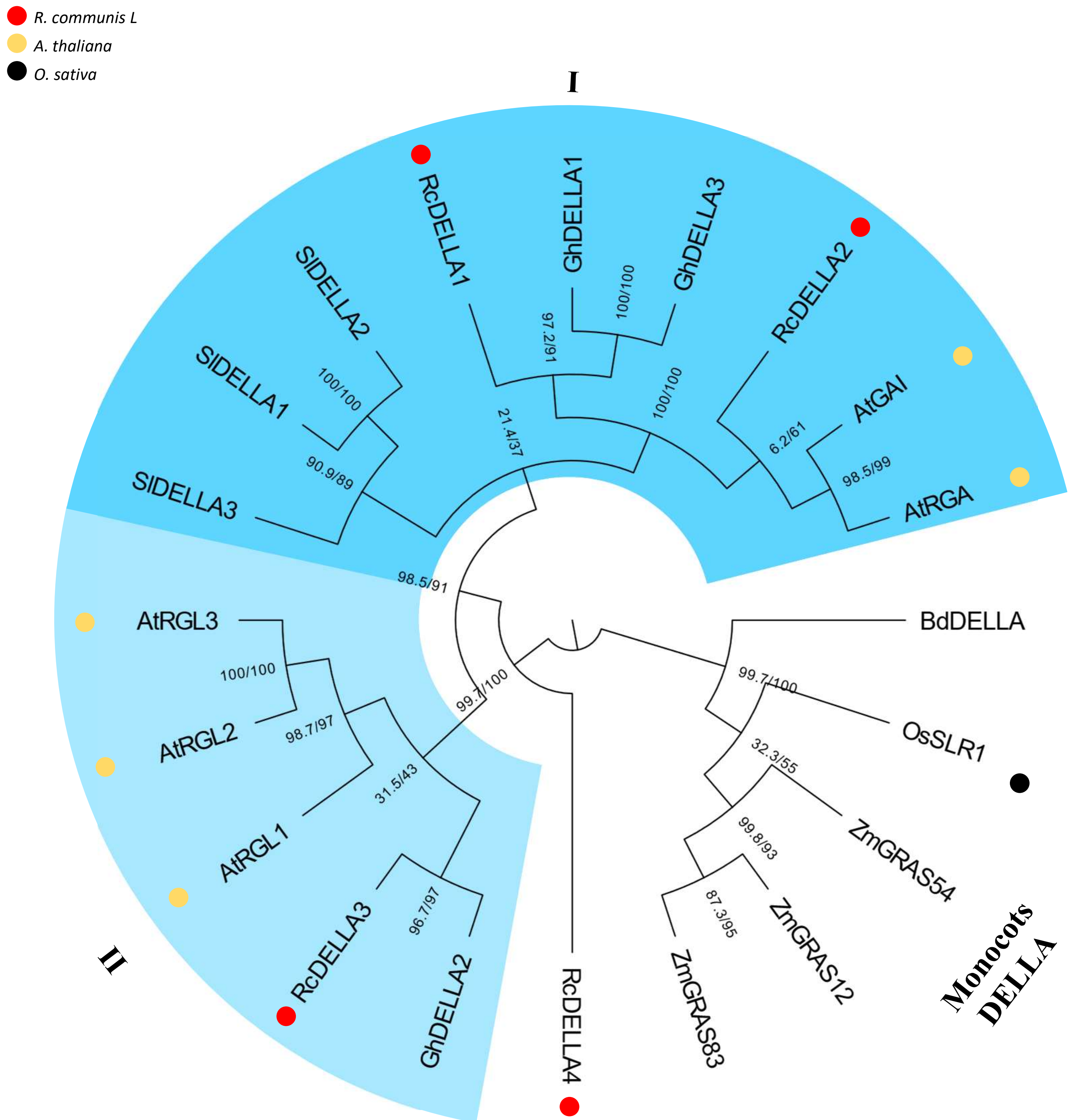

Figure S4. Maximum-likelihood phylogenetic analysis of DELLA gene family. The phylogenetic relationships among analyzed proteins were reconstructed using the maximum likelihood method under the best model selection in Iqtree software (version 1.6.12) with 1000 replicates of bootstrap and ALRT statistics. Protein sequences from *R. communis, A. thaliana and O. sativa* are highlighted by circle red, yellow and black respectively. Sequences from *S. bicolor S. lycopersicum, G. hirsutum, Z. mays,* and *B. distachyon.* were used to improve the analysis.

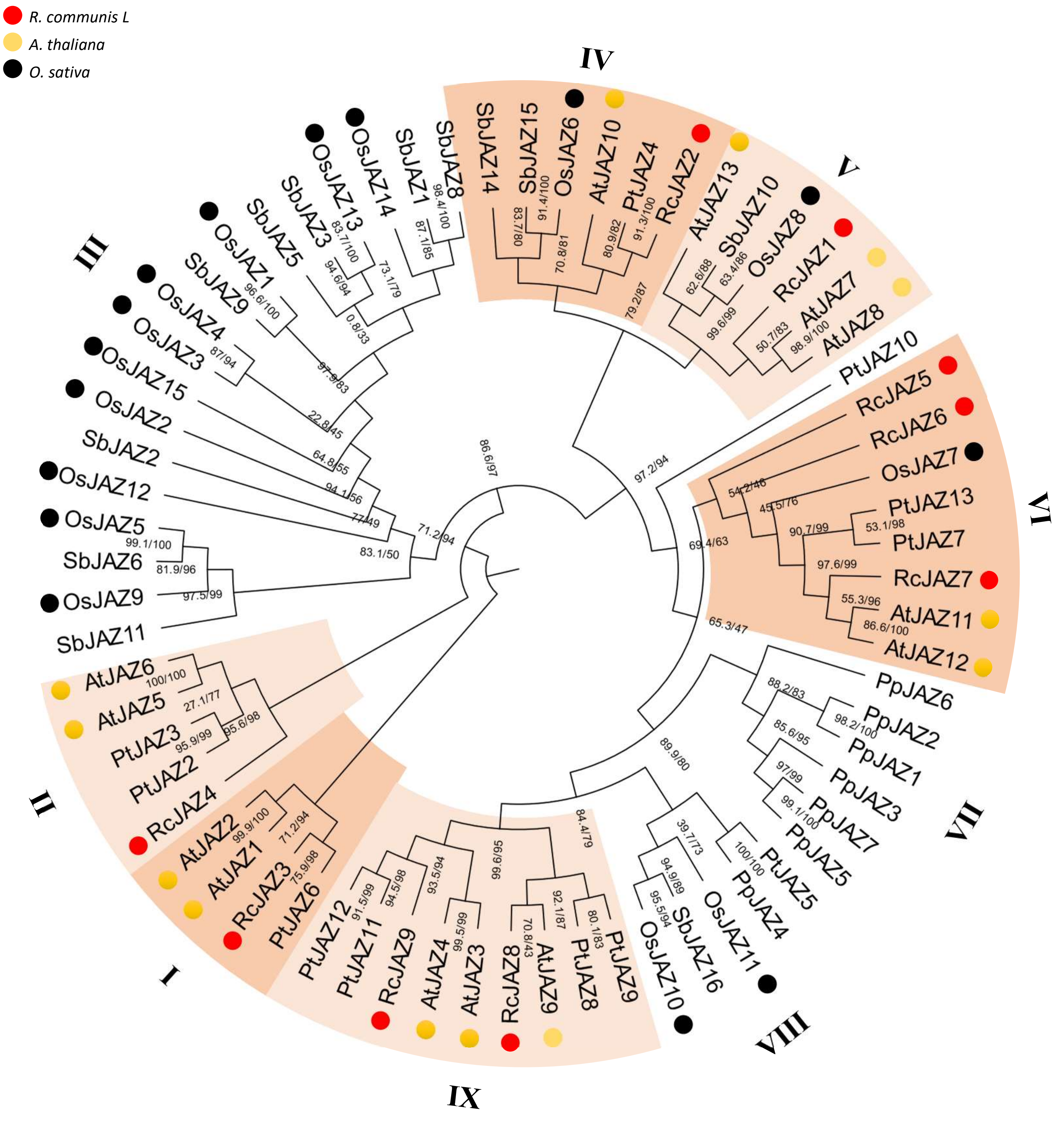

Figure S5. Maximum-likelihood phylogenetic analysis of JAZ gene family. The phylogenetic relationships among analyzed proteins were reconstructed using the maximum likelihood method under the best model selection in Iqtree software (version 1.6.12) with 1000 replicates of bootstrap and ALRT statistics. Protein sequences from *R. communis, A. thaliana and O. sativa* are highlighted by circle red, yellow and black respectively. Sequences from *P. trichocarpa, S. bicolor,* and *P. parttens* were used to improve the analysis.

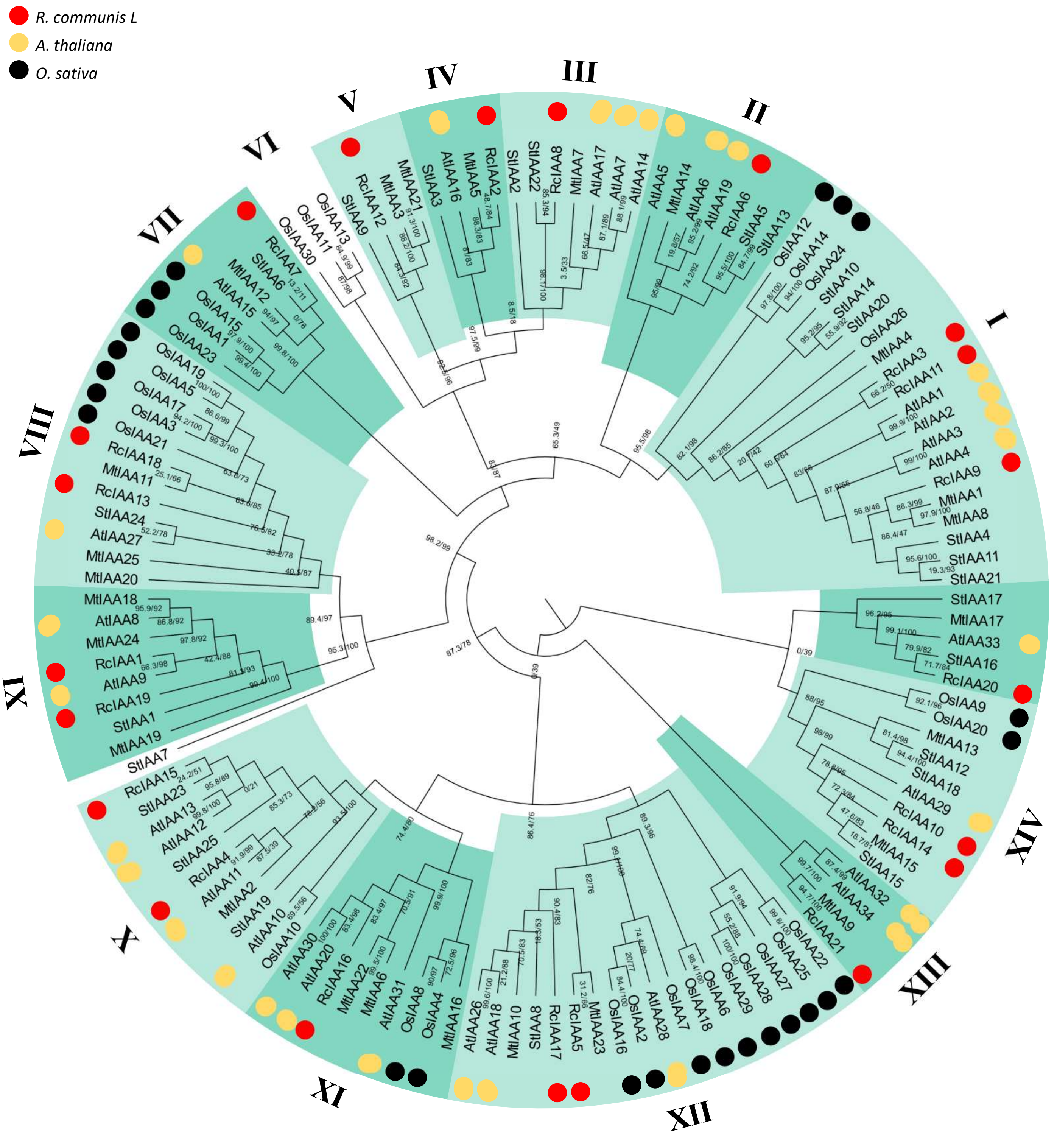

Figure S6. Maximum-likelihood phylogenetic analysis of IAA gene family. The phylogenetic relationships among analyzed proteins were reconstructed using the maximum likelihood method under the best model selection in Iqtree software (version 1.6.12) with 1000 replicates of bootstrap and ALRT statistics. Protein sequences from *R. communis, A. thaliana and O. sativa* are highlighted by circle red, yellow and black respectively. Sequences from *S. tuberosum* and *M. truncatula* were used to improve the analysis.

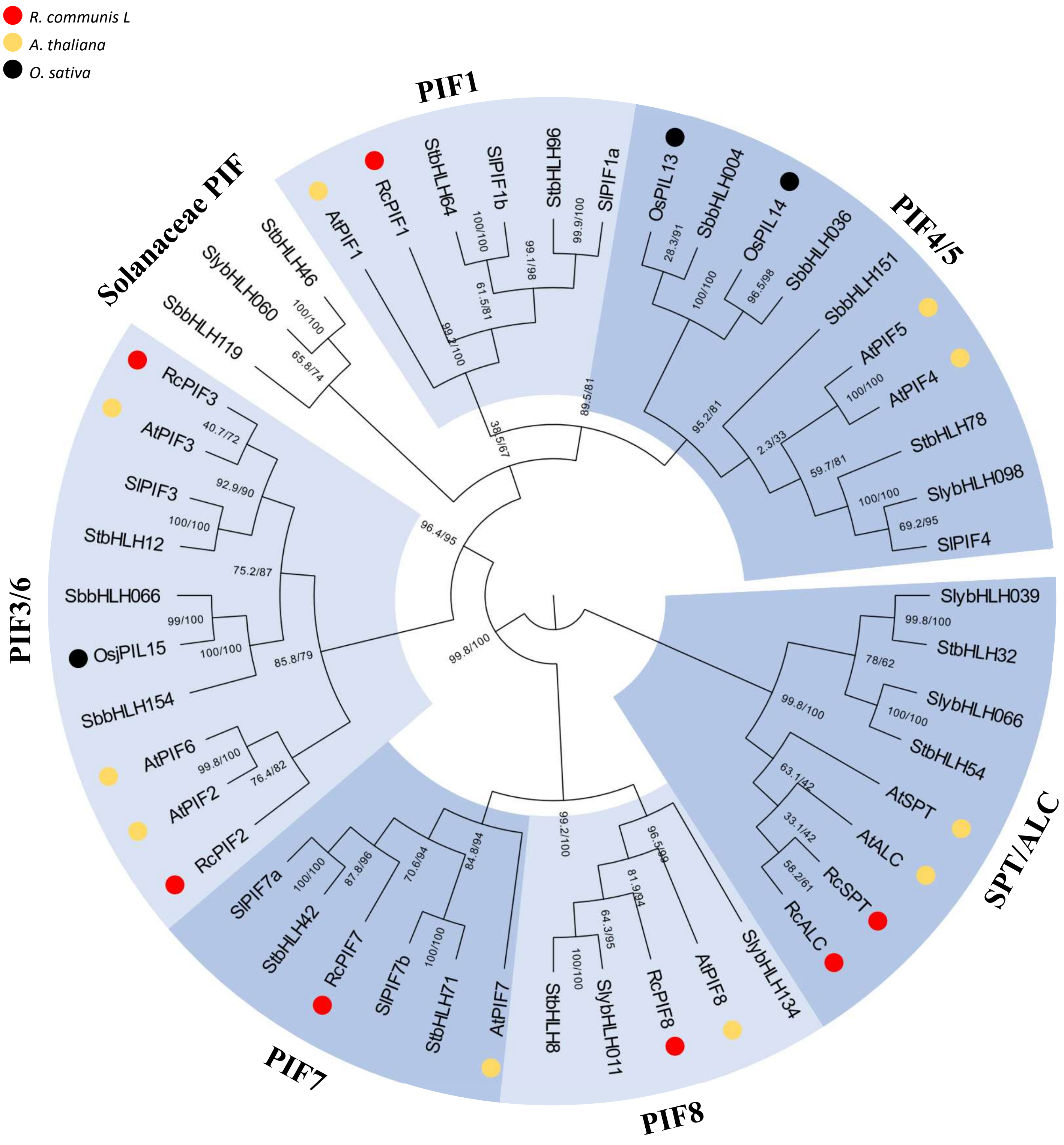

Figure S7. Maximum-likelihood phylogenetic analysis of PIF gene family. The phylogenetic relationships among analyzed proteins were reconstructed using the maximum likelihood method under the best model selection in Iqtree software (version 1.6.12) with 1000 replicates of bootstrap and ALRT statistics. Protein sequences from *R. communis, A. thaliana and O. sativa* are highlighted by circle red, yellow and black respectively. Sequences from *S. lycopersicum, S. tuberosum,* and *S. bicolor* were used to improve the analysis.

Figure S8

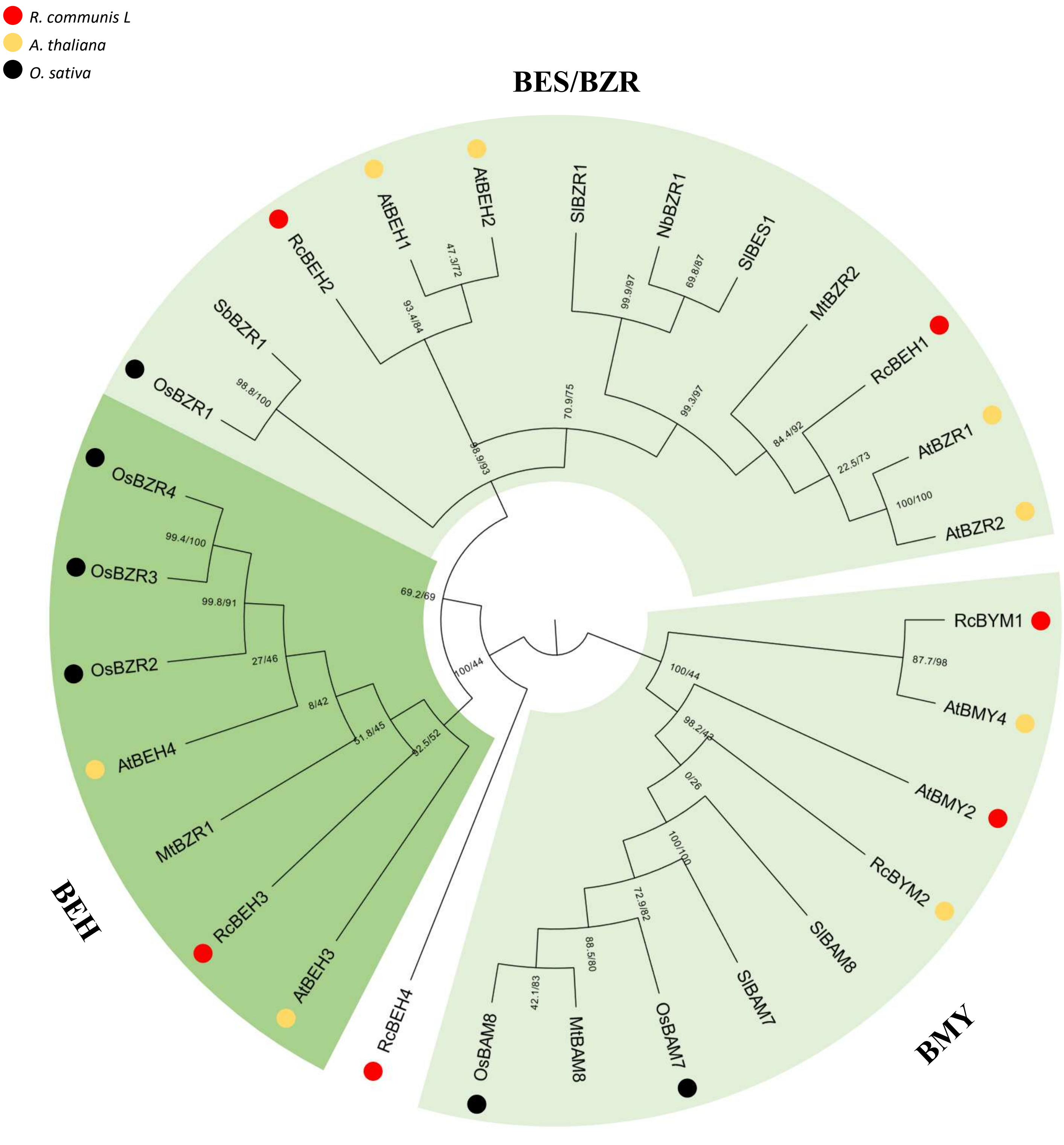

Figure S8. Maximum-likelihood phylogenetic analysis of BEH gene family. The phylogenetic relationships among analyzed proteins were reconstructed using the maximum likelihood method under the best model selection in Iqtree software (version 1.6.12) with 1000 replicates of bootstrap and ALRT statistics. Protein sequences from *R. communis, A. thaliana and O. sativa* are highlighted by circle red, yellow and black respectively. Sequences from *S. lycopersicum, N. benthamiana,* and *M. truncatula* were used to improve the analysis.

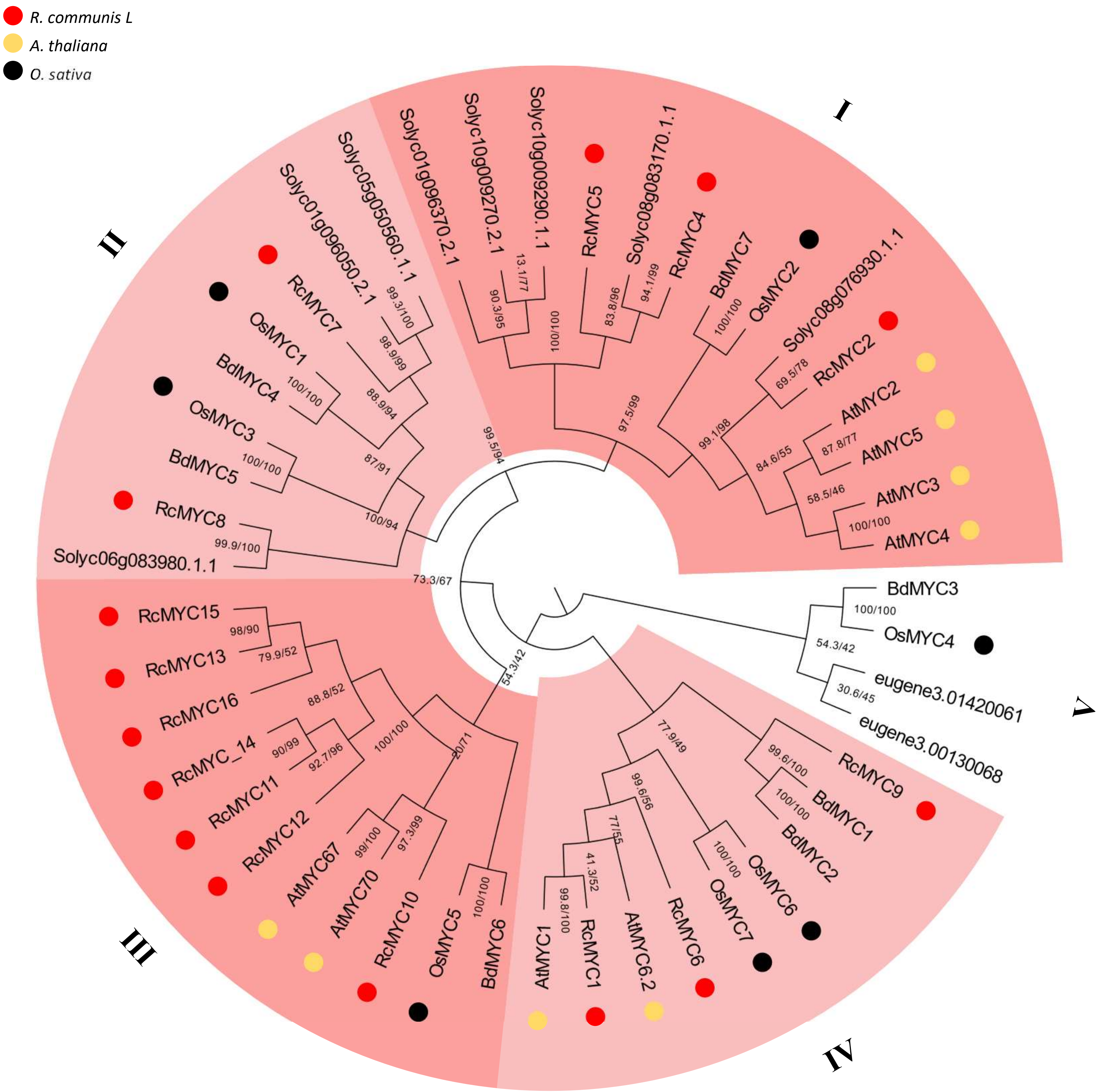

Figure S9. Maximum-likelihood phylogenetic analysis of MYC gene family. The phylogenetic relationships among analyzed proteins were reconstructed using the maximum likelihood method under the best model selection in Iqtree software (version 1.6.12) with 1000 replicates of bootstrap and ALRT statistics. Protein sequences from *R. communis, A. thaliana and O. sativa* are highlighted by circle red, yellow and black respectively. Sequences from *P. trichocarpa, S. lycopersicum,* and *B. distachyon* were used to improve the analysis.

Figure S10

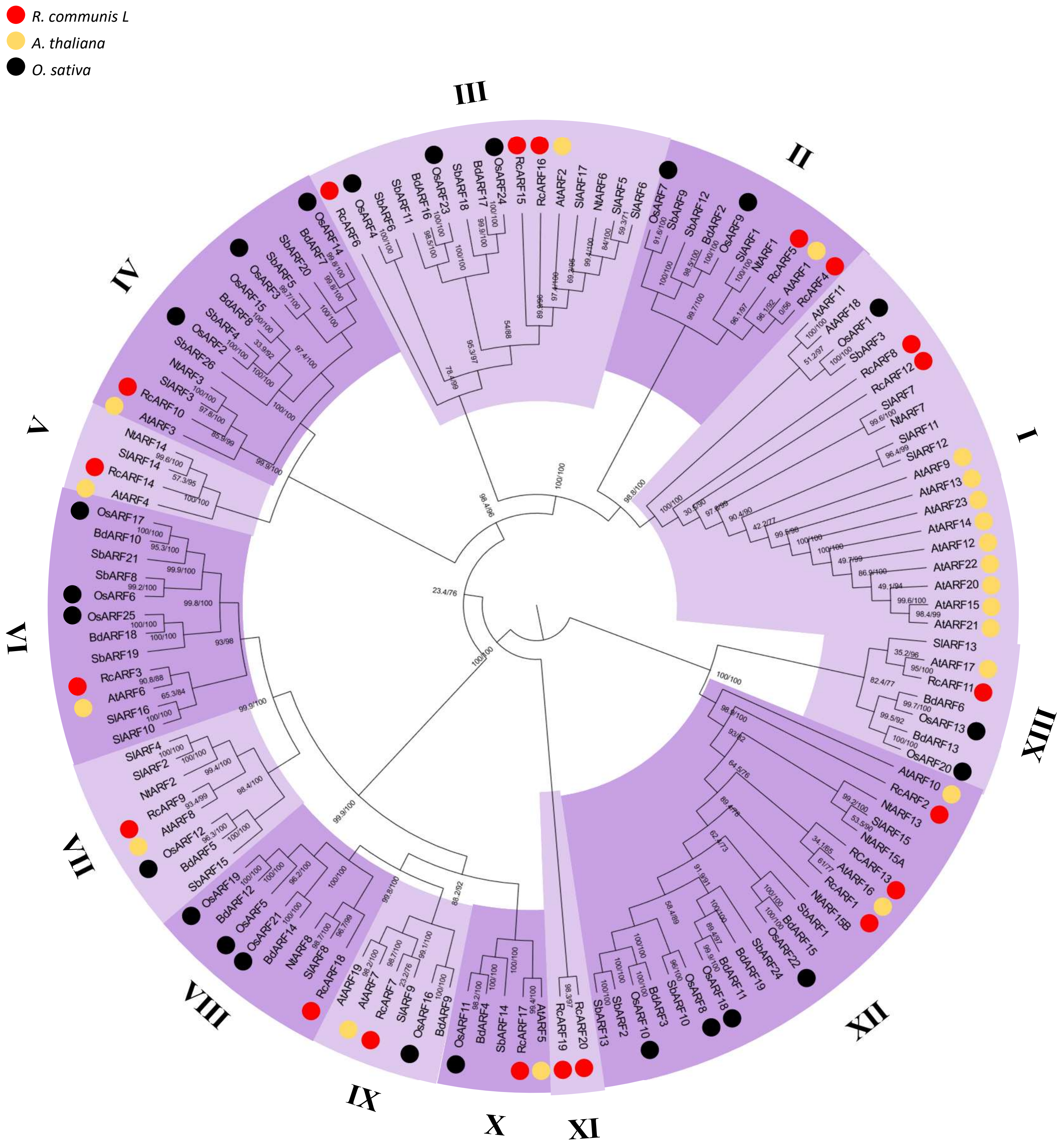

Figure S10. Maximum-likelihood phylogenetic analysis of ARF gene family. The phylogenetic relationships among analyzed proteins were reconstructed using the maximum likelihood method under the best model selection in Iqtree software (version 1.6.12) with 1000 replicates of bootstrap and ALRT statistics. Protein sequences from *R. communis, A. thaliana and O. sativa* are highlighted by circle red, yellow and black respectively. Sequences from *S. lycopersicum, N. tabacum, B. distachyon* and *S. bicolor* were used to improve the analysis.

Figure S11

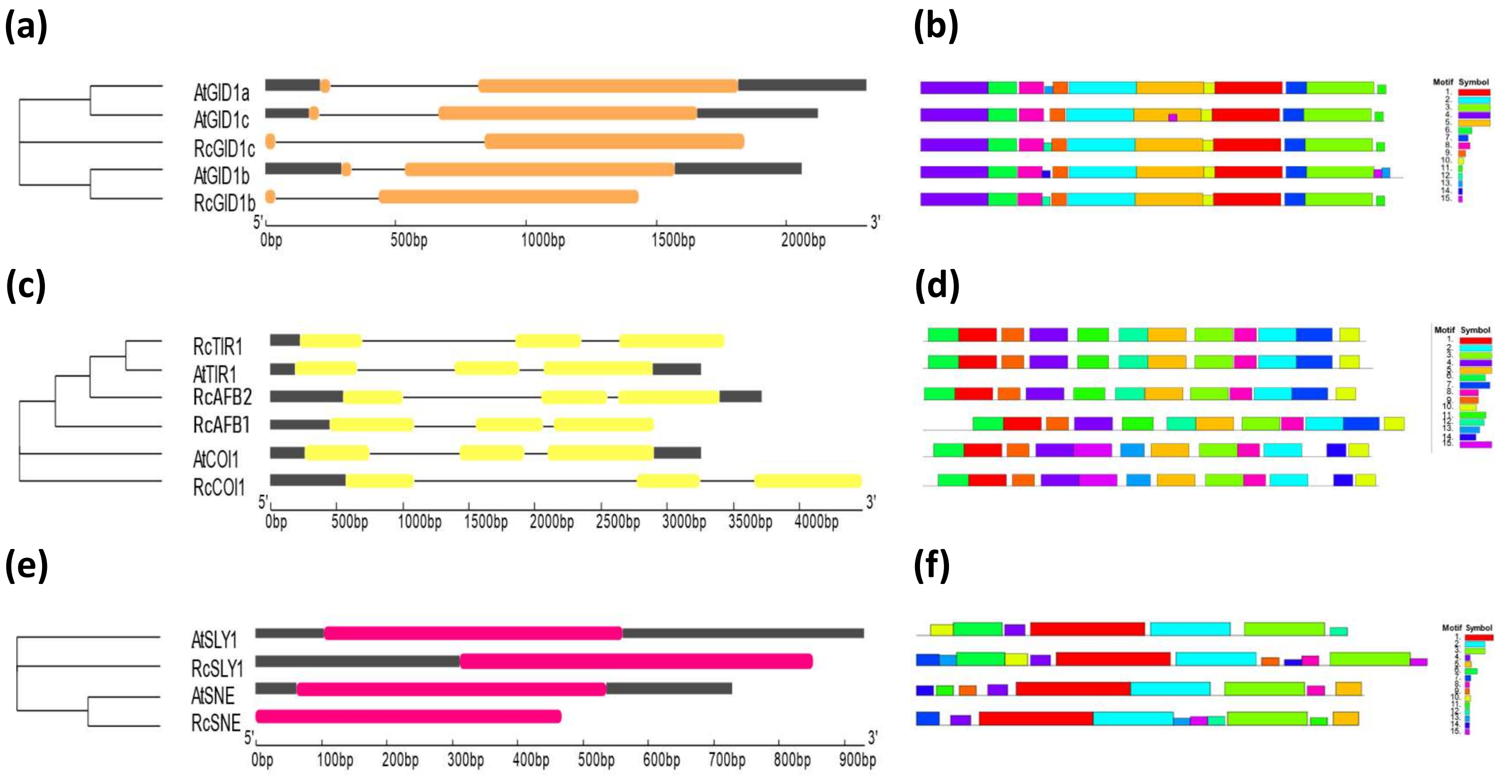

Figure S11. Analysis of the exon-intron structure and conserved motifs of receptors GID1, COI1/TIR1, and SLY/SNE. GID1 (A, B), COI1/TIR1 (C, D), and SLY/SNE (E, F) families in *R. communis* and *A. thaliana.* The phylogenetic relationships among analyzed proteins were reconstructed using the maximum likelihood method under the best model selection in MEGA 11 software with 1000 replicates of bootstrap statistics (panels A, C, and E). For all genes, black lines represent introns and colors box the exons (A, C, and E). All 15 conserved protein motifs are indicated by colored box and the lines represent non-conserved sequences.

Figure S12

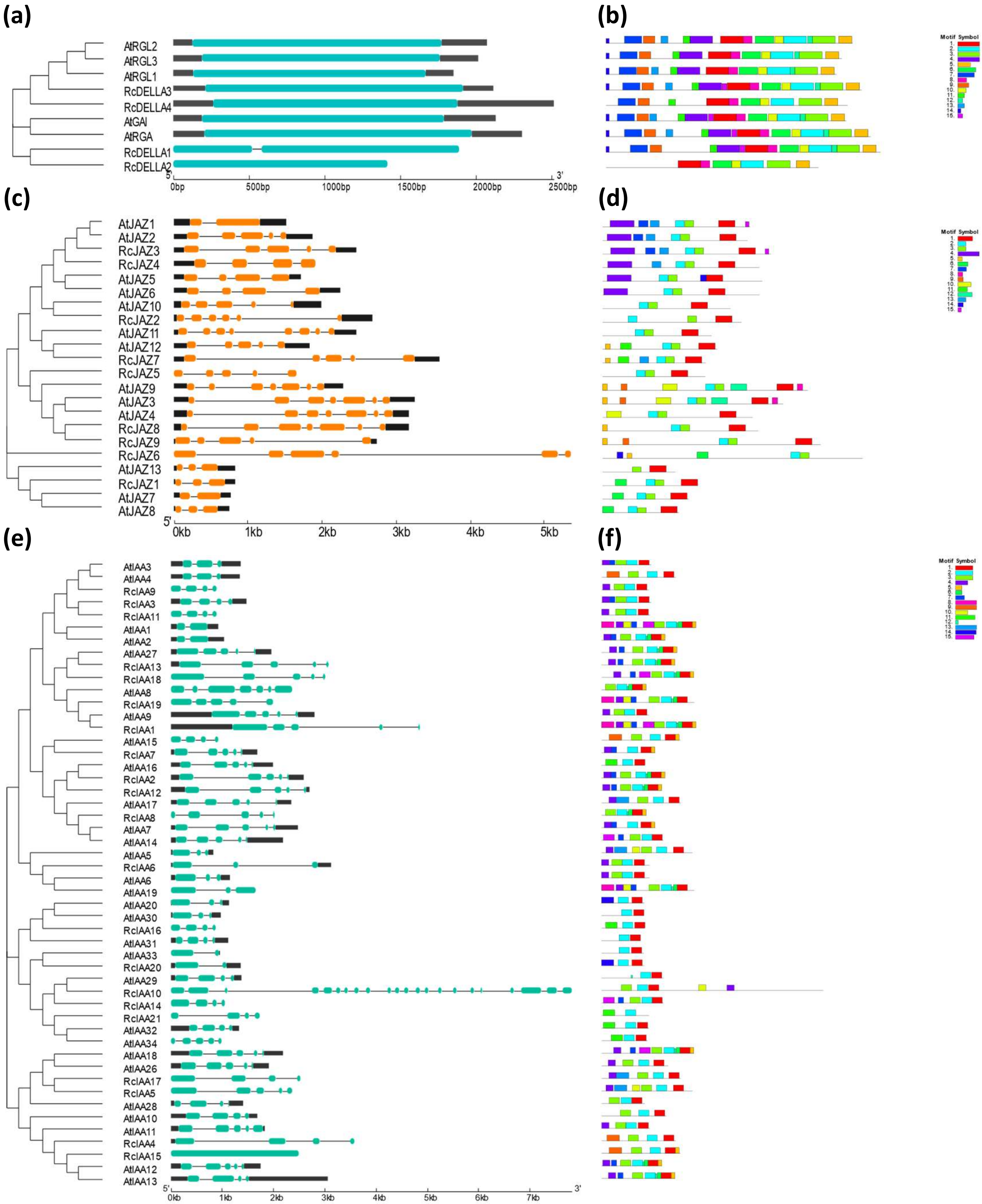
(a)
AtRGL2
AtRGL3
AtRGL1
RcDELLA3
RcDELLA4
AtGAI
AtRGA
RcDELLA1
RcDELLA2
5'
3'
0bp
500bp
1000bp
1500bp
2000bp
2500bp
(b)
Motif Symbol
(c)
AtJAZ1
AtJAZ2
RcJAZ3
RcJAZ4
AtJAZ5
AtJAZ6
AtJAZ10
RcJAZ2
AtJAZ11
AtJAZ12
RcJAZ7
RcJAZ5
AtJAZ9
AtJAZ3
AtJAZ4
RcJAZ8
RcJAZ9
RcJAZ6
AtJAZ13
RcJAZ1
AtJAZ7
AtJAZ8
5'
0kb
1kb
2kb
3kb
4kb
5kb
(d)
Motif Symbol
(e)
AtIAA3
AtIAA4
RcIAA9
RcIAA3
RcIAA11
AtIAA1
AtIAA2
AtIAA27
RcIAA13
RcIAA18
AtIAA8
RcIAA19
AtIAA9
RcIAA1
AtIAA15
RcIAA7
AtIAA16
RcIAA2
RcIAA12
AtIAA17
RcIAA8
AtIAA7
AtIAA14
AtIAA5
RcIAA6
AtIAA6
AtIAA19
AtIAA20
AtIAA30
RcIAA16
AtIAA31
AtIAA33
RcIAA20
AtIAA29
RcIAA10
RcIAA14
RcIAA21
AtIAA32
AtIAA34
AtIAA18
AtIAA26
RcIAA17
RcIAA5
AtIAA28
AtIAA10
AtIAA11
RcIAA4
RcIAA15
AtIAA12
AtIAA13
5'
3'
0kb
1kb
2kb
3kb
4kb
5kb
6kb
7kb
(f)
Motif Symbol

Figure S12. Analysis of the exon-intron structure and conserved motifs of inhibitor proteins DELLA, JAZ, and IAA. DELLA (A, B), JAZ (C, D), and IAA (E, F) families in *R. communis* and *A. thaliana.* For all genes, black lines represent introns and colors box the exons (A, C, and E). The phylogenetic relationships among analyzed proteins were reconstructed using the maximum likelihood method under the best model selection in MEGA 11 software with 1000 replicates of bootstrap statistics (panels A, C, and E). All 15 conserved protein motifs are indicated by colored box and the lines represent non-conserved sequences.

Figure S13

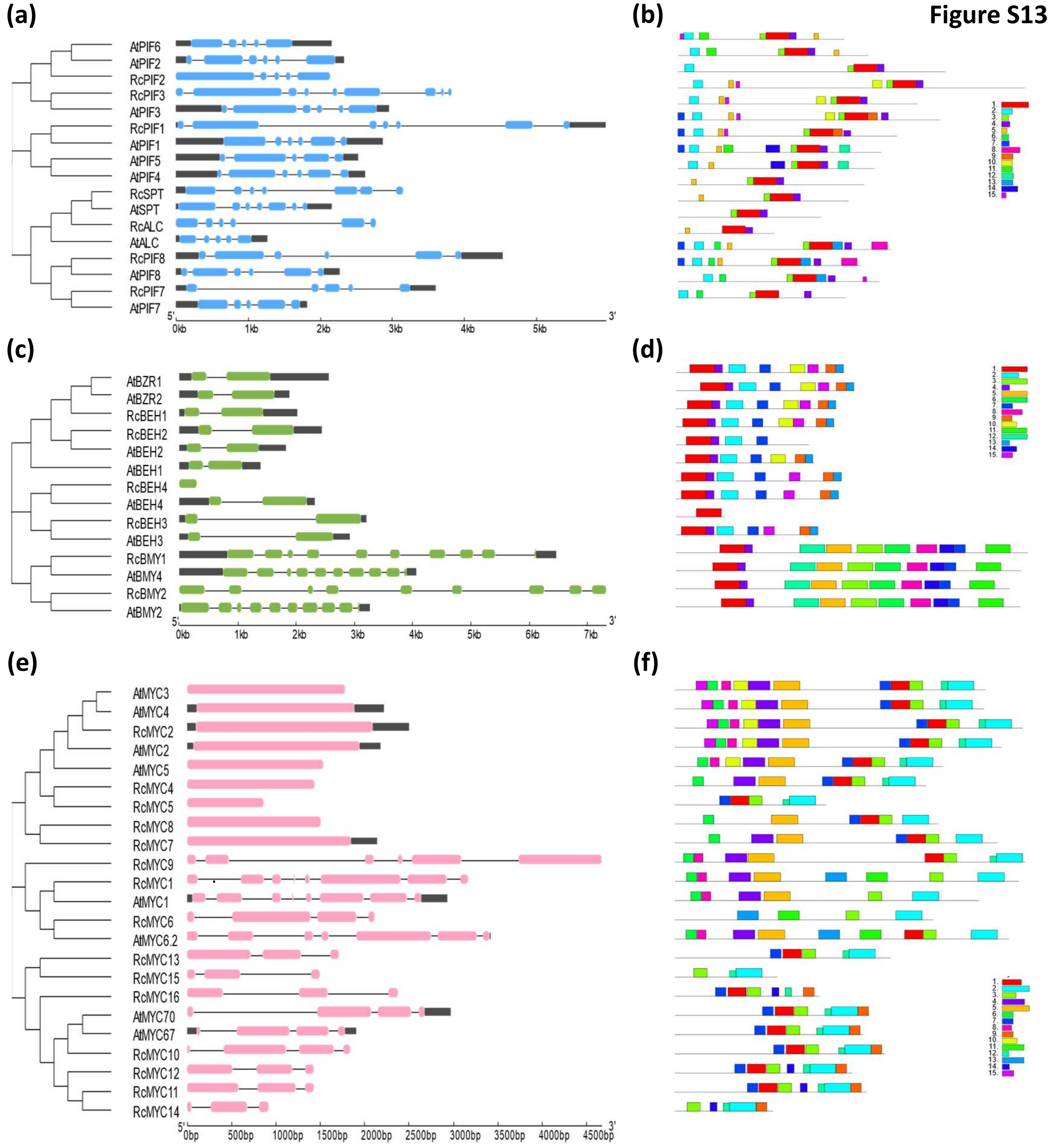

Figure S13. Analysis of the exon-intron structure and conserved motifs of transcription factors PIF, BEH, and MYC. PIF (A, B), BEH (C, D) and MYC (E, F) families in *R. communis* and *A. thaliana.* The phylogenetic relationships among analyzed proteins were reconstructed using the maximum likelihood method under the best model selection in MEGA 11 software with 1000 replicates of bootstrap statistics (panels A, C, and E). All 15 conserved protein motifs are indicated by colored box and the lines represent non-conserved sequences.

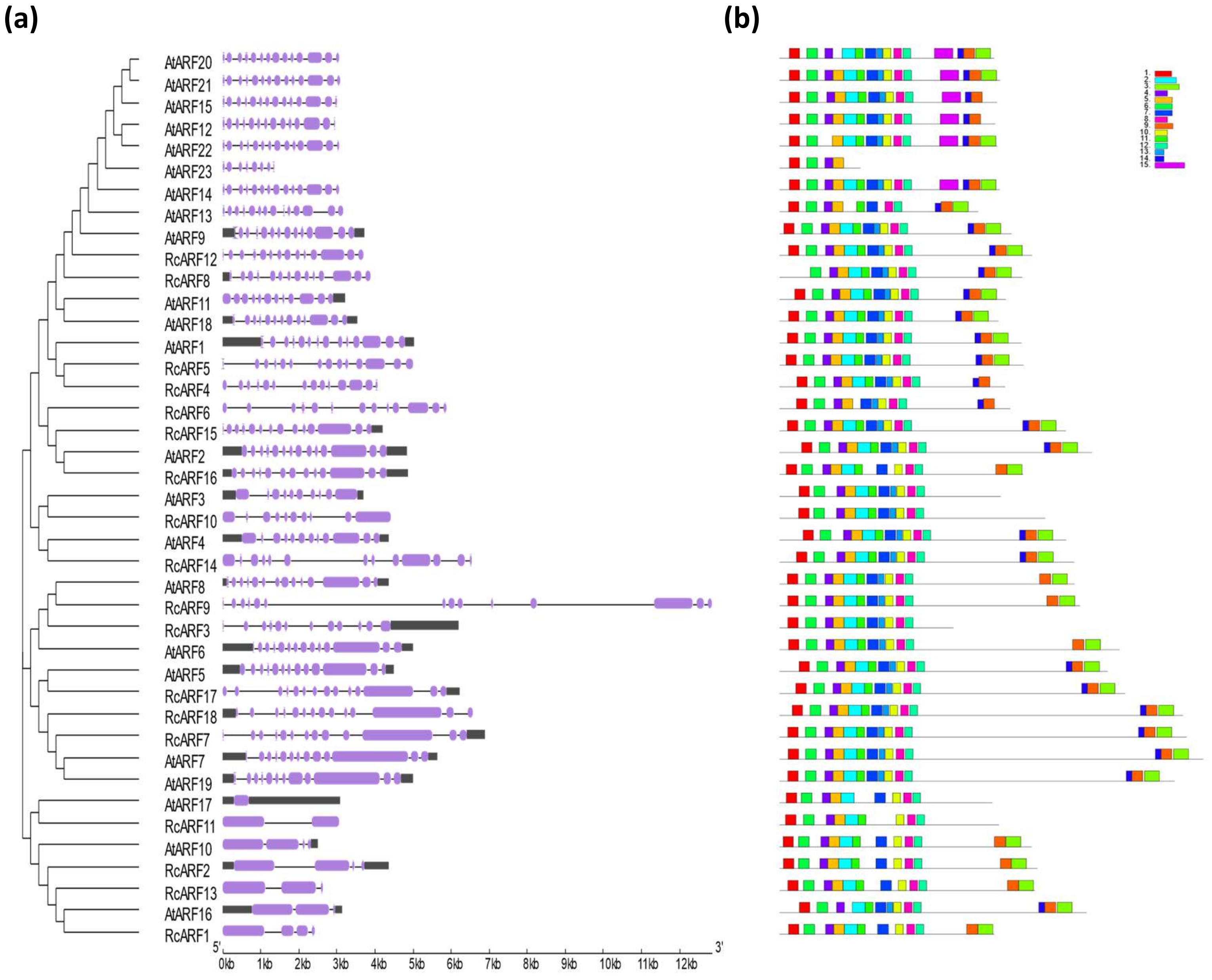

Figure S14. Analysis of the exon-intron structure and conserved motifs of the transcription factor ARF. ARF (A, B), family in *R. communis* and *A. thaliana.* For all genes, black lines represent introns and colors box the exons (a). The phylogenetic relationships among analyzed proteins were reconstructed using the maximum likelihood method under the best model selection in MEGA 11 software with 1000 replicates of bootstrap statistics (panels A, C, and E). All 15 conserved protein motifs are indicated by colored box and the lines represent non-conserved sequences.
